# Sex differences in physical fitness among 10,000 adolescents aged 13–15 years

**DOI:** 10.1371/journal.pone.0345291

**Published:** 2026-03-20

**Authors:** Ali Gorzi, Hamid Rajabi, Mina Khantan, Tommy R. Lundberg

**Affiliations:** 1 Department of Sport Sciences, University of Zanjan, Zanjan, Iran; 2 Division of Clinical Physiology, Department of Laboratory Medicine, Karolinska Institutet, Stockholm, Sweden; 3 Department of Sport Sciences, Kharazmi University, Tehran, Iran; 4 Faculty of Sport Sciences and Health, Shahid Beheshti University, Tehran, Iran; 5 Unit of Clinical Physiology, Karolinska University Hospital, Stockholm, Sweden; San Raffaele University of Rome, ITALY

## Abstract

Physical fitness during adolescence is critical for health and sports participation, with sex-specific developmental trajectories influencing performance. The aim of this cross-sectional study was to examine sex differences in physical fitness among non-athletic adolescents aged 13–15 years and to provide reference values for fitness parameters across age and sex. We assessed 9,669 non-athletic adolescents (64% females) aged 13–15 years. Fitness tests included Sargent jump, standing long jump, 30m sprint, medicine ball chest throw, and 6-minute shuttle run. Interactions between sex and age were analyzed using two-way ANOVA, with effect sizes (Cohen’s *d*) and mean differences calculated between 13–15 years of age. Pearson correlation coefficients were used to examine relevant relationships, and were compared between sexes using Fisher’s r-to-z transformation. Significant sex-by-age interactions were observed for all fitness parameters (p < 0.001). Boys showed greater differences than girls from 13 to 15 years, with mean differences for Sargent jump (7.0 vs. 1.6 cm), standing long jump (28 vs. 7 cm), 30m sprint (−0.54 vs. −0.01 s), medicine ball throw (1.6 m vs. 0.4 m), and 6-minute shuttle run (2.0 vs. −0.3 laps). Height correlated moderately with the power-based tests in both sexes (p < 0.001), particularly in boys (R = 0.21 to 0.56 depending on age and test), but not with endurance. Correlations between tests were stronger (p < 0.01) in boys for all comparisons except medicine ball throw vs. shuttle run. We conclude that boys show larger fitness differences from 13 to 15 years of age than girls, likely due to pubertal changes that increase stature and improve muscle mass and body composition. These reference values serve as a basis for sex-specific interventions to improve adolescent health, performance, and sports participation.

## Introduction

Adolescence, particularly the ages of 13–15, is a critical period marked by rapid psychological and biological changes that significantly impact sport participation, physical fitness and athletic performance [1,2]. Boys experience performance-enhancing changes due to increased testosterone levels, leading to greater muscle mass, bone density, and cardiovascular capacity [3]. In contrast, girls mature earlier, often accumulating more fat mass and reaching their peak height velocity (largest growth rate in cm/year) around age 12, about two years before boys [4]. Additionally, girls may face challenges related to breast development, menstruation, and iron status, which can affect their performance [5]. These physiological differences result in boys generally outperforming girls in most fitness measures except flexibility [6–8]. A study on 2267 Austrian adolescents (mixed training status) aged 11–17 found that after the age of 13, fitness improvements plateaued for girls whereas boys continued to improve in most areas and performed better than girls in tests of speed, power, strength, endurance, and agility, while girls had better flexibility [9].

These biological sex differences occur during a period when sports dropout rates are substantially higher among girls than boys, with factors such as low self-confidence, negative body image, and perceived lack of skill among the key contributors [10–15]. It is possible that experiencing minimal fitness improvements while observing peers’ rapid gains may negatively affect adolescents’ perceived physical competence [5,16]. However, characterizing the actual magnitude of sex differences in fitness development during this period, particularly in non-athletic adolescents where training effects are removed, is a prerequisite for understanding whether such disparities exist at the population level. Studying non-athletes specifically isolates developmental patterns from training effects, providing baseline data on maturational changes in fitness that may be relevant to broader discussions of physical activity engagement.

Previous research has established sex- and age-specific reference values for physical fitness in children and adolescents, including large-scale European normative studies [17,18]. These studies indicate that boys’ fitness improves more than girls’ during adolescence. However, existing work has typically reported descriptive values rather than directly testing the statistical magnitude of sex differences across age groups using formal interaction analyses and standardized effect sizes. Furthermore, previous normative studies have focused primarily on health-related applications, with limited attention to how the observed patterns might inform discussions of perceived competence and sports participation. Many studies also include populations of mixed training status [9,17], focus on single outcome measures [19], or examine ages outside the critical 13–15 year window [20,21]. A systematic review highlighted additional gaps including inconsistent testing methods and non-representative samples [22]. The present study addresses these limitations by providing a formal quantitative comparison of sex-specific developmental trajectories, using effect sizes with confidence intervals and interaction testing, in a large sample of exclusively non-athletic adolescents from Iran, a population underrepresented in the fitness literature. This approach isolates the effects of natural maturation from training influences and provides reference data that may inform future research on the relationship between developmental fitness patterns and physical activity engagement.

Accordingly, we examined sex differences in physical fitness parameters among 10,000 non-athletic adolescents aged 13–15 years. This large-scale analysis provides useful reference values for various fitness components, including strength, speed, endurance, and power, and helps to establish the magnitude of sex differences during these ages. We hypothesized that boys would show greater differences in fitness parameters across ages compared to girls, with different magnitudes across performance metrics.

## Materials and methods

### Study design and participants

This cross-sectional study assessed physical fitness in close to 10,000 non-athletic adolescents aged 12.5–15.5 years (grouped as age groups 13, 14, and 15 years) from 6 cities across 3 provinces in Iran. Participants were recruited from middle schools using a stratified sampling approach to ensure geographic representation. After excluding outliers (unlrealistic values/manual input errors) and incomplete data, 9669 participants remained (6176 girls, 3493 boys). Exclusion criteria included regular and structured athletic training in the past 6 months, reported medical conditions, or failure to complete at least 3 of 6 fitness tests (327 for girls and 93 for boys were excluded due to failure to complete at least three tests). The lower number of boys in the study primarily reflects the exclusion criteria, since a greater proportion of boys reported regular participation in sports activities. A six-month period without structured training was considered a conservative threshold for classifying participants as non-athletes. This duration substantially exceeds the timeframes over which training-induced physiological adaptations have been reported to dissipate in the literature, even when most available data are from adult populations [23]. Furthermore, any residual training effects among participants who stopped training close to this threshold would be expected to distribute equally across age and sex groups, and would therefore not systematically bias the between-group comparisons that are the focus of this study. For girls, testing was scheduled outside their menstrual period based on self-reported cycle timing.

The retrospective study was approved by the Research Ethics Committees of Kharazmi University (Code: IR.KHU.REC.1403.165), Tehran, Iran, and complied with ethical standards outlined in the Declaration of Helsinki (except pre-registration). Written informed consent was obtained from participants and their parents regarding the timing and type of tests, potential considerations, and the publication of overall results (01.03.2017–15.03.2017).

### Inclusivity in global research

Additional information regarding the ethical, cultural, and scientific considerations specific to inclusivity in global research is included in the Supporting Information (S4 File)

### Anthropometry and physical fitness assessments

Height and five field-based fitness tests, adapted mainly from the Eurofit battery [24], were conducted in the following order: height, standing long jump, Sargent jump, 30 m sprint, medicine ball chest throw, and 6-minute shuttle run. Tests were performed between 9:00 and 12:00 on a single day in school gymnasiums with standardized flooring, separately for boys and girls. All examiners participated in a centralized training workshop prior to data collection to ensure consistent measurement procedures. Participants completed a 5-minute dynamic warm-up before testing. Teachers and trained staff provided standardized instructions and encouragement to ensure maximal effort. Each test (except the shuttle run) allowed 3 trials with 2–3 minutes of rest between attempts, and the best result was recorded. Although intra- and inter-rater reliability were not directly evaluated in this study, the physical fitness tests employed are part of standardized battery with previously reported high test–retest reliability [25–29]. All tests were administered in the same order for all participants.

Height: Measured without shoes using a wall-mounted stadiometer to the nearest 0.1 cm.

Standing Long Jump: Participants stood behind a marked line and jumped forward with arm swing allowed, measured to the nearest 1 cm.

Sargent Jump: Participants stood against a wall, marked their standing reach height, then jumped vertically with bent knees and arms lowered, aiming to touch the highest point. Jump height was measured to the nearest 1 cm.

30 m Sprint: Participants ran 30 m from a standing start in groups of 3, randomized by class, with time recorded from the staff member’s command (including reaction time) using a stopwatch to the nearest 0.01 s.

Medicine Ball Chest Throw: Participants threw a 1 kg (girls) or 2 kg (boys) medicine ball from a 50 cm² marked area, using a chest-pass technique with lower body engagement, parallel feet, and facing the throwing line. Distance was measured to the nearest 1 cm.

6-Minute Shuttle Run: Participants ran 20 m laps for 6 minutes, self-paced, with the total number of laps recorded manually by two staff members.

### Statistical analysis

Percentiles were calculated for all tests by age group and sex. A two-way ANOVA was used to examine the effects of sex (male, female) and age (13, 14, 15 years) on fitness outcomes, with a focus on sex-by-age interactions. Effect sizes (Cohen’s *d*) with 95% confidence intervals (CI) were calculated for pairwise comparisons between 13 and 15 years, along with corresponding raw mean differences and 95% CI. Normality and homogeneity of variance were assessed using Shapiro-Wilk and Levene’s tests, respectively. Pearson correlations evaluated relationships between height and fitness scores, as well as between-fitness test relationships. To determine whether the relationships between physical fitness parameters differed significantly between boys and girls (averaged across all age groups), we formally compared correlation coefficients using Fisher’s r-to-z transformation [30]. The difference between z-scores (z₁ - z₂) was tested against the null hypothesis that the population correlation coefficients are equal (H₀: ρ₁ = ρ₂) using a two-tailed test with α = 0.05. The test statistic was calculated as z_diff/SE_diff, where SE_diff = √(SE₁² + SE₂²) and SE = 1/√(n – 3) for each group. Confidence intervals (95%) for the difference in correlation coefficients were computed using the method proposed by Zou [31]. Data are presented as means ± SD or 95% CI unless stated otherwise. Statistical analyses were performed using JAMOVI (version 2.6.26.0) and R studio (Version 2025.09.0 + 387). Graphs were created in GraphPad Prism and R Studio.

## Results

Percentile tables for all tests by age group and sex are shown in Table 1.

**Table 1:** Sex- and age-specific Mean±SD, 95% CI lower and upper and percentiles.

Test	Sex	Age	95% Confidence Interval/ Percentiles		P10	P20	P30	P40	P50	P60	P70	P80	P90
			M ± SD	Lower-Upper									
**Height** (cm)	Girls	13 (n = 1113)	156.9 ± 6.4	156.5-157.2	148	152	154	155	157	159	160	162	165
		14 (n = 1982)	159.0 ± 6.1	158.7-159.3	151	154	156	158	159	160	162	164	167
		15 (n = 3020)	160.9 ± 6.1	160.6-161.1	153	156	158	160	161	162,5	164	166	169
	Boys	13 (n = 512)	156.1 ± 8.0	155.4-156.8	145	150	152	154	156	158	160	162	167
		14 (n = 1057)	161.0 ± 8.8	160.5-161.6	150	153	156	159	161	163	165	169	172
		15 (n = 1914)	169.1 ± 8.0	168.7-169.5	159	163	165	168	170	171	174	176	180
**Sargent Jump** (cm)	Girls	13 (n = 1086)	26.4 ± 6.0	26.1-26.8	19	21	23	25	26	28	30	31	35
		14 (n = 1954)	27.2 ± 6.3	26.9-27.5	20	22	24	25	27	28,5	30	32	35
		15 (n = 2962)	28.1 ± 6.5	27.8-28.3	20	23	25	26	28	30	30	33	36
	Boys	13 (n = 512)	29.3 ± 7.1	28.7-29.9	21	24	25	27	29	30	33	35	39
		14 (n = 1055)	32.2 ± 7.6	31.7-32.6	23	25	28	30	32	34	36	39	43
		15 (n = 1915)	36.3 ± 8.6	35.9-36.7	25	30	31	34	35	39	40	43	48
**Standing Long Jump** (cm)	Girls	13 (n = 1128)	134.5 ± 22.0	133.2-135.8	108	115	120	128	133	140	145	153	164,3
		14 (n = 1984)	136.9 ± 23.4	135.9-137.9	110	118	123	130	135	140	150	158	168
		15 (n = 3014)	141.0 ± 23.9	140.1-141.8	111	120	129	134	140	145	150	160	170
	Boys	13 (n = 511)	159.1 ± 23.3	157.1-161.1	130	140	145	150	160	165	170	180	190
		14 (n = 1055)	169.9 ± 26.5	168.3-171.5	135	148	155	163,2	170	175	183	192	205
		15 (n = 1914)	187.3 ± 27.4	186.1-188.6	150	165	174	180	190	195	201,1	210	220
**30m Sprint** (s)	Girls	13 (n = 1102)	6.26 ± 0.74	6.22-6.31	5,34	5,65	5,89	6,06	6,21	6,36	6,58	6,84	7,22
		14 (n = 1926)	6.25 ± 0.77	6.22-6.29	5,35	5,62	5,84	6	6,15	6,34	6,53	6,84	7,23
		15 (n = 2956)	6.25 ± 0.76	6.22-6.28	5,31	5,61	5,84	6	6,16	6,34	6,56	6,82	7,22
	Boys	13 (n = 509)	5.81 ± 0.74	5.74-5.87	5	5,15	5,32	5,51	5,75	5,96	6,1	6,37	6,7
		14 (n = 1041)	5.57 ± 0.63	5.53-5.61	4,9	5,06	5,19	5,32	5,5	5,63	5,8	6,03	6,45
		15 (n = 1885)	5.27 ± 0.63	5.24-5.30	4,58	4,78	4,91	5,03	5,16	5,31	5,5	5,76	6,06
**Medicine ball Ches Throw** (m)	Girls	13 (n = 1115)	5.18 ± 0.83	5.13-5.23	4,1	4,5	4,75	5	5,1	5,36	5,6	5,9	6,28
		14 (n = 1970)	5.38 ± 0.85	5.34-5.42	4,3	4,7	5	5,1	5,3	5,52	5,8	6	6,5
		15 (n = 2999)	5.59 ± 0.88	5.56-5.62	4,5	4,9	5,1	5,35	5,55	5,8	6	6,3	6,69
	Boys	13 (n = 510)	5.12 ± 0.97	5.04-5.21	4	4,3	4,5	4,79	5	5,2	5,5	5,8	6,26
		14 (n = 1049)	5.70 ± 1.16	5.63-5.77	4,3	4,7	5	5,3	5,5	5,9	6,15	6,6	7,2
		15 (n = 1909)	6.68 ± 1.37	6.62-6.74	5	5,5	6	6,2	6,5	7	7,3	7,7	8,5
**6 min Shuttle Run** (round)	Girls	13 (n = 1058)	19.37 ± 3.35	19.1-19.5	15	17	18	19	19,5	20	21	22	23
		14 (n = 1814)	19.07 ± 3.41	18.9-19.2	15	16,75	17,75	18,5	19	20	20,5	21,5	23
		15 (n = 2744)	19.07 ± 3.51	18.9-19.2	15	17	18	18,5	19	20	20,5	21,75	23
	Boys	13 (n = 478)	21.48 ± 4.86	21.-21.9	14,35	18	19	20,5	22	23	24	25,5	27
		14 (n = 983)	22.74 ± 4.68	22.4-23.0	17	19	21	22	23	24	25,5	27	28
		15 (n = 1770)	23.46 ± 5.19	23.2-23.7	17	19	21	22,5	24	25	26	28	30

Note: Different Sample size for the same age-sex group (e.g., 1113 N for G13 in Height vs. 1086 G13 for Sargent Jump) is due to incomplete test participation.

### Height

**Fig 1:**
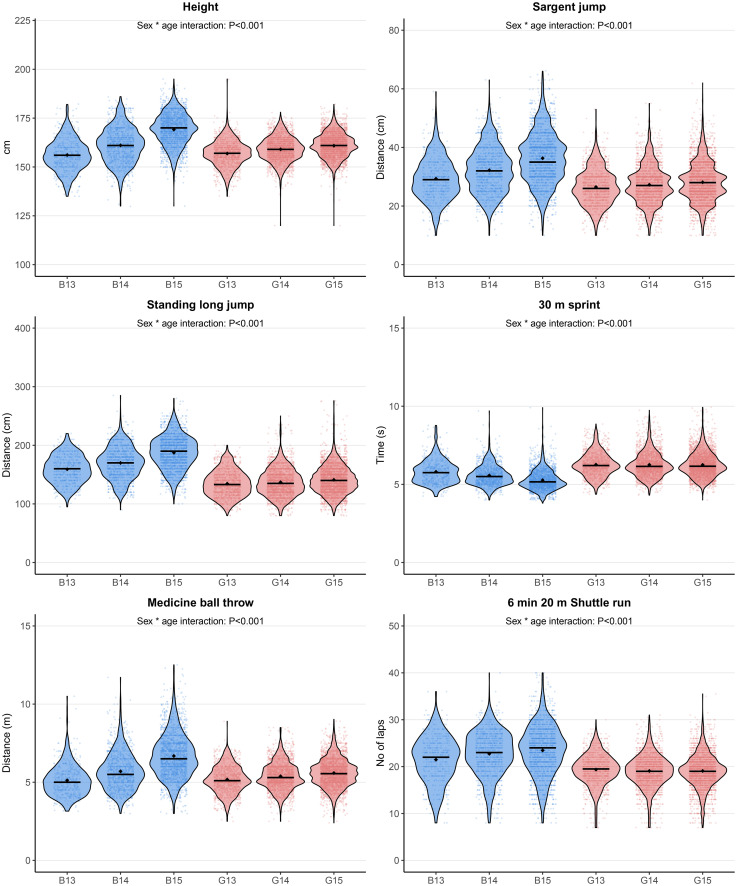
Mean, SD and distribution pattern of Height, Standing long jump, Sargent jump, 30 m sprint, Medicine ball throw and 6 min shuttle run. B = Boys, G = Girls.

**Fig 2:**
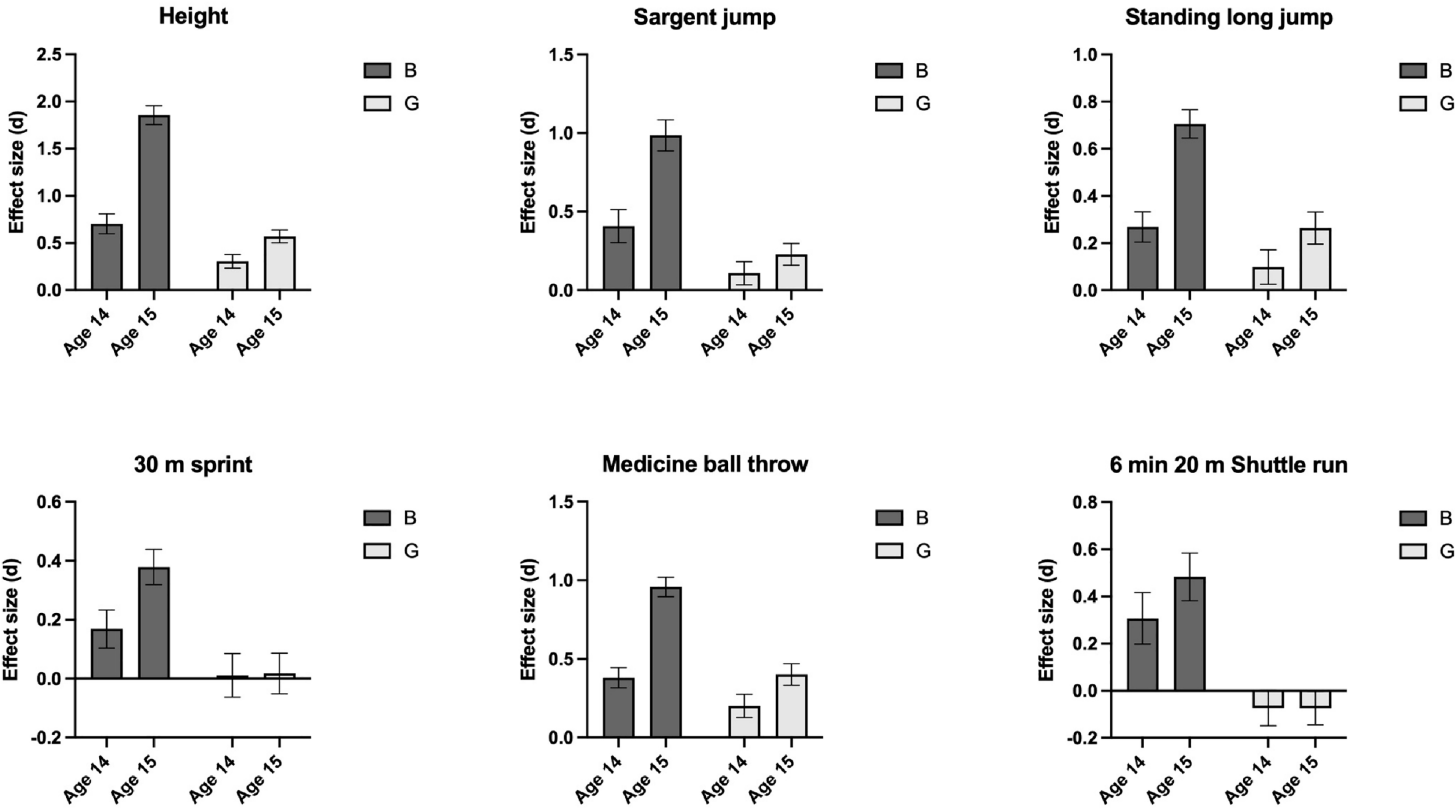
Effect size (Cohen’s *d*) differences of Height, Standing long jump, Sargent jump, 30 m sprint, Medicine ball throw and 6 min shuttle run. Age 13 is used as the reference age group in this analysis. B = Boys, G = Girls.

There was an interaction between sex and age in height (F = 306; p < 0.001; Fig 1). This was due to a larger difference between age groups in boys compared to girls. For example, the effect size of the difference between 13 and 15 years was 1.86 (95% CI 1.75, 1.96) for boys compared to 0.57 (95% CI 0.51, 0.64) for girls (Fig 2; Table 2).

**Table 2:** Sex-specific age comparisons.

Outcome variable	Sex	Comparison	Mean at Age 13	Mean at comparison Age group	Mean Difference	CI Lower	CI Upper	95% CI	N Age 13	N comparator group
Height (cm)	Boys	Age 14 vs Age 13	156,12	161,06	4,94	4,06	5,82	[4.06, 5.82]	512	1057
Height (cm)	Boys	Age 15 vs Age 13	156,12	169,14	13,02	12,24	13,8	[12.24, 13.8]	512	1914
Height (cm)	Girls	Age 14 vs Age 13	156,89	159,03	2,14	1,67	2,61	[1.67, 2.61]	1113	1982
Height (cm)	Girls	Age 15 vs Age 13	156,89	160,89	4	3,56	4,44	[3.56, 4.44]	1113	3020
Sargent jump (cm)	Boys	Age 14 vs Age 13	29,32	32,21	2,89	2,12	3,66	[2.12, 3.66]	512	1055
Sargent jump (cm)	Boys	Age 15 vs Age 13	29,32	36,31	6,99	6,27	7,72	[6.27, 7.72]	512	1915
Sargent jump (cm)	Girls	Age 14 vs Age 13	26,49	27,26	0,76	0,31	1,22	[0.31, 1.22]	1086	1954
Sargent jump (cm)	Girls	Age 15 vs Age 13	26,49	28,11	1,62	1,19	2,05	[1.19, 2.05]	1086	2962
Standing long jump (cm)	Boys	Age 14 vs Age 13	159,13	169,95	10,82	8,24	13,4	[8.24, 13.4]	511	1055
Standing long jump (cm)	Boys	Age 15 vs Age 13	159,13	187,37	28,24	25,88	30,61	[25.88, 30.61]	511	1914
Standing long jump (cm)	Girls	Age 14 vs Age 13	134,52	136,94	2,42	0,77	4,07	[0.77, 4.07]	1128	1984
Standing long jump (cm)	Girls	Age 15 vs Age 13	134,52	141,02	6,5	4,96	8,04	[4.96, 8.04]	1128	3014
30 m sprint (s)	Boys	Age 14 vs Age 13	5,81	5,57	−0,24	−0,31	−0,16	[-0.31, -0.16]	509	1041
30 m sprint (s)	Boys	Age 15 vs Age 13	5,81	5,27	−0,54	−0,61	−0,47	[-0.61, -0.47]	509	1885
30 m sprint (s)	Girls	Age 14 vs Age 13	6,26	6,25	−0,01	−0,06	0,05	[-0.06, 0.05]	1102	1926
30 m sprint (s)	Girls	Age 15 vs Age 13	6,26	6,25	−0,01	−0,06	0,04	[-0.06, 0.04]	1102	2956
Medicine ball throw (m)	Boys	Age 14 vs Age 13	5,12	5,7	0,57	0,46	0,68	[0.46, 0.68]	510	1049
Medicine ball throw (m)	Boys	Age 15 vs Age 13	5,12	6,68	1,56	1,45	1,66	[1.45, 1.66]	510	1909
Medicine ball throw (m)	Girls	Age 14 vs Age 13	5,18	5,38	0,21	0,14	0,27	[0.14, 0.27]	1115	1970
Medicine ball throw (m)	Girls	Age 15 vs Age 13	5,18	5,59	0,41	0,35	0,47	[0.35, 0.47]	1115	2999
6 min shuttle run (laps)	Boys	Age 14 vs Age 13	21,48	22,74	1,25	0,73	1,78	[0.73, 1.78]	478	983
6 min shuttle run (laps)	Boys	Age 15 vs Age 13	21,48	23,46	1,98	1,48	2,47	[1.48, 2.47]	478	1770
6 min shuttle run (laps)	Girls	Age 14 vs Age 13	19,37	19,07	−0,3	−0,55	−0,04	[-0.55, -0.04]	1058	1814
6 min shuttle run (laps)	Girls	Age 15 vs Age 13	19,37	19,07	−0,3	−0,54	−0,06	[-0.54, -0.06]	1058	2744

### Sargent jump

There was an interaction between sex and age in the Sargent jump test (F = 95; p < 0.001; Fig 1). This was due to a larger difference between age groups in boys compared to girls. For example, the effect size of the difference between 13 and 15 years was 0.99 (95% CI 0.89, 1.08) for boys compared to 0.23 (95% CI 0.16, 0.30) for girls (Fig 2; Table 2).

### Standing long jump

There was an interaction between sex and age in the standing long jump test (F = 132; p < 0.001; Fig 1). This was due to a larger difference between the age groups for boys compared to girls. For example, the effect size of the difference between 13 and 15 years was 1.15 (95% CI 1.05, 1.25) for boys compared to 0.26 (95% CI 0.20, 0.33) for girls (Fig 2; Table 2).

### 30m sprint

There was an interaction between sex and age for the 30 m sprint test (F = 83; p < 0.001; Fig 1). This was due to a larger difference between age groups for boys compared to girls. For example, the effect size of the difference between 13 and 15 years was 0.74 (95% CI 0.64, 0.84) for boys compared to 0.02 (95% CI −0.05, 0.09) for girls (Fig 2; Table 2).

### Medicine ball chest throw

There was an interaction between sex and age in medicine ball throwing (F = 226; p < 0.001; Fig 1). This was due to a larger difference between age groups for boys compared to girls. For example, the effect size of the difference between 13 and 15 years was 1.51 (95% CI 1.41, 1.61) for boys compared to 0.40 (95% CI 0.33, 0.47) for girls (Fig 2; Table 2).

### 6 min shuttle run

There was an interaction between sex and age on the shuttle run test (F = 40; p < 0.001; Fig 1). This was due to a larger difference between the age groups for boys compared to girls. For example, the effect size of the difference between 13 and 15 years was 0.48 (95% CI 0.38, 0.58) for boys compared to 0.07 (95% CI 0.003, 0.14) for girls (Fig 2; Table 2).

### Correlation analysis

**Fig 3:**
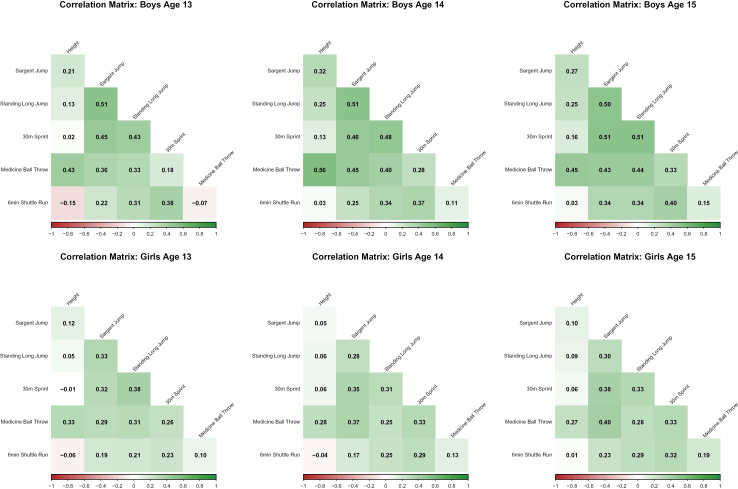
Correlation between all factors for all ages in boys and girls.

Pearson correlation analyses were conducted to examine relationships between height and fitness test outcomes, as well as inter-test correlations, for boys and girls aged 13, 14, and 15 years (only for the 8,486 participants with complete data for matrix generation; Fig 3; S1 File). Height showed moderate to weak correlations with most fitness outcomes, with notable sex differences (Fig 3). For boys, the strongest correlations were observed between height and Medicine Ball Throw, particularly at age 14 (r = 0.56), followed by ages 15 (r = 0.45) and 13 (r = 0.43). These correlations were consistently stronger in boys than girls (P < 0.01; S2 File), suggesting a stronger association between height and upper-body strength in males.

Overall, boys showed stronger correlations than girls (P < 0.01; S2 File) for all variable pairs except medicine ball throw vs. shuttle run (S2 File). The largest correlations across both sexes were observed for Sargent Jump and Standing Long Jump with 30m Sprint, peaking at age 15. Weak correlations between 6 min Shuttle Run and other measures (Fig 3; S2 File, S3 File) suggest that aerobic endurance is relatively independent of strength and power in this population.

## Discussion

In the current study, we examined sex differences in physical fitness parameters among non-athletic adolescents aged 13–15 years, revealing significant sex-by-age interactions across all measured variables. Boys demonstrated considerably larger differences across the age spectrum compared to girls, with notably higher effect sizes for height, Sargent jump, standing long jump, 30m sprint, medicine ball throw, and 6-minute shuttle run. The stronger correlations between height and power-based fitness metrics in boys compared with girls further underscores the sex-specific developmental trajectories during this critical adolescent period.

The performance advantages demonstrated by boys in tests of strength, power, and speed correspond to the well-documented physiological changes occurring during puberty. The surge in testosterone experienced by adolescent males promotes increased muscle mass, bone density, and cardiovascular capacity that directly enhance performance in tests requiring explosive strength and power [1,3]. Girls’ earlier maturation and different body composition changes – characterized by increased fat mass accumulation – do not confer the same performance advantages and may partially explain the small or non-existent age differences in several fitness parameters [4]. Pre-pubertal differences in muscle mass widen substantially during puberty, which explains our findings of markedly larger effect sizes for boys across all fitness tests. These biological differences manifest most prominently in power-based tests but also extend to speed and endurance measures, consistent with previous large-scale studies on adolescents that established sex- and age-specific reference values [9,18].

A particularly significant finding from our investigation is the inference of a “plateau effect” observed in girls’ physical fitness parameters (lack of physiologically relevant performance differences across age groups). This potential plateau is consistent with previous findings indicating that fitness improvements are moderate in female adolescents during this period [6,32,33]. The implications of this may extend beyond physical performance metrics to psychological aspects of sports participation. It may undermine girls’ perceived competence and confidence in physical activities [16], potentially contributing to the documented higher dropout rates among adolescent girls compared to boys [13]. Coupled with minimal improvement in endurance capacity (e.g., 6-minute shuttle run; effect size of 0.07), this may place adolescent girls at elevated risk for inactivity-related health complications, particularly given that adolescent girls are already less active than boys [34].

The findings of this study have substantive implications for developing targeted interventions to address sex-specific performance trajectories. Resistance training programs represent a particularly promising approach for adolescent girls, offering both performance enhancement and injury prevention benefits [35]. Research indicates that properly structured resistance training can reduce sports-related injuries in adolescents, with particular effectiveness in preventing ACL injuries in girls [36]. Such training approaches not only improve physical fitness parameters but also enhance physical self-perception, potentially mitigating the decline in perceived competence that contributes to sport dropout. Furthermore, integrating resistance training with diverse movement activities in school-based programs can promote physical literacy development [37,38]. The timing of such interventions is important. Ideally, they should begin when girls show sufficient emotional maturity to follow instructions and, for athletic girls, be intensified before the sports season to build basic fitness.

This study establishes comprehensive reference values for physical fitness in non-athletic adolescents aged 13–15 years while highlighting significant sex differences in fitness during this critical period. The large sample size of nearly 10,000 non-athletic adolescents provides robust, generalizable data that avoids the selection bias inherent in athlete-focused studies and can be used for reference comparisons. In addition, the comprehensive battery of fitness tests offers a multidimensional evaluation of physical capabilities, and the focus on ages 13–15 captures a critical window of development [39]. The pronounced divergence in fitness between boys and girls – characterized by considerably larger fitness differences across age in boys versus girls – likely reflects the differential impact of pubertal development on physical capacities. These differences may have important implications for both health and sport participation and may contribute to the higher rates of physical activity/sport dropout among adolescent girls. Cultural and social factors in Iran that may limit girls’ participation in physical activity relative to boys might be another contributing factor. However, the cross-sectional design precludes definitive causal inferences regarding developmental trajectories, highlighting the need for longitudinal investigations to track individual development patterns over time. The findings are also relevant in relation to previous work showing that physical fitness in adolescence is associated with long-term health outcomes [40–42] and sports participation [11,15,43]. Yet uncontrolled variables such as socioeconomic status, nutritional factors, and engagement in recreational physical activities may influence the observed results [44,45]. The exclusion of a greater proportion of boys due to regular sports participation may result in a more selected male subsample, though this also ensures the remaining sample better represents non-athletic adolescents. Future research should address these limitations and extend the assessment to other aspects of physical fitness such as body weight, flexibility, motor control and balance.

### Summary and practical implications

- Boys showed significantly greater fitness differences than girls across the age spectrum. Significant plateau effects were inferred from girls’ physical fitness data.
- The positive correlations between height and strength/power-based fitness metrics were considerably stronger in boys than in girls.
- By understanding these sex-specific patterns, educators, coaches, and health professionals can design more effective and targeted interventions to support adolescent physical development, prevent injury, improve performance, and promote lifelong engagement in physical activity and sport.

## Supporting information

S1 File Correlations between factors. (PDF)

S2 File Table of correlation comparision between sexes. (XLSX)

S3 File All data for fitness tests (Sargent jump, Standing long jump, 30m sprint, Medicine ball chest throw, and 6-minute shuttle run records). (PDF)

S4 File Inclusivity in global research. (PDF)
