# Supplementary material for: Sex differences in physical fitness among 10,000 adolescents aged 13–15 years
Source: PLoS One. 2026 Mar 20;21(3):e0345291. doi: 10.1371/journal.pone.0345291 (PMC13004366; doi:10.1371/journal.pone.0345291)

Supplementary file 1: Correlations between factors

Sex Differences in Physical Fitness Among 10,000 Adolescents Aged 13–15 Years

Authors

Ali Gorzi [1,2*], Hamid Rajabi [3], Mina Khantan [4], Tommy R. Lundberg [2,5]

Affiliations

1. Department of Sport Sciences, University of Zanjan, Zanjan, Iran
2. Division of Clinical Physiology, Department of Laboratory Medicine, Karolinska Institutet, Stockholm, Sweden
3. Department of Sport Sciences, Kharazmi University, Tehran, Iran
4. Faculty of Sport Sciences and Health, Shahid Beheshti University, Tehran, Iran
5. Unit of Clinical Physiology, Karolinska University Hospital, Stockholm, Sweden

Correspondence

Ali Gorzi, PhD; Division of Clinical Physiology, Department of Laboratory Medicine, Karolinska Institutet, Stockholm, Sweden

Email: ali.gorzi.2@ki.se

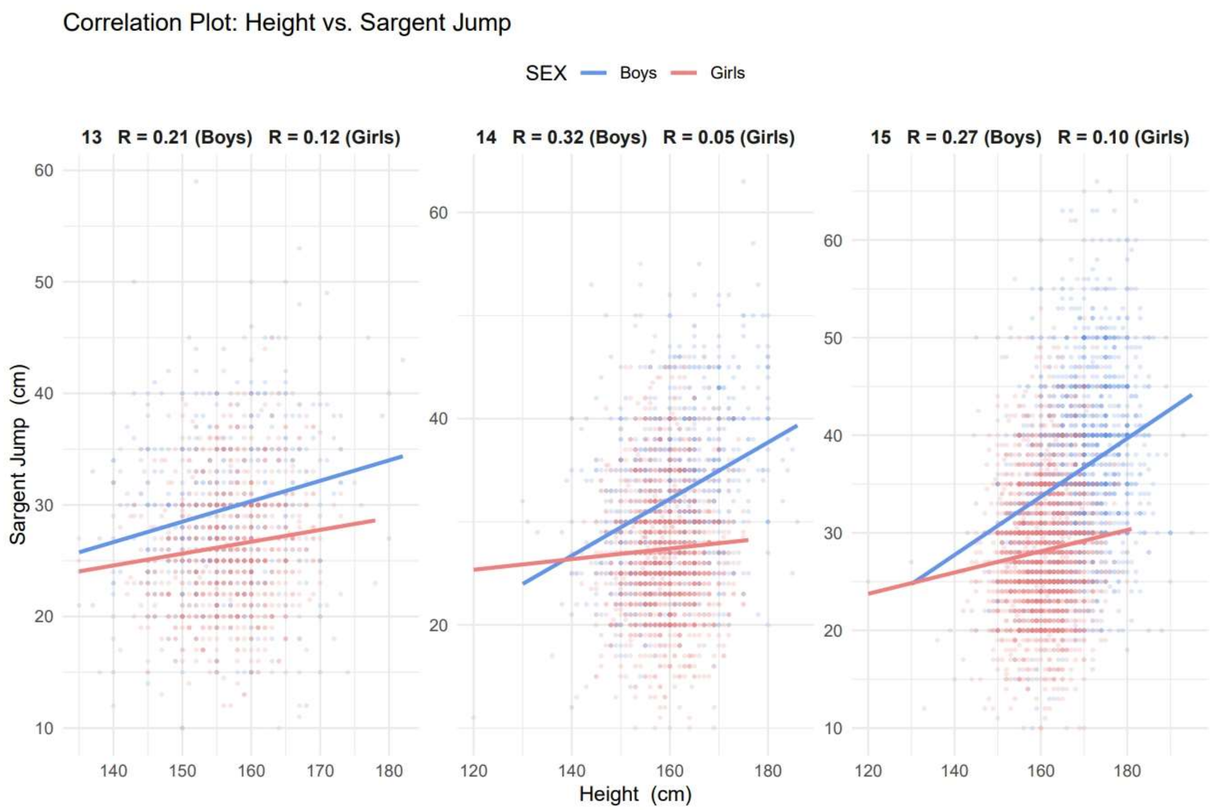

Correlation Plot: Height vs. Standing Long Jump

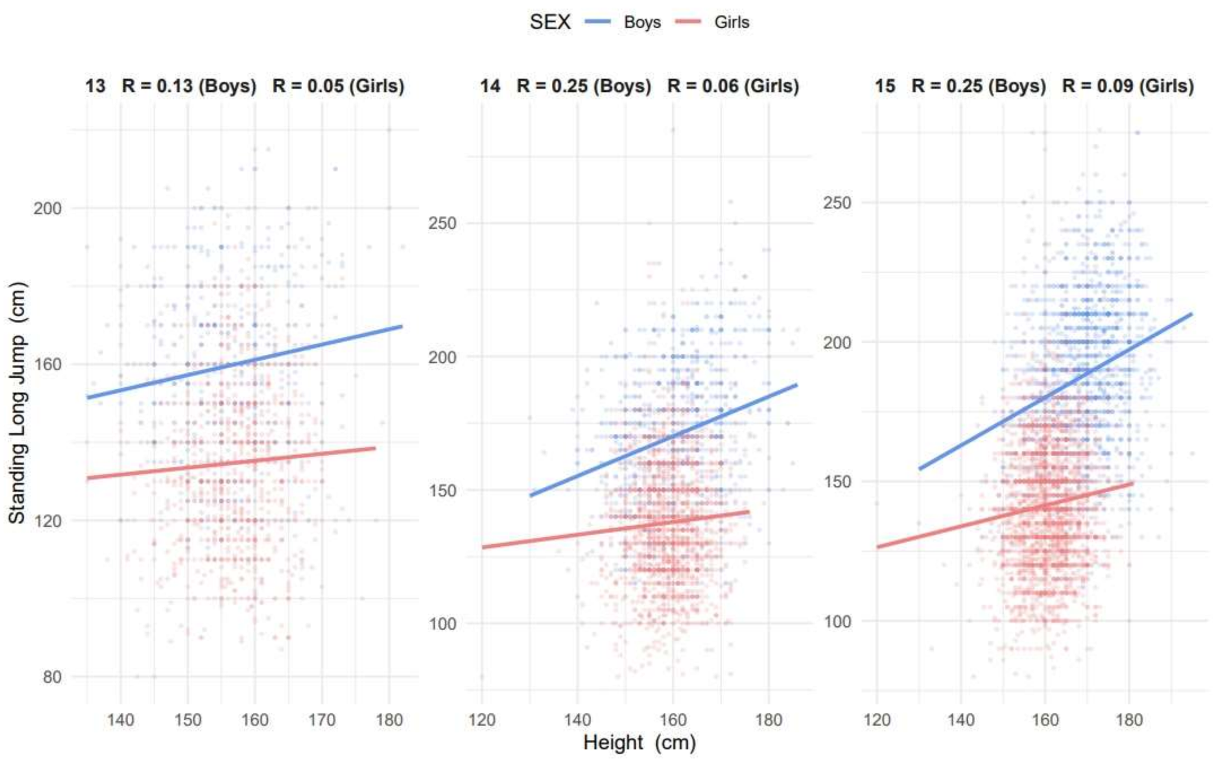

Correlation Plot: Height vs. 30m Sprint Performance

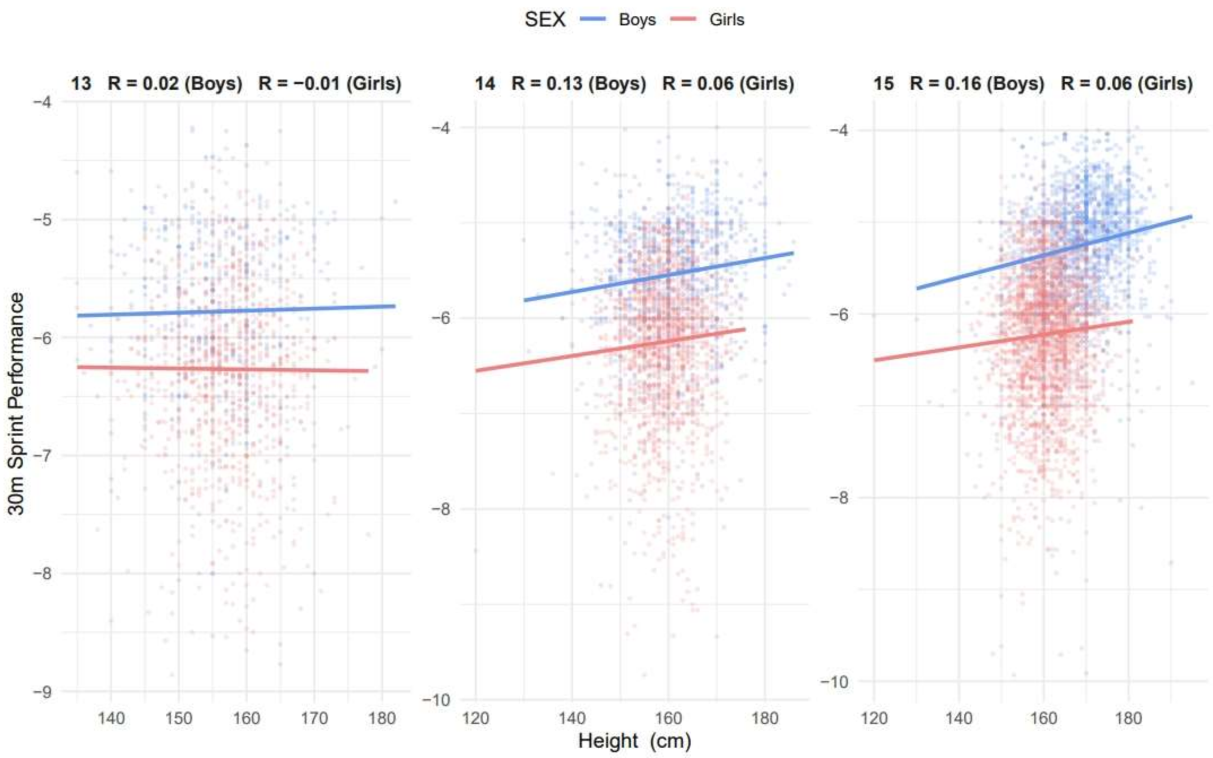

Correlation Plot: Height vs. Medicine Ball Throw

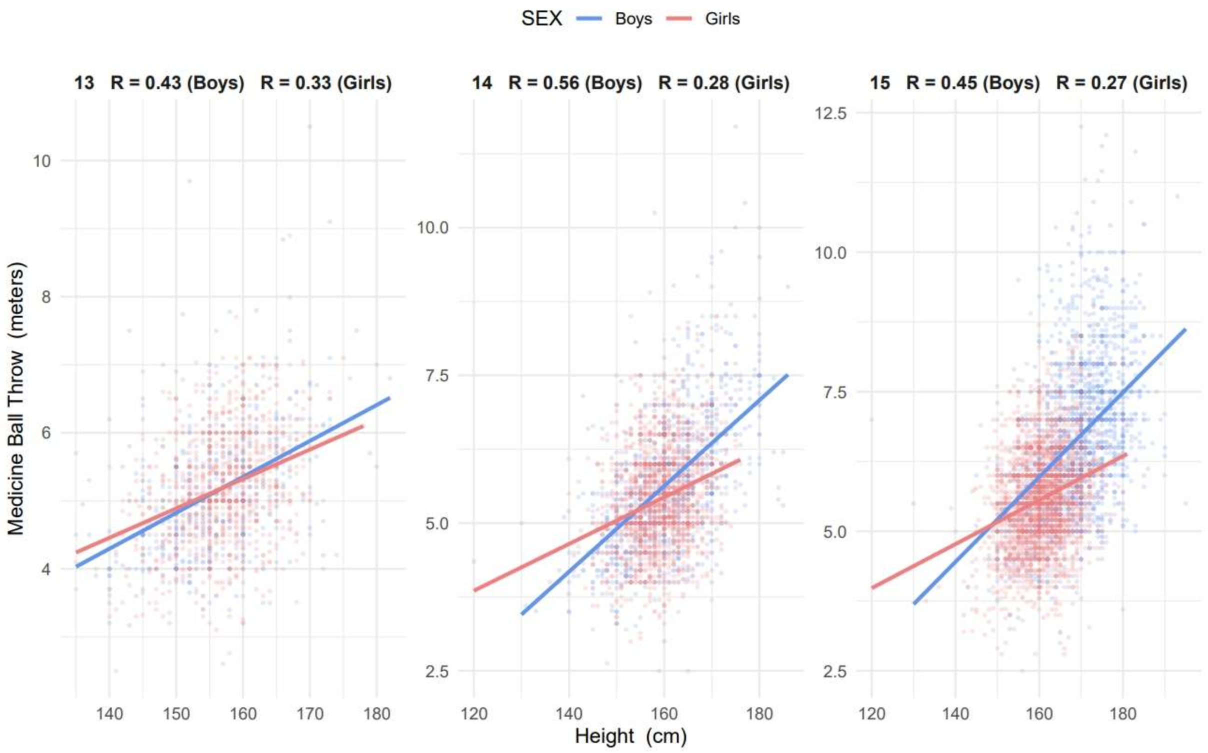

Correlation Plot: Height vs. 6min Shuttle Run

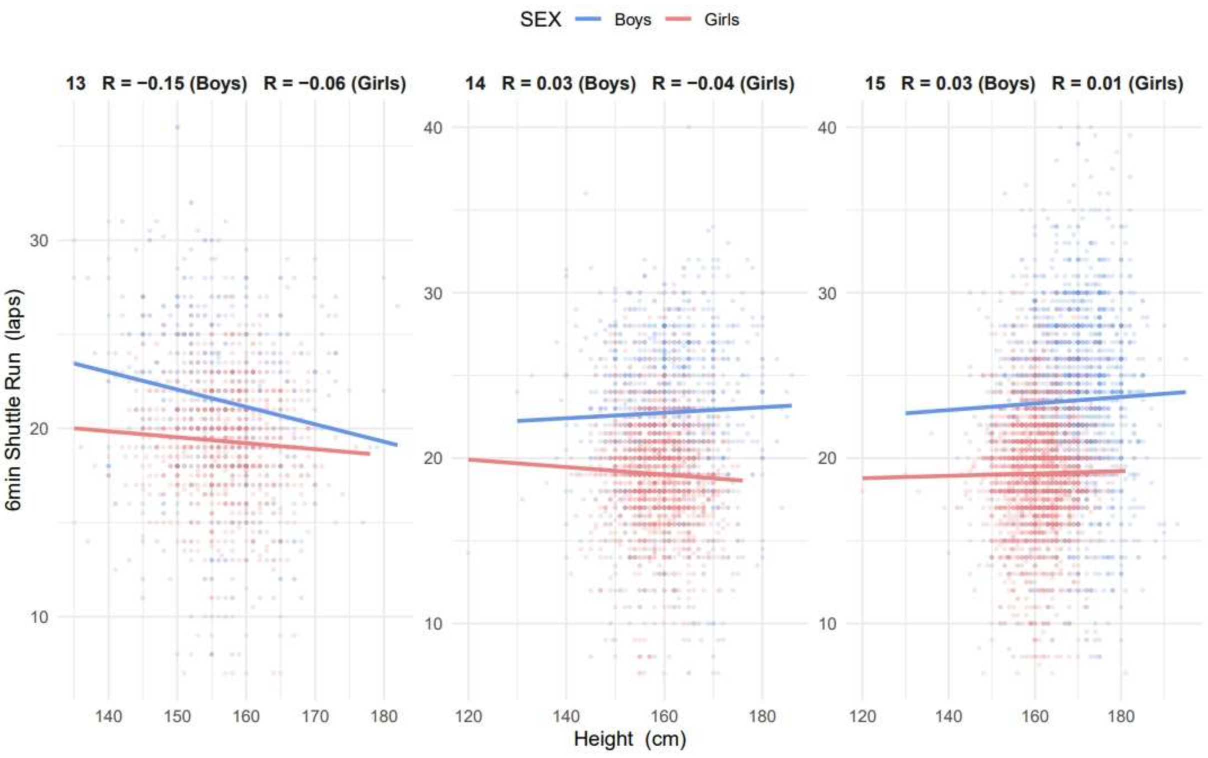

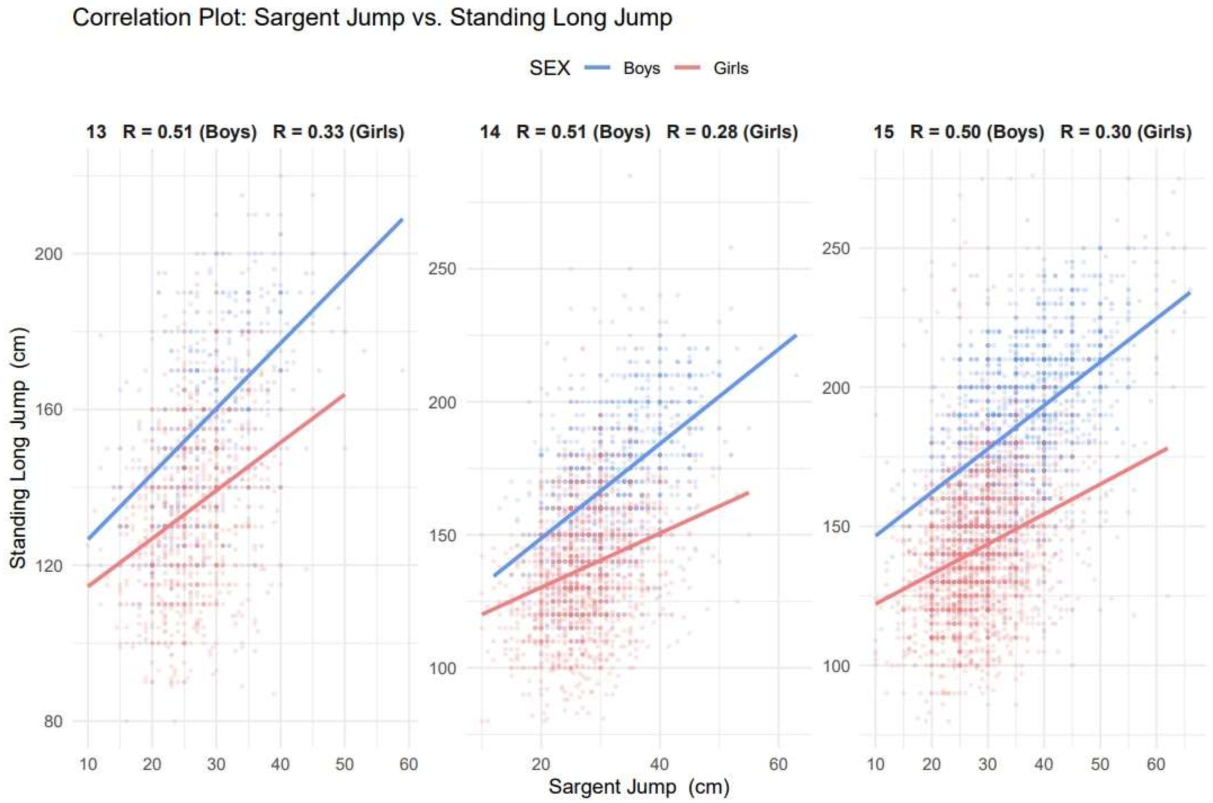
Correlation Plot: Sargent Jump vs. Standing Long Jump
SEX Boys Girls
13 R = 0.51 (Boys) R = 0.33 (Girls)
14 R = 0.51 (Boys) R = 0.28 (Girls)
15 R = 0.50 (Boys) R = 0.30 (Girls)
Standing Long Jump (cm)
Sargent Jump (cm)

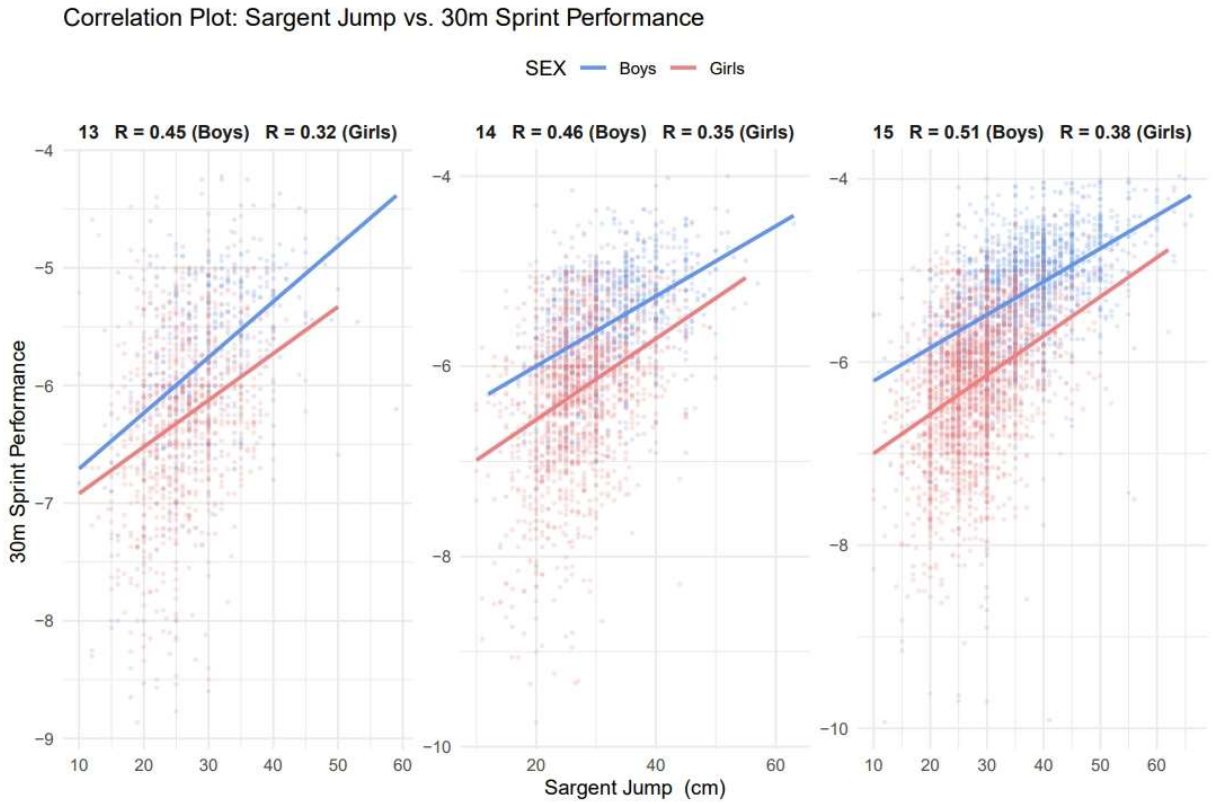
Correlation Plot: Sargent Jump vs. 30m Sprint Performance
SEX Boys Girls
13 R = 0.45 (Boys) R = 0.32 (Girls)
14 R = 0.46 (Boys) R = 0.35 (Girls)
15 R = 0.51 (Boys) R = 0.38 (Girls)
30m Sprint Performance
Sargent Jump (cm)

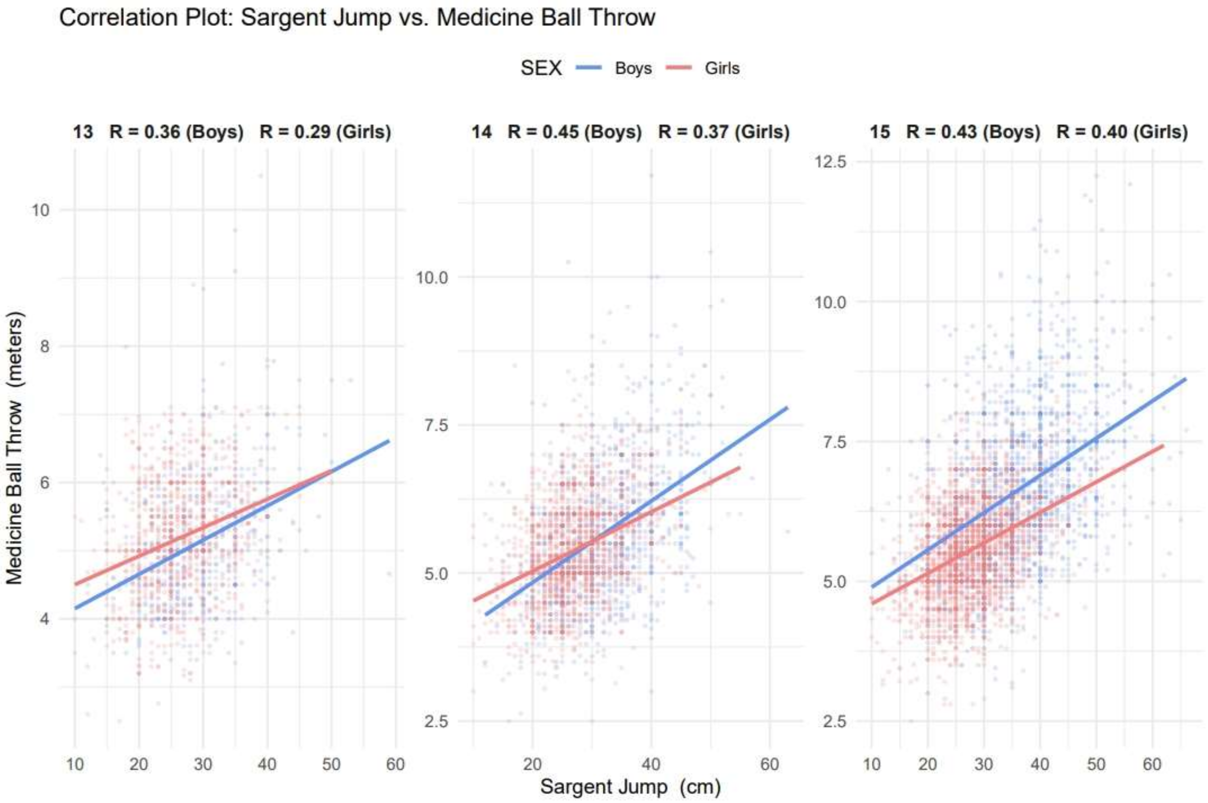
Correlation Plot: Sargent Jump vs. Medicine Ball Throw
SEX Boys Girls
13 R = 0.36 (Boys) R = 0.29 (Girls)
14 R = 0.45 (Boys) R = 0.37 (Girls)
15 R = 0.43 (Boys) R = 0.40 (Girls)
Medicine Ball Throw (meters)
Sargent Jump (cm)

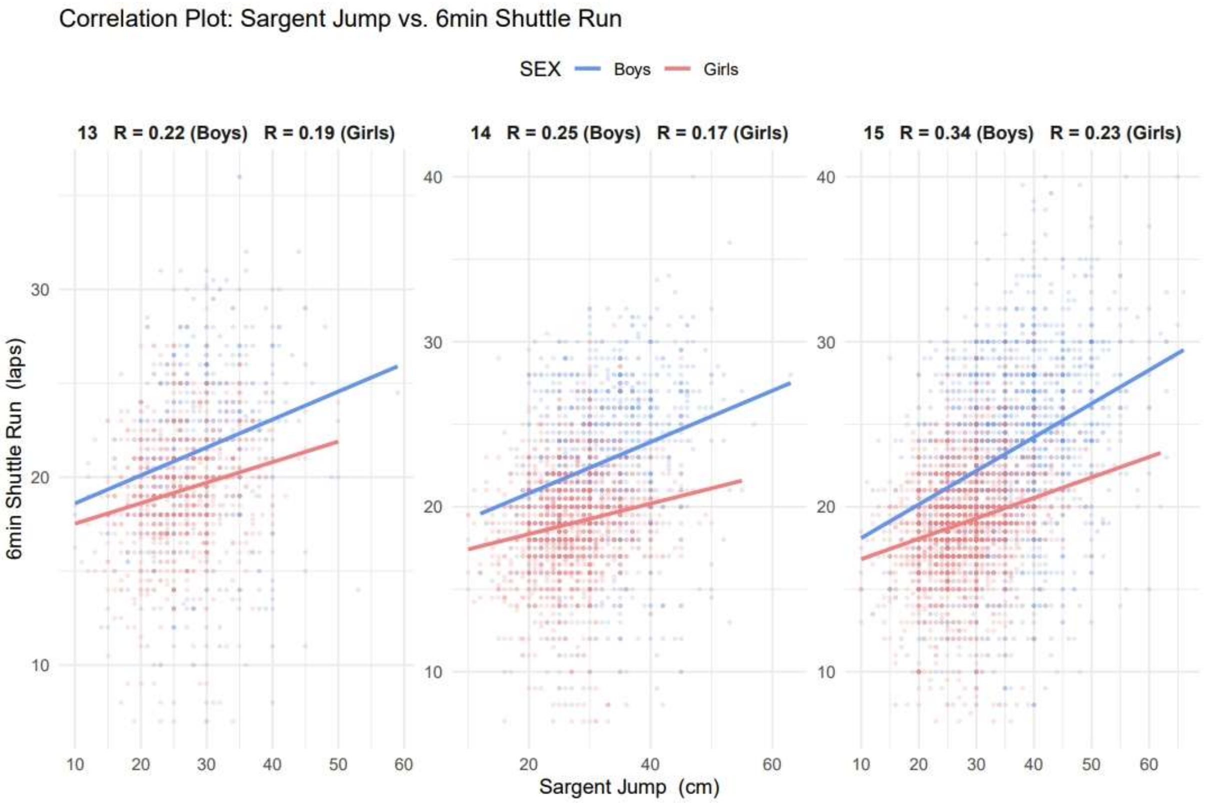
Correlation Plot: Sargent Jump vs. 6min Shuttle Run
SEX Boys Girls
13 R = 0.22 (Boys) R = 0.19 (Girls)
14 R = 0.25 (Boys) R = 0.17 (Girls)
15 R = 0.34 (Boys) R = 0.23 (Girls)
6min Shuttle Run (laps)
Sargent Jump (cm)

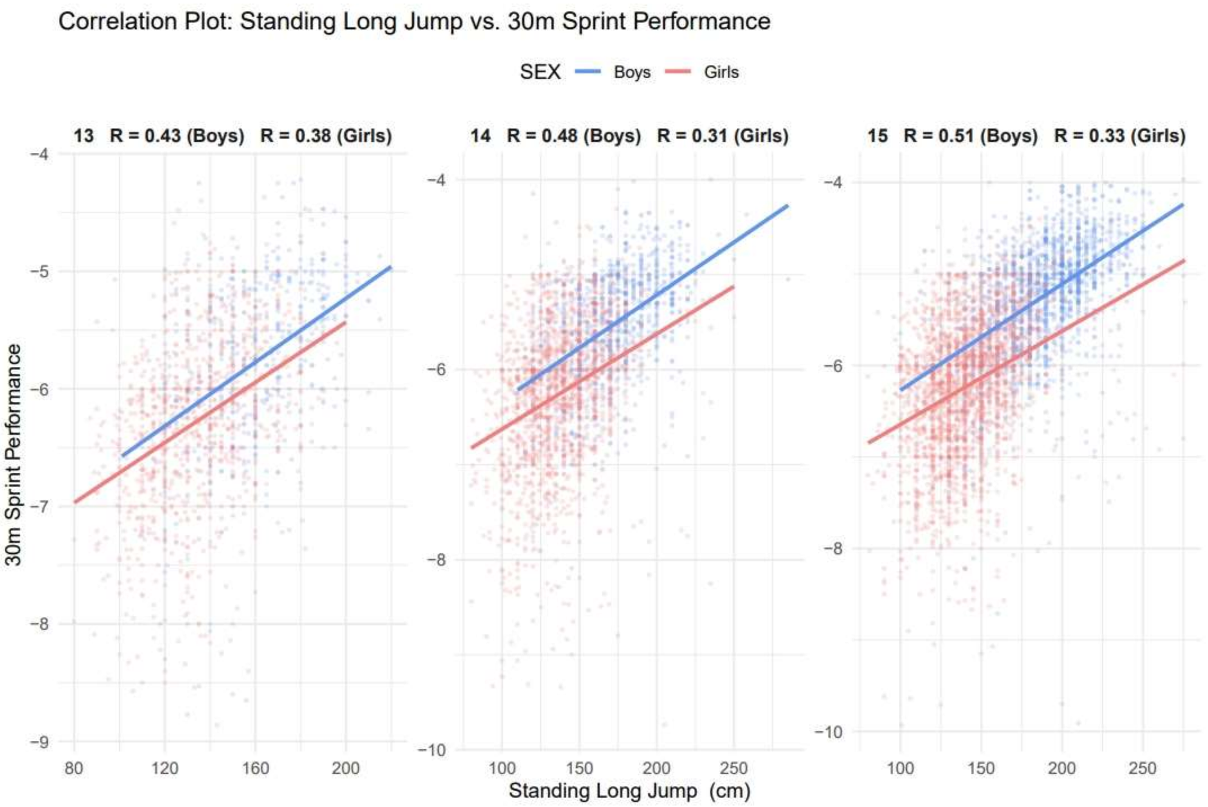
Correlation Plot: Standing Long Jump vs. 30m Sprint Performance
SEX Boys Girls
13 R = 0.43 (Boys) R = 0.38 (Girls)
14 R = 0.48 (Boys) R = 0.31 (Girls)
15 R = 0.51 (Boys) R = 0.33 (Girls)
30m Sprint Performance
Standing Long Jump (cm)

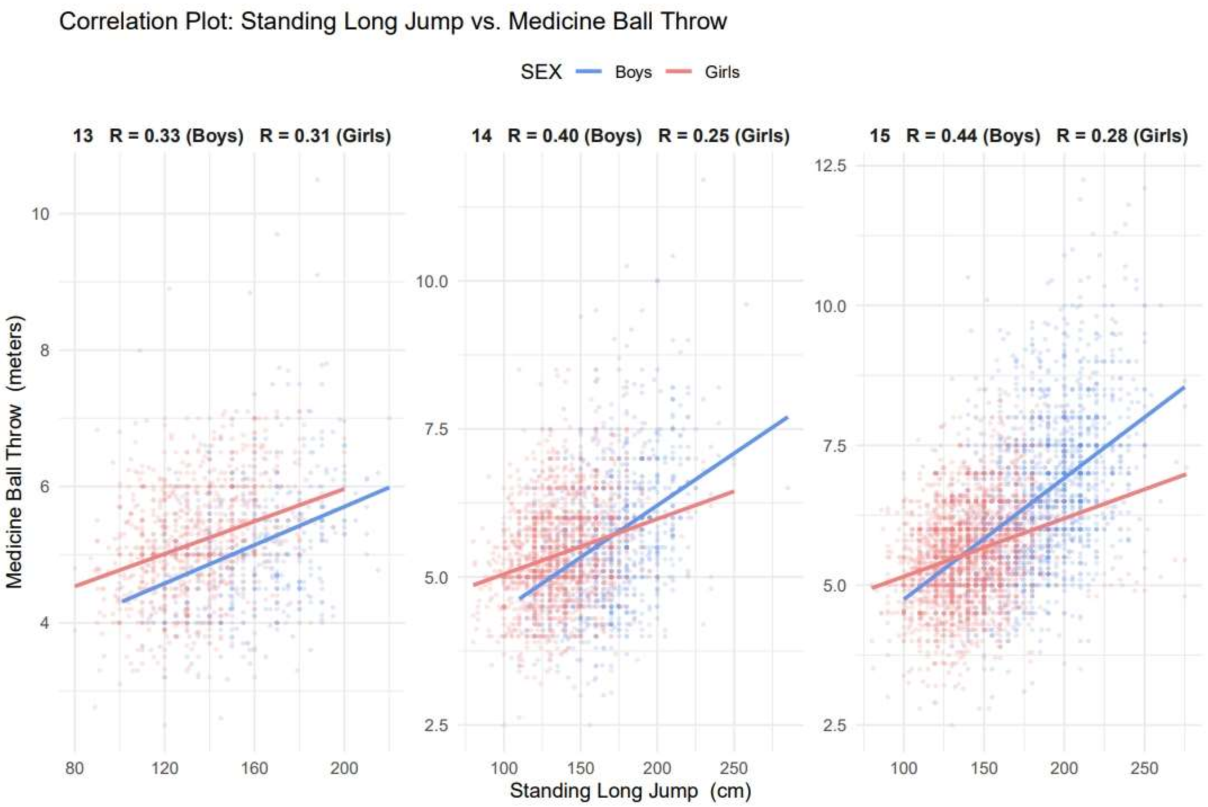
Correlation Plot: Standing Long Jump vs. Medicine Ball Throw
SEX Boys Girls
13 R = 0.33 (Boys) R = 0.31 (Girls)
14 R = 0.40 (Boys) R = 0.25 (Girls)
15 R = 0.44 (Boys) R = 0.28 (Girls)
Medicine Ball Throw (meters)
Standing Long Jump (cm)

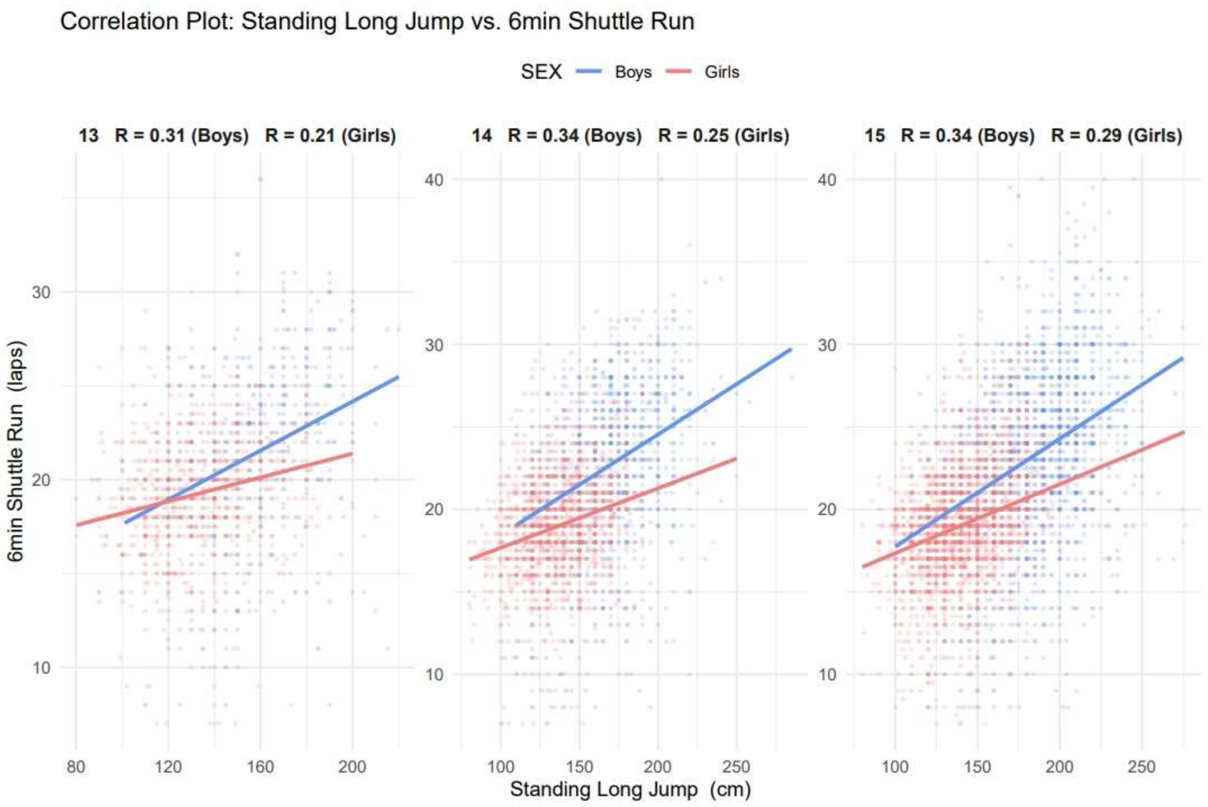
Correlation Plot: Standing Long Jump vs. 6min Shuttle Run
SEX Boys Girls
13 R = 0.31 (Boys) R = 0.21 (Girls)
14 R = 0.34 (Boys) R = 0.25 (Girls)
15 R = 0.34 (Boys) R = 0.29 (Girls)
6min Shuttle Run (laps)
Standing Long Jump (cm)

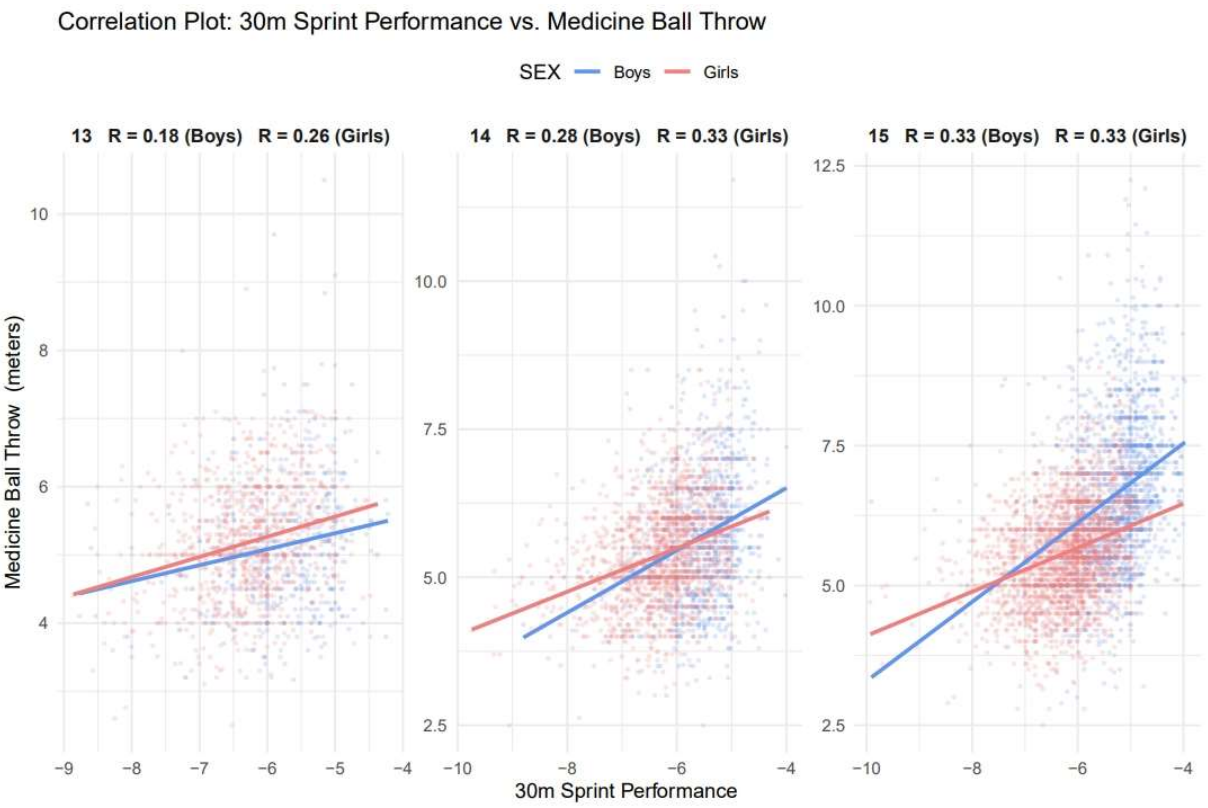
Correlation Plot: 30m Sprint Performance vs. Medicine Ball Throw
SEX Boys Girls
13 R = 0.18 (Boys) R = 0.26 (Girls)
14 R = 0.28 (Boys) R = 0.33 (Girls)
15 R = 0.33 (Boys) R = 0.33 (Girls)
Medicine Ball Throw (meters)
30m Sprint Performance

Correlation Plot: 30m Sprint Performance vs. 6min Shuttle Run

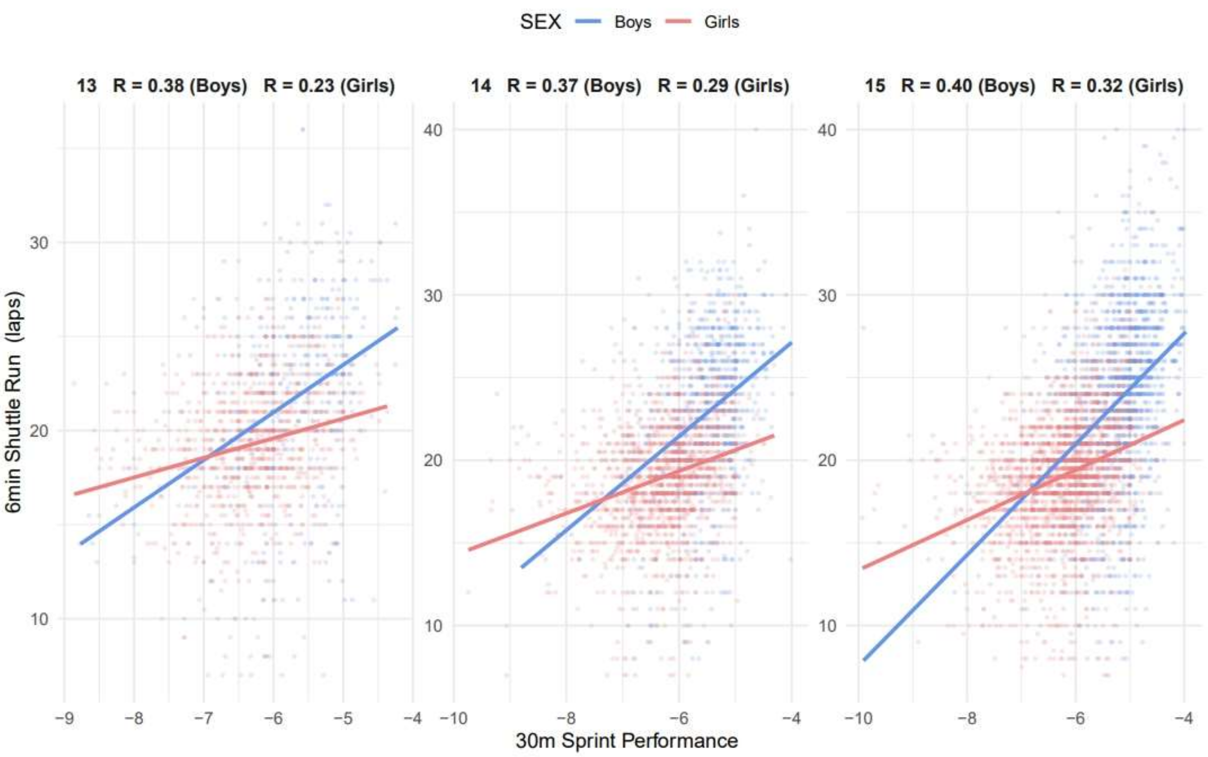

Correlation Plot: Medicine Ball Throw vs. 6min Shuttle Run

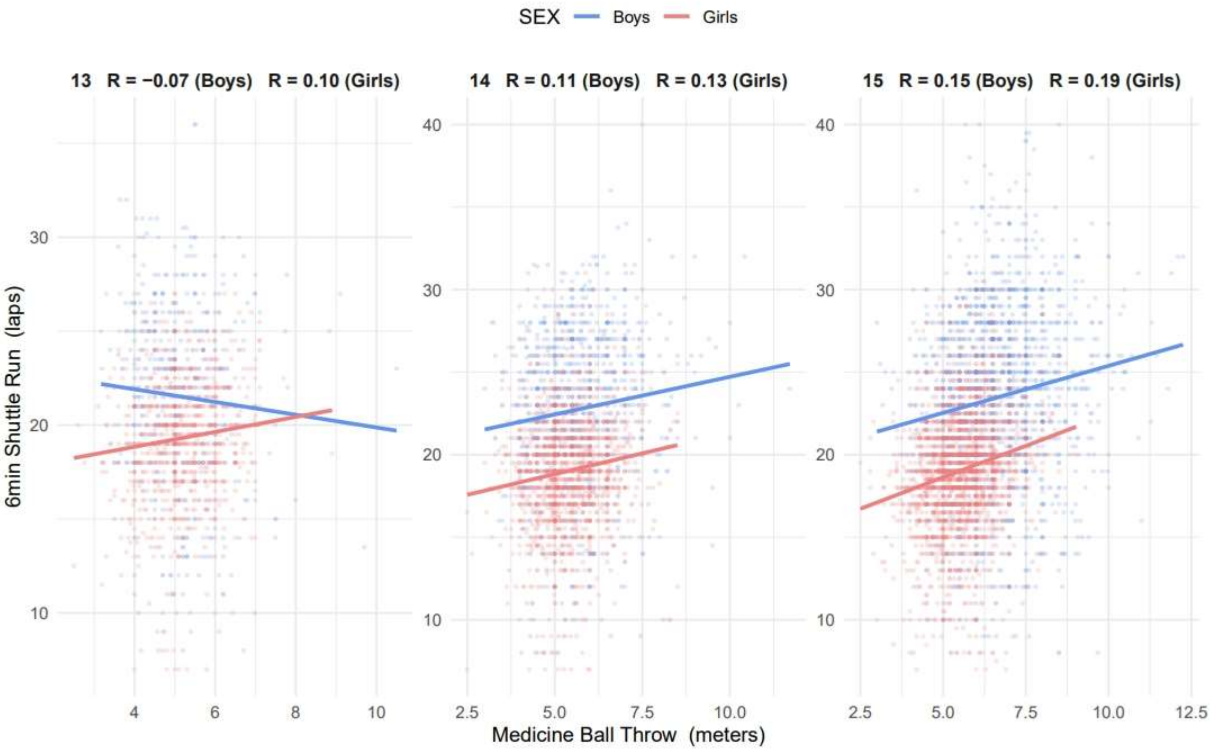

Supplement: S1 File — (PDF) [file pone.0345291.s001.pdf]
