# Supplementary material for: Sex differences in physical fitness among 10,000 adolescents aged 13–15 years
Source: PLoS One. 2026 Mar 20;21(3):e0345291. doi: 10.1371/journal.pone.0345291 (PMC13004366; doi:10.1371/journal.pone.0345291)
Supplement: S3 File — (PDF) [file pone.0345291.s003.pdf]

Height	Sargent J	Stand. L.J	30m	Med-2kg	6min.shuttle.Run	SEX	Age
	31	172	5.51	4.7	21.75		
	16	135	6.59	4.6	17.5		
	37	189	5.24	6	25		
	28	167	5.65	5.5	22.5		
	30	180	5.86	5	19.25		
	28	157	6.38	5	17.25		
	27	180	6.31	4.6	20		
	26	170	5.84	6	24		
	23	150	6.04	4.76	23		
	24	145	7.22	4.7	17		
	27	166	6.05	5.3	27		
	29	196	6	6.25	18		
	32	189	6	4.8	25		
	26	176	6.6	5.92	18		
	31	134	6	4	18		
	26	155	6.3	4.95	19		
	26	156	6.22	6.12	23		
	23	175	6.12	4.55	31		
	27	175	6	4.26	23		
	25	155	7	4.15	26.5		
	31	190	6.5	4.12	25		
	16	130	8	3.5	15		
	29	174	6.15	5	23		
	18	156	8.65	5.75	15.5		
	24	166	6.18	4.9	26		
	29	195	6.5	6.1	19		
	27	172	6.07	5.2	28		
	38	190	5.55	5.3	21		
	39	167	5.84	5	20		
	23	180	5.8	4.9	22.25		
	32	150	6.2	4.3	22.5		
	28	170	6.71	5.5	19.5		
	26	156	6.7	5	26		
	22	160	5.76	4.7	15.5		
	19	140	7.4	3.8			
		172	5.56	5.7	20		
	35	160	6.49	4.5	18		
	34	170	5.04	6	21		
	34	165	6.03	3.5	18		
	23	155	6.01	5	20.5		
	25	170	5.03	4.5	21.5		
	33	190	5.03	6.6	20.5		
	23	140	7	4.5			
	29	185	5.03	4.7	23		
	22	150	5.07	4.5	19.5		
	24	155	6	4	20		
	33	168	4.83	5.2	22		
	23	140	6	4	21		
	27	170	5.03	4.5	20		
	36	200	4.86	6.2	22		
	16	167	5.04	4.2	20.5		
	34	168	5.02	4.7	24		

18	155	5.08	4.5	15.5
18	145	6.06	3.5	18
22	150	5.77	6	16
24	140	6	4	19
35	180	5.57	4.5	27
35	190	4.47	5.5	30
35	160	5.58	5.5	36
40	190	5.23	5.5	27
25	145	5.23	4	26.5
23	120	6.88	4.18	19.5
33	168	6	4.66	23.5
38	162	5.25	5.7	24
49	186	5.44	6.7	20
39	192	5.75	6.25	22
46	170	5.44	5	25
33	151	5.38	5	23
36	145	5.75	4	23.25
40	200	4.96	5.7	24
59	170	6.2	4.66	24.5
25	160	6.39	5.05	20
25	150	6.52	4.9	
20	150	6.5	4.56	24
22	150	6.2	4.36	15
24	150	6.12	3.9	25
38	113	5.76	4	22.5
27	148	5.61	3.17	25
28	135	5.94	3.2	23
35	154	6.5	5.07	19
35	132	5.87	4.82	20
26	140	6	4.35	25
19	101	6.2	4.52	16
32	157	5.15	3.7	23
20	118	8	3.65	18.5
30	150	4.94	4.22	30
36	150	5.2	3.65	32
30	150	5.3	3.78	26.5
44	150	5.25	3.8	32
33	140	6.25	5.35	22
26	122	6.93	5.05	18.5
34	180	5.75	4.96	26.5
30	150	5.86	4.26	25.5
34	185	5.8	4.38	23.5
30	150	6.32	5	19.5
22	170	5.08	3.15	
10	120	6.83	4	18
27	155	5	4.9	23
32	177	4.25	3.8	23.5
22	125	5.15	4.3	20
30	150	5.06	4.2	21.5
24	138	4.72	4.2	25.5
25	125	5.2	4.1	21.5
36	170	4.25	4.2	31
34	165	4.97	5.7	22

45	182	5	6.7	29
40	140	5.32	4.1	21
26	110	6.05	4.1	20
30	135	4.95	4.2	26
25	145	6.7	5	19
22	135	5.9	5.1	20.5
32	185	4.9	5.1	23
28	150	5.97	4.8	20
24	125	7.15	5.1	17
21	140	5.3	5.1	21.5
23	135	6.1	4.8	19
35	160	5.37	6.7	24
29	168	5.8	5.8	21.5
30	160	5.55	5.8	22.5
27	158	5.4	3.6	20
30	150	6.5	4.8	18.5
20	110	6.2	3.6	18
23	142	5.44	4.6	20.5
30	130	5.15	5.7	26
25	145	6.22	4.4	19.5
39	200	5.05	6	29
28	149	5.06	4.2	27.5
18	125	6.1	3.8	18
24	131	5.85	3.8	19
20	118	6	4.2	18
29	158	5.74	3.4	19
30	168	5.15	4.8	22
27	125	6.15	5.2	18
18	95	7.15	3.5	
29	135	4.25	5.8	26
23	125	5.95	6	19.5
34	160	6.18	4.8	24
31	185	5.8	3.6	29.5
31	170	5.09	5.2	30.5
26	170	6.05	4.3	19.5
31	135	7.1	4.5	25.5
30	170	6.28	6	24.5
24	152	6.19	4.5	23.5
27	170	6.5	3.9	24
20	155	6.64	3.7	23
30	160	6.37	4.1	23
26	150	5.65	4.4	31
28	200	5.03	5.4	29.5
19	145	6.16	5.1	22
32	190	5.31	5.4	30
30	180	5.81	5.1	26
22	195	5.41	5.4	25.5
30	150	6.5	5.6	21
31	190	5.4	4.6	28
30	130	6.63	4.18	
26	160	5.21	3.6	
23	130	6.54	5.15	23
29	180	5.62		26.5

35	190	5.53	4	23.5
25	132	6.03	3.4	24
36	210	5.03	6.1	26
36	180	5.6	5.74	28
33	190	4.9	5.29	30.5
34	170	6.71	5.4	29
32	200	5.38	6.6	30
30	160	5.89	4.3	
27	195	5.3	4.1	27
30	170	6.01	4.68	26.5
40	180	6.8	4.52	27
21	195	5.91	4.23	28.5
34	215	5.41	4.77	28
26	170	6.01	4.94	23
28	160	6.24	3.6	23.75
33	171	5.9	7.74	25
35	188	5	9.1	27
30	150	6.56	6.98	10
41	190	5	7.78	28
27	192	5.4	6.2	28
22	138	6.5	5.36	18
30	158	5.15	8.84	25
21	140	6.4	5.93	18
27	170	5.06	6.2	30
34	195	6.15	5.65	19
23	133	6.53	5	21
21	110	7	5.56	
31	200	5.38	6.96	21
20	120	6.3	4	20
36	198	5.71	6.98	27
22	118	6.62	5.5	15
40	192	5.2	7.8	22
28	158	6.22	5.9	13
32	178	5	6.35	22
24	138	6.25	6.6	17
31	155	5	5.8	23
28	122	5.81	6.25	20
23	135	7.28	4.7	10
33	172	5.56	6	27
39	188	5.16	10.5	23
32	152	5.7	7.1	24
18	109	7.25	7.99	16
25	145	6	5.3	24
32	125	6.1	4	25
28	130	5.8	4.3	18
31	170	5.45	5	29.5
27	120	5.7	4.7	27
35	120	5.38	4.5	26.5
32	160	5.15	6.2	27.5
24	120	5.22	4	21.5
41	150	5.32	6.2	23
26	115	5.9	4.2	22.5
27	130	5.5	4	24.5

31	125	5.51	5.1	22
34	180	4.7	5.8	26
23	130	6.02	5.9	21
28	140	5.42	6.2	21
35	140	5.64	5.05	20.5
33	150	5.13	4.6	22
24	125	5.7	4.1	23.5
37	125	5.45	6.2	25
28	150	5.6	5	23.5
42	165	4.97	5	24
21	125	6.28	5	20.5
21	125	7.3	4.7	21
32	185	4.94	6.2	24
21	115	6.6	4	22
30	140	5.81	4.5	27
36	120	6.05	5.5	21.5
37	155	5.2	5	23.5
28	130	5.34	4.3	24.5
40	170	4.9	6.2	23
28	130	5.59	4.5	22
30	160	5.3	4.7	27
36	155	5.57	5	25
27	150	5.6	5	25.5
30	150	5.55	5	24
41	170	4.8	7	27
37	160	5	5.5	27
25	160	6.1	4.6	20
15	140	8.06	3.8	18
20	120	7.2	3.2	18
30	192	5.14	5.2	28
22	150	6.75	4.2	21
30	160	5.05	4.5	25
30	162	6.31	4.5	25
33	162	5.37	4.85	23.5
32	160	5.09	4.3	25
35	170	5.69	4.7	21.5
35	165	6.02	4.5	19
38	145	6.25	5.7	19
27	120	5.97	5.35	15
30	165	7	5.2	26
30	170	6.5	4.7	26
30	170	5.9	4.3	30.2
30	180	6.18	4.6	25.5
30	155	6.5	4.7	23
30	190	6	4.3	25.5
30	190	5.3	4.05	31
35	190	5.47	6	26
41	172	4.37	6.5	28
40	170	5.25	4.5	26
34	170	4.59	4	29
48	180	4.97	5.5	28
43	190	4.85	6.8	26.5
33	160	5.53	5.55	24

26	149	6.03	4.4	21
25	148	5.6	4.8	23
35	182	5.47	4.5	18
34	174	5.35	4.58	
38	165	4.92	5.25	22.5
25	157	5.04	5.32	
21	130	6.03	5.1	16
33	162	5.1	5.25	20
33	188	5.11	5.8	25
19	120	7.56	4.6	17
21	145	6.08	5.75	17
22	130	6.37	4.2	
32	179	4.95	5.4	
28	160	5.75	5.6	21.5
28	160	6.56	5.8	19
27	170	5.64	4.75	20.5
27	190	5.51	5.55	
23	126	6.72	5.8	19.5
32	180	4.22	5.6	26.5
27	156	5.88	5.2	20.5
30	175	5.51	4.55	26
34	180	6.5	5.54	22.5
23	129	6.53	4.55	17.5
34	155	5.13	5.86	23
32	180	5.5	5.9	23.5
31	168		4	
31	150	5.47	4.8	23
25	145	5.25	6.74	16
35	180	5.57	4.5	27
40	190	4.47	5.5	30
35	160	5.58	5.5	36
40	190	5.23	5.5	27
25	145	5.23	4	26.5
28	180	5.37	4.2	24
30	165	5.8	6.4	25
25	160		5.1	24
27	165	6.09	6.7	25.5
35	170	5.9	9.7	13.5
30	210	5.25	5.7	18
35	210	5.25	5.3	13
27	189	5.43	4.65	22
30	167	5.07	3.96	20
22	160	6.25	5.8	18.5
30	180	5.65	5.6	24.5
14	140	6.87	6.4	12
21	160	5.72	6.4	19.5
15	130	7.45	5.6	18
21	130	6	4.5	23
20	155	5.35	4.6	23
38	186	5.63	6.5	22.5
15	150	6.42	6.2	17
22	155	5.74	5.6	18
23	175	5.89	5.5	17

24	190	5.6	5.7	18
24	170	6.36	5.7	18
24	135	6.48	5.5	19
24	170	5.92	5.9	20
24	150	7.08	6.1	23
15	140	6.65	5.6	17
30	160	6.05	7.35	18.5
24	150	5.5	4.5	26
26	170	5.47	4.7	27
20	135	6.7	4.5	24
26	190	4.9	4.5	25
27	175	5.05	5.5	25
26	175	7.2	4.5	24
22	145	6	3.9	19
15	130	6.9	4	12
22	130	4.9	4.1	22
31	155	5.8	4.37	25
35	174	5.17	5.01	26.5
27	141	6.5	4.78	20
24	137	6.81	4	17.5
37	191	5.23	5.7	23.5
32	142	5.4	4.5	20
27	113	7.23	4.06	18
35	182	5.23	5.32	23.5
39	171	5.03	5.15	24.5
25	152	4.8	3.91	26.5
27	153	6.31	5.03	26.5
27	147	5.9	5.58	22.5
31	157	6.62	6.11	20
16	130	6.7	4.3	22
23	165	5.8	5.3	25
15	120	7.3	3.9	18
26	142	6	5	22
45	184	5.47	7.11	26
27	185	5	4.3	23
24	140	6.5	6.5	18
23	160	5.5	4.2	25
25	125	6.3	4.5	23
26	160	5.6	5.2	22
28	175	5.53	5	21
25	190	6.1	4.3	20
20	130	5.59	4.2	21
26	180	5.35	4.3	26
21	190	4.9	5.7	25
30	200	4.9	6.1	27
23	155	5.75	4	26
23	170	5	4	23
26	180	4.9	4.5	25
23	170	5.34	4	25
20	180	5.1	5	23
21	130	5.25	4.2	21
25	180	5.3	4.6	21
27	165	5.15	5.5	25

25	160	5.9	5.1	
22	145	6.4	5.05	10
27	150	5.7	3.5	
25	150	7	5.2	13
38	185	5.25	5.5	15
24	180	5.3	4.2	24
26	130	6.2	4.8	28
21	190	4.6	5.7	28
30	200	4.54	5.3	28
27	170	5.3	5.5	28
21	150	5.9	4.5	28
26	175	4.82	4.1	28
37	182	4.47	5.06	28
41	182	5.76	4.6	30
34	160	6.4	4.1	18
25	185	5.96	4.9	22
22	184	5.5	6.4	
22	180	5.55	4	14
42	181	5.1	5.6	25
33	184	5.1	5.7	24
33	170	5.31	5.3	18
35	185	5.09	5.2	23
24	135	5.56	3.8	25
38	173	6.44	4.5	15
12	120	8.3	4.5	19
39	196	4.94	5.65	25
15	165	6.9	5.5	18
50	200	5.18	6.3	23
42	150	5.16	5	
45	220	5.25	6	
28	120	5.9	5.3	13
27	140	6.15	5.7	13
39	150	5.7	4.7	13
20	145	6.7	4.1	
20	150	6.09	5.2	
27	200	6.1	5.5	22
30	135	6.25	4.5	
50	180	4.75	7.5	20
35	155	5.16	6.2	20
32	170	5.47	4.7	18.5
35	145	4.89	4.1	21.5
30	140	5.22	5	25
30	150	6.06	4.9	12
25	140	5.16	5.2	16
35	145	5.46	5.8	14
25	150	5.65	6.2	11
40	150	5.72	5.8	13
30	145	5.7	4.6	8
38	165	5.38	5.6	16
32	160	6.56	5.7	21
35	140	6.03	5.5	
30	145	6.72	7.5	
30	168	5.75	7.5	15

38	140	6.7	5.3	13
28	140	6.8	4.1	10
25	145	6.7	4.7	
25	135	6	4.7	12
25	165	6.7	4.2	17
32	125	5.01	5.6	12
30	145	5.61	4.4	16
40	205	5.02	5.5	17
30	175	5.06	7	16
35	185	6.45	6.5	20
25	145	5.89	5.1	
32	160	5.89	6.1	
25	140	4.89	4.9	23.5
38	160	5.16	4.7	23.5
25		6.08	4.2	19.5
30	140	5.62	5.5	21
25	165	5.5	4.9	21.5
37	200	4.75	5.5	22.5
45	215	4.87	6.2	11
30	135	6.14	5.2	11
38	140	5.8	5.6	23
32	145	5.16	4.8	18
40	140	5.3	7	17
35	175	5.96	7	
27		6.6	6.4	15
40	170	5.5	4.9	24
35	160	5.25	5.4	23
42	175	5.09	5	23
30	165	4.87	5.1	21
53	175	5.46	7.5	14
30	140	5.35	6.5	17
21	120	4.98	5	11
35	140	5.12	5.9	18
25	140	5.2	5.3	12
35	140	5.9	5.2	18
40	190	6.12	6.7	8
25	125	6.06	5.2	8
35	200	5.11	6.6	14
45	195	5.38	6.8	16
28	180	6.12	5.2	
15	115			
18	120	5.95	5.2	14
37	165	6.09	5.25	16
30	175	6.25	5.8	
28	155	6.3	6.2	13
40	190	5.2	5.21	13.5
25	145	6.7	4.7	17
35	190	5.12	5.2	20
24	150	6.5	5	16
25	160	6.1	5.29	14
27	155	6.14	5.2	13.5
40	185	5.98	4.8	11
33	163	6.1	5.3	14

45	190	5.41	7.5	15
27	140	5.43	4.22	16.5
26	130	5.37	5.1	18.5
44	195	4.49	5.4	23
35	170	4.88	4.6	23.5
33	160	5.8	4.5	19
35	160	6.25	5.6	23
40	160	6.5	6.7	24.5
20	140	6.69	5.4	
35	160	5.75	5	23.5
25	140	8.03	5.8	
40	170	5.5	6.5	25.5
25	140	6.12	4.5	
40	185	5.6	6	14.5
40	185	5.53	7.1	20
45	200	4.75	5.3	29
27	155	6.61	4.5	16
40	220	4.99	7	28
38	210	6.03	6.12	23
40	210	6.34	5	20
32	140	5.9	5.2	14
30	165	5.12	6.2	14
32	145	6.15	4	11
40	205	5.09	5.9	14
38	175	5.08	5.9	14
25	145	6.11	4.9	12
30	155	6.8	5.1	22
10	150	8.4		16
30	150	8.6	5.1	14
15	150	8	5.1	13
20	135	8.53	4.1	13
15	140		5.1	13
25	130	8.77	4.1	14
25	140	8	5.6	22
30	140	6.9	4.1	17
20	121	7.5	4.7	21
36	185	5.09	4.6	19
41	210	5.27	6.4	23.5
36	190	5.97	6	22
35	178	5.61	6.15	19.5
26	190	5.51	5.3	24
23	148	6.37	5.2	24
25	170	6.24	6	23
36	185	5.11	6	23
14	125	7.91	4.1	15.5
32	175	5.02	6.6	21
37	175	5.6	5.1	25
36	210	5.02	6	29
28	140	6.9	4.2	19
25	152	6.2	4.75	19
20	180	6.79	5.15	20.5
33	190	6.02	6	18.25
22	165	7.04	4.2	26.5

34	185	5.9	4.4	26
36	210	5	6.2	26
25	175	6	6.4	19.5
21	222	5.19	6	19
33	192	5.35	5.5	26
26	153	6.8	5.5	12
29	160	6	4.9	18
27	170	5.59	4.2	21
26	156	5.87	6.52	21.5
23	115	6.47	4.3	15
35	186	6.37	5.4	18
34	170	5.9	5.95	23
30	170	6.25	4.6	24
26	140	5.6	5.7	18
39	189	5.8	6	24.5
43	170	5.8	6.15	22.25
27	182	6.09	4.9	19.25
40	150	6.2	4.3	18
26	175	8.8	4.2	22
26	165	5.85	4.8	23.25
27	175	5.54	6	23.5
31	190	5.6	6	23
24	180	5.2	6	25
31	157	6.16	5.3	20
25	210	5.6	5.8	19
24	145	5.08	4.5	19.5
24	170	5.08	3.5	24.5
30	195	5.03	6.5	22.5
23	168	5.01	4.2	18
24	165	6.02	4.5	20.5
30	161	5.05	5.5	21.5
30	175	5	4.5	25.5
20	155	6.01	4.2	16
19	145	5.06	3.8	21
31	180	5.06	6.7	23.5
29	158	5.07	5.7	19.5
26	160	5.07	5	18.5
32	190	5.01	6.5	20.5
25	165	5.03	4.6	16.5
31	178	5.03	7.2	22.5
27	175	5.04	6	24.5
25	190	5.06	7	24.5
19	145	6.03	4.5	22.5
21	130	6.06	5.5	21
45	205	5.2	6	21
26	148	6	4.1	19
25	165	6.03	4.5	22
25	160	5.01	3.8	19.5
18	150	6.79	5.1	20
23	160	5.05	5	20
32	180	4.72	4.4	25
30	170	5.2	4.5	24
23	158	5.54	4.5	18

18	150	6	4.8	17
38	208	4.7	6.5	18
34	170	5.19	6	23
32	200	5.02	6	19
26	155	6.32	5.23	17.5
35	225	5.1	7.5	21
26	170	5.58	5.5	22.5
24	175	5.03	4.5	23
25	155	5.33	5.5	23
40	210	5.33	7.5	22
23	155	5.75	6.5	16
28	162	6.7	5.5	20
25	180	5.73	4.5	18
27	170	5.31	5	21.5
25	155	5.88	5.8	21.5
20	140	6.43	4	18
25	170	5	5	24
20	145	6	6	19
45	188	5.63	6.5	20
30	175	5.16	7.5	21.5
45	180	5.46	6.5	20
30	160	7	6	14
25	175	5.75	5.8	22
50	175	5.66	4.5	16.5
35	195	4.8	7	23
30	175	5.6	7	21
30	188	5.09	6.3	23
30	175	5.58	4	20
30	180	5.94	4	21
30	150	6.08	5.5	22
40	188	5.5	6.5	22
30	180	5.08	8.2	21
25	160	5.65	4.5	21
30	145	7	4	15
31	162	6	6.2	22
27	175	5.06	6	20.5
32	190	5.02	7	22.5
52	235	4	7.2	24
22	153	5.4	5.5	21
26	200	5.09	6.7	22
25	150	5.6	7.5	14
20	150	6.7	5	16
35	190	5.5	5.3	20
35	190	4.8	8.5	21
25	155	6.1	6	18
35	160	5.6	5.5	21
25	160	5.9	4.2	20.5
20	182	5.6	4.2	20
30	160	5.8	4.8	22
25	160	5.7	3.5	25
20	162	5.7	4.6	22
25	170	5.7	4.5	24
50	215	4.98	6.5	28

40	225	4.98	6.5	26
35	185	5.05	6.5	28
35	175	5.53	6.5	27
45	215	4.45	6	25.5
40	200	4.72	5	26.5
45	200	4.52	5.5	27
40	180	4.67	5.5	26.5
37	180	4.67	5.5	27
38	195	4.35	6	26.5
35	195	5.05	5.3	28
35	127	5.7	5.6	18
37	148	5.75	6.45	24
21	112	6.94	4.05	17.5
35	135	6.44	5.85	22.5
33	138	5.56	4.25	22.5
39	170	5.5	4.28	27.5
42	180	5.32	6.8	24
40	170	5.35	5.3	21.5
30	150	6.06	4.1	20.5
28	145	5.63	6.5	21.5
32	148	6.68	4.64	22.5
40	150	6.91	5.25	21.5
40	190	5.19	5.12	23
25	150	6.06	5.02	23
40	150	5.94	4.06	20.5
33	140	5.82	7.1	23.5
38	160	5.8	6.32	29
47	210	5.31	8	28.5
31	160	5.63	5.56	28.5
35	160	5.75	5.37	29
44	210	4.75	7.7	33
37	153	5.28	6.35	27
36	185	5.06	7.7	22
41	170	5.43	4.45	29
46	170	5.19	5.75	27.5
34	170	5.38	4.86	29
38	150	5.43	6.23	26.5
30	150	5.38	4.92	21.5
29	162	5.48	5.04	22.5
29	170	5.5	5.88	24.5
30	180	5.79	4.8	31.5
36	160	5.31	4.6	21.5
47	180	5.28	7.28	31.5
43	215	4.62	7.38	28
45	175	4.87	5.8	29.5
28	150	5.62	5.18	28
45	210	5.6	6	31.5
31	165	6.38	5	25
45	210	5.3	6.64	28.5
49	211	5.19	6.7	29.5
40	188	5.27	5.8	31.5
30	140	6.44	5.06	21.5
39	170	6.07	5.29	31

33	190	5.93	4.38	24
34	190	5.12	5.9	29.5
42	185	5.56	6.05	28.5
35	175	5.68	5.08	27
35	170	5.85	5.95	30
45	180	5.56	7.75	25
45	180	5.38	7.35	29.5
31	140	6.25	5.65	22
35	160	6.81	5.17	20.5
36	205	5.3	4.88	26.5
31	145	6.31	4.5	24
43	210	5	5.65	27
40	190	5.13	5.7	25.5
38	200	5.43	4.75	27.5
43	195	5	5.75	28.5
34	150	6.19	5.15	24
30	185	5.25	5.25	25
36	177	5.58	6.9	23
40	220	5.27	5.04	25.5
29	148	6.09	5.5	20
25	160	6.75	4.92	23
27	150	5.9	4.53	24
31	120	6.12	4.4	19
32	149	5.17	4.51	21
32	152	5.32	4.5	26.5
37	135	5.06	5.31	25.5
34	171	5	7.1	25
25	172	5.1	4.15	27
37	175	5.25	5.25	26.5
33	190	5.09	5.5	20
31	208	5.15	5	24
22	125	5.45	4.5	23
35	150	5.43	4.25	25
30	136	5.36	5	25.5
32	137	5.06	6	25
21	116	6.18	4.5	22
18	120	6.38	4.65	22
20	148	5.6	3.6	25
28	142	5	3.95	23
25	130	5.93	4	22
30	150	5.25	5.85	23.5
28	130	5.5	4.15	23
33	155	5.7	4.3	26
25	205	5.65	5.3	22.5
37	180	5.31	5.44	25.5
29	160	6	4.58	25.5
20	112	6.7	3.05	
30	141	6	4.02	
30	126	5.7	5.58	
29	151	6.2	4	
22	145	7.08	4.38	
30	115	6	3.7	
40	175	5.12	5.25	

20	148	6.8	4.7	
24	140	7.15	5.48	
33	170	6.31	5.27	
30	162	6.44	5.06	
25	145	5.25	4.75	
20	111	6.65	6.5	
18	125	7.05	4.51	
27	185	5.03	6.3	
23	150	6.16	4.6	
26	160	5.04	4.5	
26	115	5.87	5	20
47	190	4.84	5.25	21
30	180	4.36	6.9	22
27	158	5.63	5.1	20
26	185	5.24	6	23
25	115	6.02		18
53	165	5.18	7.1	21
25	140	5.12	4.1	20
32	177	5.18	5	24
28	150	5.14	4.3	21.5
33	160	5.05	5.7	25
21	140	6.13	4.1	18
22	125	5.63	4.7	20.5
38	175	5.17	4.7	24.5
39	185	5.13	6.2	22
31	195	5.5	7.3	24
31	165	4.5	3.8	26.5
29	140	5.6	4.4	21
29	142	5.06	3.3	26
18	125	5.34	4.2	23.5
32	160	5.09	5.3	24
32	125	4.91	5.8	22.5
35	170	4.59	4.4	31.5
24	130	6.2	5.3	20
33	165	4.5	5.1	22.5
32	172	4.91	6.2	22
43	195	4.56	6.6	22.5
25	153	5.25	5.9	20
36	193	4.94	5	23
37	178	5.85	4.8	21
47	202	4.63	7.5	40
30	167	5.4	5.3	21
30	170	5.1	6	24
32	180	4.34	6.6	24
40	152	4.87	3.9	23
30	128	5.09	5.6	21.5
33	145	5.75	5.2	20
25	120	5.04	4.6	22.5
36	185	5.4	5	26
35	170	5	4.5	23
45	200	4.9	6.6	27
33	135	4.45	4.8	23
30	185	5	6.6	25

28	160	5.8	5.6	21
35	150	5.45	5.6	22
29	180	4.94	6.2	21.5
35	180	4.92	5.6	24
23	128	5.7	4	19
31	160	5	6	21
34	157	5.17	4.3	25
30	148	5.68	5.3	22.5
40	175	4.95	5.7	27
35	170	4.95	5.1	27
32		5.8		
15	110		4.1	
30	185	5.72	5.2	29.5
38	200	4.97	5.2	30.5
38	205	5.15	7	31.5
27	170	6.07	4.4	21
33	195	5.3	5.4	27
31	160	5.37	5.8	29
45	210	4.82	5.45	25
37	205	5	6.93	28
27	200	5.34	5.24	26
29	190	4.84	5.8	27
33	210	4.98	5.5	27
36	200	5.76	6.43	24
34	160	5.12	7.45	28
47	210	4.82	4.94	30
27	195	5.43	6.47	26
26	195	5.333	6.02	30
37	205	5.09	5.9	28
35	195	5	5.44	29
27	150	5.82	4.7	29
41	180	5.44	6.5	30
43	240	4.75	7.05	34
22	190	6.06	5.5	
21	150	6.02	6.6	
46	220	5.13	7.2	31
38	215	5.53	7.4	23
40	240	5.22	8.2	20.5
37	235	5.25	6.4	27.25
25	160	6.5	4.95	21.5
28	170	6.4	5	28
38	210	5.12	7	26.25
34	160	6.59	5.2	23.5
25	160	6.28	6.8	19.25
25	160	6.28	6.8	19.25
25	170	5.75	4.7	28
37	210	5.32	6.2	27
33	198	5.62	6.1	32
44	180	5.82	5.5	27.5
24	130	6.18	6.4	27
41	230	5.27	6.8	33.75
38	200	5.3	5.85	30.25
24	170	5.81	5.75	27.5

28	170	6.09	5.4	27.5
21	115	7.9	6.5	17
22	165	7.5	4.8	25.5
25	180	5.66	5.8	24
28	161	6.15	4.6	26.5
57	220	5.13	6.6	25
25	140	6.44	4.5	19
36	215	5.4	5.3	28
32	175	6.01	4.85	26
29	170	5.13	5	27
20	160	5.88	5.2	27.5
23	150	5.94	4.6	18.5
28	155	5.06	4.65	22
22	170	5.53	4.75	29
26	170	6.78	5.4	28
31	180	5.78	5.5	29.5
33	220	4.75	7	29
27	170	6.54	6.8	17.5
29	180	5.48	4	28
29	170	6.04	3.8	26
18	130	6.82	5.15	19
43	215	5.25	6.5	31
30	195	5.15	5.5	29
36	210	5.03	6.4	32
25	165	6.97	5.2	26
31	170	5.5	5.2	27
25	180	6.22	5.8	28
34	220	5.66	6.4	26
30	165	6.53	5.4	24
46	180	6.1	6.2	27
21	160	7.16	5	20
34	170	5.75	5.25	19
36	190	4.94	5.81	19
35	180	5.92	6.14	18
35	170	5.44	5.8	29
37	185	5.84	6.2	31.5
27	120	7.76	4.3	14
33	170	5.75	4.21	27.75
46	205	5.65	5.75	26.75
35	200	5.43	4.8	20
26	135	6.6	5.74	21
32	145	6.67	6.23	27
37	200	5.3	5.64	31
46	205	5.07	7.03	31
33	200	5.74	4.94	26
36	170	6.77	5.4	25.5
35	210	5.67	6.6	28
39	210	5.91	7.2	25.5
37	210	5.4	6.2	31.5
28	135	6.36	5.34	18
41	188	5.5	7.45	9
31	178	5.97	6.15	14
24	140	5.5	5.1	22

32	158	5.7	5.9	19
25	130	6.5	5.95	
27	110	6.81	5.2	11
33	158	5.3	7.3	24
45	180	5.69	7.26	19
37	178	5.5	8.06	21
34	150		7.62	25
28	138	6.55	5.9	16
37	164	5.43	5.1	26
31	180	6	6.9	21
28	128	5.6	6.1	21
31	148	6.07	5.6	16
35	201	5.3	7.4	27
32	180	5.54	8.9	26
23	151	6.09	6.95	26
22	139	6.85	7.84	16
25	175	5.43	6.78	27
40	210	5.25	6.4	22
41	210	5.4	6.66	28
40	230	4.97	11.71	24
23	145	7.35	6.7	
38	190	5.34	6.46	25
44	162	6.55	8.7	
38	202	5.22	8.14	25
30	170	5.59	7.68	17
30	168	5.5	6.76	22
33	170	5.12	6.19	22
36	180	5.91	4.21	23
35	138	6.41	6.33	10
35	152	5.5	6.98	27
40	190	5.35	8.23	19
44	180	5.09	7.32	24
22	120	6.8	5.35	21
26	138	6.07	6.44	20
47	198	5.22	8.33	23.5
28	148	5.15	4.81	18
27	170	5.32	7.92	20
25	170	5.02	7.4	19
40	192	4.62	7.6	25
40	172	4.96	9.02	21
26	180	5.22	10.25	23
27	170		7.44	21
50	210	5.3	10.42	32
32	170	5.81	7.15	26
48	210	5.25	7.53	26
30	170	5.88	5.25	20
41	198	5.61	6.51	26
36	190	5.88	8.11	23
43	192	5.16	7.26	23
41	198	5.54	7.05	27
21	122	7.63	5.09	18
40	199	5.19	7.43	27
30	188	6.07	6.56	24

20	162	6.09	7.87	19
44	183	5.63	9.18	26
28	150	5.35	5	27
33	165	5.25	5.3	25
45	200	4.75	6.2	24
25	130	5.75	7	23
32	150	5.82	5.4	24.5
43	180	5.3	6.2	23
35	145	5.45	5.5	29.5
30	140	5.5	6.5	25
43	200	5.04	7.5	24.5
36	160	5.25	4.8	27.5
39	150	5.25	5.5	22
30	145	5.99	6.5	22
27	120	5.9	6	24
33	120	5.75	6.2	23.5
45	160	4.99	5.5	27.5
28	120	5.97	6.5	21.5
38	130	5.4	4.8	25
24	125	5.92	4.5	19
30	145	5.62	4.5	26.5
31	135	5.32	6.3	27
41	175	5	7.4	26
24	115	6.88	4.8	19.5
33	150	5.15	9.4	27
29	125	6.05	7.3	
29	150	5.1	7	25
40	130	5.5	4.6	24.5
30	130	6.3	5.3	19
28	140	5.55	5.8	28
27	120	6.22	6.5	19
35	125	5.75	6	20.5
38	145	5.75	5.4	22
30	125	6.1	5.5	19
46	180	5.53	5.8	26
36	170	4.96	6	26
26	120	6.59	5	21
30	150	5.5	5.5	23.5
37	198	4.63	6.5	26
30	150	5.7	5	21
29	150	5.41	5.2	24.5
37	165	4.96	5.6	26
35	150	5.16	6	24.5
23	115	5.78	4.1	24.5
26	150	5.63	4.5	25.5
30	160	5.43	5.8	23.5
26	120	6.14	6.1	17
38	150	5.05	7.5	26
34	140	5.51	6.1	26
31	120	5.2	6.3	26
25	140	6.1	5.5	20.5
40	127	5.13	5.5	21
40	190	4.85	7	26.5

31	120	6.45	6.8	19
38	170	5.14	5.9	24
36	140	5.42	5.4	27.5
41	170	5.25	5.5	25
31	175	5.33	4.8	25
31	135	5.5	6.5	22
30	150	6	6	23
34	165	5.43	7	25.5
37	140	5	7	25.5
43	165	5.3	6.6	25.5
28	115	6.53	5	21.5
31	130	6.03	6.8	22.5
18	135	5.97	3.5	19.5
25	115	6.86	6	17
37	160	5.1	6.8	26
34	155	5.68	5.5	24
35	180	5.3	6.3	27
49	175	5.02	8.5	26
37	160	5.4	6.2	24
33	165	5.02	6.5	26.5
31	170	5.53	5.8	24.5
47	190	4.92	7	27
41	175	5	5.8	21
52	180	4.75	8.3	28
31	165	5.45	6.5	27
51	185	4.6	7	28.5
46	160	5.42	5.6	25.5
38	180	5.46	6	25.5
43	170	4.9	6.7	25
36	170	5.4	6.7	24.5
33	140	5.32	6	18
38	165	5.25	5.1	18
37	160	4.85	6.2	26
24	125	5.75	6	18
33	180	4.45	5.7	25
38	155	5.06	6.1	21
37	145	5.63	5.4	18
37	145	5.94	6	26
35	155	5.14	6.5	17
26	110	6.25	5.8	17
40	150	5.26	6.5	28
38	180	4.76		
22	120	6	4	27
37	150	5.25	7.2	24.5
30	160	5	6.2	25.5
35	170	5.55	6.7	20
21	130	6.5	4	
33	120	5.6	5	17
52	180	4.59	7.8	18
42	140	5.25	6	12
32	115	6.95	4.5	21
25	140	5.8	5.8	22.5
46	145	5.6	5.1	28.5

29	155	5.56	5.5	22.5
30	130	6.4	5.6	
23	150	5.51	5.6	21.5
28	130	5.5	5	26
40	165	5.33	5.7	21.5
37	135	5.08	5.7	23.5
31	120	5.8	5.1	25
38	160	5.3	5.7	23.5
26	125	5.75	5.1	23.5
34	180	5.32	5.1	23.5
39	130	5.1	5.7	27
38	120	5.65	4.7	23
31	130	5.65	4.7	22
41	120	5.42	4.2	11
22	125	6.85	4.5	19
42	200	5.19	5.6	29
32	185	5.19	5.9	14.2
35	190	5.47	6.7	22
35	180	5.41	4.8	26.3
36	185	5.56	4.42	21
35	147	5.41	4.3	16
35	180	5.04	5.15	28
34	180	5.53	5.5	23.5
46	194	4.5	5.36	26.4
30	175	5.5	4.11	19.5
34	165	5.25	4.15	24
25	170	5.66	4.2	25.9
42	205	4.75	6	20
35	170	4.85	5.3	18.5
30	170	5.72	4.4	26
25	150	6.69	4	24.5
30	180	5.06	5	25
23	120	6.4	4	19
30	150	6.35	3.6	17.5
25	175	4.99	5.4	21
36	210	4.88	5.9	28
46	210	4.81	7.5	20
30	150	6.15	5.85	21
42	191	4.6	5.5	29
33	165	5.12	4.7	28.3
33	170	5.16	5.7	20
39	160	4.65	5.4	23.5
30	140	5.22	4.2	23
35	185	5.15	4.3	26
40	210	5.19	5.85	25.5
35	170	5.6	4.2	25
30	170	6.34	5.35	18
30	130	5.87	4.2	19
40	180	5.44	5.5	22
25	165	6.29	4.5	26
35	190	6.12	4.7	26
30	145	7	5.5	20.5
35	175	5.7	5.8	27

35	180	5.13	6.4	27.5
33	180	5.6	5.5	27
30	135	6.67	5.25	18
30	160	6.15	4.7	26.5
40	175	5.5	4.5	31.4
25	175	5.8	4.22	18
40	200	5.53	6	19
35	180	5.6	5.1	26.5
30	130	6.7	4.85	20.5
36	180	5.9	4.92	21
30	170	6.32	4.1	24.5
30	190	5.36	5.9	27
32	200	5.4	5.5	25.5
46	200	5.03	4.7	27
36	200	5.3	5.43	30
48	220	5.26	7	27
40	220	5.5	5.5	20
23	165	6.21	4.5	20
53	220	4.85	6.6	36
32	180	5.85	3.7	13
30	210	5.2	9	25
25	150	6.2	4.65	14.3
40	155	5.5	4.2	20
45	210	6.5	5	12
40	210	5.7	5.7	24
42	200	5.5	4.5	20
32	195	5.85	6	20
36	195	5.6	4.45	25
34	170	6	4.5	27.25
35	215	5.38	7	27.25
30	165	6	5.1	28.25
63	210	4.5	5.7	28
31	175	5.18	4.3	25
37	170	5.16	5.1	21
35	185	5.06	5	21
40	180	5.27	6.1	19
43	210	4.66	5.7	25
35	175	5.47	5.4	17
26	148	6.02	5.2	
38	198	4.76	5.5	27
38	173	5.15	5.8	26
26	119	5.85	4.2	
24	140	5.72	4.75	21
25	165	5.69	4.65	15
32	150	5.48	7.45	21
27	161	6.25	7	18
23	120	5.95	3.75	
23	151	5.97	5.3	18
19	130	7.37	4.62	20
18	120	7.6	4.5	
36	185	5.11	5.92	20
35	190	5.23	6	23.5
25	155	5.54	4.52	17

25	150		5.35	
41	210	5.01	7.75	24
38	200	4.85	7.45	26
36	189	5.09	5.23	25
34	170	5.8	7.5	25.5
36	200	4.89	6.73	24.5
28	173	5.2	5.8	30
32	200	5.2	5.75	30
31	155	5.62	5.05	24
38	170		5.8	
36	160		7	
34	165	5.06	7.53	14
39	185	5.22	6.75	17
30	168	5	5.6	17
45	209	4.89	5.78	21
27	150	6.52	6.83	16
33	209	5.19	5.32	
44	230	4.57	7	
29	158	5.63	6	
38	189	5.07	8.32	
41	204	6.86	7.55	
24	145	6.22	4.5	
36	197	4.78	7.15	23
29	200	5.16	6.65	25
34	209	5.07	5.3	18
28	160	5.25	5.2	23
32	175	6.88	5.1	23.5
42	185	5.15	6.24	24.5
23	160	6.32	5.55	21
34	210	5.25	4.78	19
40	220	5.25	5.1	26
34	200	5.9	5.44	20.5
37	160	5.6	7.34	18
38	200	5.31	5.35	22.5
37	220	4.85	8.2	27
34	200	5.13	5.5	21.5
27	120	5.17	4.3	
36	200		6.85	
33	155		7.65	
42	170		5.85	
35	160		8.83	
43	185	5.1	8.26	23
30	175	5.75	6.35	20
35	250	4.84	7	27
36	175	4.87	5.3	23
36	160	5.66	5.8	19
36	190	5.32	6	15
31	185		5.35	24
35	210	6.59	6.71	21.5
50	215	4.98	6.5	28
40	225	4.98	6.5	26
35	285	5.05	6.5	28
45	215	4.45	6	25.5

40	200	4.5	5	26.5
45	200	4.52	5.5	27
40	180	4.67	5.5	26.5
37	180	4.67	5.5	27
38	195	4.35	6	26.5
35	195	5.05	5.3	28
35	179	4.9	6.3	27.5
30	161	5.87	8.35	32
30	172	5	4.82	26.5
22	165	5.07	6	28
27	180	6	6.2	28
27	160	5.39	5.56	28
45	210	5.2	6.4	22
42	200	5.2	6.71	25
43	200	5.17	7	21
30	180		8.4	17
40	210	4.74	8	20
42	210	4.93	8.2	19
48	180	5.4	8.5	
23	205	4.59	4	27
27	210	5.2	5.7	26
30	200	5.7	5.3	13
30	215	4.92		27
38	215	4.69	6.9	24.5
25	167	5.72	6.7	27
32	154	5.43	4.79	18
32	160	5.49	4.15	23.5
23	140	5.5	4.9	23.5
25	200	5.35	5	23
23	150	6.2	5.9	18.5
33	200	5.6	4.8	22
28	190	5.09	5.6	23
34	154	5.68	4.69	24
20	180	5.85	4.5	23
17	140	6.15	5.8	17.5
23	185	5.59	5.65	17.5
21	160	6	4	19
23	160	5.3	5.6	27
27	180	5.4	6.5	27
27	130	6	4.2	17
20	150	4.9	5.3	25
34	180	4.99	7.1	19
33	175	5.7	6.7	14.5
33	145	5.23	5.36	21
36	179	5.19	7.26	21
23	140	6	7	22
22	145	6.78	6	17
22	145	6.68	5.6	16.5
20	140	6.45	6.1	14
20	160	5.97	5.8	16.5
18	165	6.61	5.3	16
24	180	5.7	5.9	18.5
15	135	7.37	5.5	18

18	200	5.65	4.3	23
27	180	5.4	5	26
22	168	5.5	6	21
30	150	5.22	5.5	24
30	180	5.1	4.7	20
21	165	5.05	3.8	18
20	170	5.7	4.4	21
29	190	5.1	6.2	27
26	170	5.3	4.5	24
29	160	5.54	5.4	21
22	160	5.8	6	20
23	135	5.25	5.1	25
22	155	6	3.9	23
27	175	5.7	4.1	22
27	150	6.5	5	23
24	185	5.3	4.3	22
30	180	5.3	4.4	25
27	160	6.35	3.5	23
33	174	6.12	4.53	25
23	180	5.19	4.4	22
30	138	7.25	3.92	21
25	129	6.51	4.37	11
25	150	5.49	4.71	24
29	164	5.759	3.51	25
32	152	5.94	5.03	21
29	170	5.56	4.61	25
37	189	5.1	5.72	25
34	183	5.5	5.7	23
22	190	5.43	5.4	21.5
20	130	6.56	4.43	21.5
35	169	5.75	5.5	22
34	182	5.4	4.95	26.5
27	142	5.91	4.67	25
30	174	5.69	4.5	24
34	141	6.03	5.74	20.5
28	175	5.47	5	23.5
24	170	5.88	4.35	18
35	200	5.01	6.6	22
35	183	5.07	4.4	10
32	167	5.75	5.9	17
38	185	4.79	5.8	26
22	120	5.88	3.52	17.5
34	149	5.69	6.11	22.5
29	159	6.41	4.19	26.5
21	152	6.01	4.1	24
26	160	5.69	5	23
24	155	5.5	4.7	24
22	140	7	4.8	19
19	135	6.1	4.5	21
31	200	5.66	5.4	23
25	190	5.35	5.8	22
30	180	5.6	5.5	25
26	165	5.5	4.6	23

21	160	5.4	6.1	26
22	125	6.1	5.5	23
20	130	6	5.5	20
25	180	5.47	5	23
25	170	6	6.5	24
25	120	6.8	4	21
28	200	4.6	5.4	24
17	135	6.65	4	22
25	140	5.2	5.1	24
21	145	5.85	5.2	27
23	180	5.3	5.6	22
30	180	4.94	5.5	27
27	190	5	6	25
25	200	5.9	4.5	20
21	160	5.9	5	23
12	110	6.3	3.5	16
30	140	5.1	4.7	21
26	175	5	4.5	26
21	140	5.6	4.15	18
23	140	5.35	4.5	22
25	180	5.2	4.1	18
25	160	5	4	22
23	160	5.3	4	23
32	170	5.69	6.1	14
25	160	5.6	4.8	
35	180	5.1	5	12
33	180	5.19	6	12
	180	5.8	5.95	
34	180	5.9	5.5	15.5
26	175	5.88	5.25	14.5
22	140	6.5	5.5	23
22	160	5.4	3.3	21
23	170	5.5	4.2	20
28	175	4.9	5.5	22
22	170	5.57	5.1	28
26	170	4.65	5.1	29
37	170	4.6	6.8	28
20	150	5.4	4.3	28
24	185	5.19	4.7	28
21	170	5.25	4.7	28
26	180	4.75	6	29
25	190	5.3	4.6	28
26	180	4.96	5.2	29
26	200	4.95	5	29
23	150	5.3	4.3	28
20	141	5.7	4.6	28
33	167	5.64	4.71	28
31	170	5.39	5.3	28
39	190	4.38	4.11	28
30	159	5.29	5.34	28
24	159	5.65	3.26	28
27	205	6.8	6.5	28
40	180	5.21	6.23	21.5

40	195	5	6	19.2
45	190	5.66	6.4	25.5
46	168	5.5	4.8	27
46	190	5.53	6.02	24
30	185	4.9	5.2	31
37	153	6.72	4.24	31
30	190	5.2	6.2	32
45	175	4.88	8.7	29.5
21	203	5.12	5.5	30
25	205	4.95	5.5	30
45	215	4.5	8.8	25.5
52	258	4.37	9.6	26.5
25	175	4.84	6.1	30
40	189	4.52	4.23	30
50	210	4.34	7.5	27.5
26	120	4.15	5.3	28
32	195	5.47	4	29
34	240	4.58	7.6	28
22	150			
27	200	5.25	5.7	
26	185	5.59	4.6	
26	190	5.44	5	24
23	185	5.8	5.6	
20	120		4.2	
32	210	4.98	4.8	
21	170	5.98	5.6	13
36	220	4.48	6.1	30
34	210	5.35		
19	152	6.48		
40	210	5.31	5.2	27
17		6.61	6.2	
20	180	5.48	5.2	28
18	175	6.29	6.8	
20	90	9.7	4	
29	190	5.56	4	25
32	210	5.26	5.8	30
26	170	6.05	6.6	13
32	185	5.66	4.8	25
26	140	6.81	5.1	8
36	195	5.65	4.5	8
27	160	5.76	4.7	8
21	182	5.73	5.3	15
35	205			
29	154	5.9	4.3	9
27	145	6.5	6.6	9
18	135	6.69	3.4	16
16	155	6.5	5.3	10
17	150	5.8	4.7	20
24	175	5.5	4.75	13
	130	7.1	3.8	9
35	140	4.87	5	22
35	170	6.22	5.5	28
35	175	4.9	7	26

40	200	4.79	10	21
30	130	5.4	7.5	24
36	160	5.67	9.5	18
35	190	5.28	7	20
50	190	4.79	9.5	14.5
30	160	6.53	8.5	24
50	230	4.78	8	26
35	215	4.84	6.5	29
40	200	4.83	7	30
40	180	4.35	7	30
10	130	5.47	5	
31	200	4.91	7	30
41	220	4.48	9	32
10	170	5.66	5.5	
46	190	4.48	7.5	30
40	165	5.78	6.5	23
30	190	5.53	7	28
41	200	4.74	10	28
35	155	6.38	6.9	12
37	200	5.25	6.5	
40	195	5.16	7	12
45	190	5.14	7.6	
42	165	5.7	6.5	14
33	160	6.15	6	13
45	165	5.2	6.1	14
45	170	5.7	5.9	18
45	165	5.05	5.4	18
35	145	6.65	7	13.5
24	145	6.5	6	12
45	205	6.06	6.4	
45	155	5.97	7	
25	125	6.7	5	13.5
35	165	5.7	4.7	12
40	180	5.6	5.7	14
42	155	5.2	6	14
40	205	5.15	6.1	14
45	200	4.97	7	28
32	175	5.4	5.5	15
50	210	5.91	8	15
43	215	5.11	7.2	17
30	190	4.97	5.8	20.5
32	190	5.56	5	26
25	185	6.63	5	14
35	165	5.47	5.7	14.5
40	185		7.6	21
35	165	5.7	4.65	14
45	195	5.06	6	14
35	160	5.36	5.6	16
27	175	5.45	7	15
25	160	5.12	5.6	20.5
35	170	6.12	6.3	18
40	150	5.53	6	18.5
32	140	5.4	5.2	22

30	165	5.47	6.6	19
28	150	6.05	5.6	11
30	145	6	5	25
35	150	6.53	6	
45	180	5.93	6.2	24.5
28	140	5.67	5.8	20
30	150	5.97	4.8	21.5
40	170	5.57	6.4	25
35	180	5.44	6.4	24
35	200	5.46	5.7	19
40	185	5.47	6.6	27
35	180	5.18	5.6	22
35	160	5.35	5.5	20.5
35	150	5.16	6.4	18
35	175	4.98	6	18
55	190	4.89	7	27
28	155	5.6	4.6	15
30	145	6.8	5	14
35	185	4.97	7.4	23
30	200	4.87	7.3	22
30	145	5.27	6.3	19
40	215	4.79	7.9	19
30	195	5.1	5.1	24
40	150	5.25	5.6	16
38	160	5.25	5.4	24
30	170	5.29	6.7	16
34	175	5.1	5.1	21
39	200	5.48	5.5	
39	160	4.9	6.5	21
37	165	5.92	6	22.5
29	150	6	4.2	20
34	160	5.99	4	
32	140	5.94	4	22
37	165	6	5.9	20.5
35	140	5.37	5.3	16
36	160	5.6	7.5	15
34	165	5.35	7.15	15
38	210	4.7	8.2	23
25	130	5.85	5.1	9
25	150	6.68	6.5	
35	165	4.59	4.9	17
40	150	5.7	6.8	16
40	155	6.59	5	19
45	210	4.67	6	22.5
30	195	4.9	6	30.5
45	190	4.87	7.1	27.5
30	135	6.19	5.5	
30	150	5.65	5.4	
38	175	5.75	5.6	23
45	175	5.85	5.6	10
30	145	4.92	5.2	14
35	145	6.42	5.8	14
48	220	5.46	7.7	15

30	145	6.67	5.5	12
40	165	6.19	3	24
40	165	6.16	6	20
40	175	4.1	6.5	30
20	145	8.09	4.7	19
30	210	6.46	7.1	23
20	180	6.65	6.1	18
30	180	6.51	5.5	10
40	175	6.09	6.5	20
45	220	6.91	8.1	10
33	155	6.59	5.5	
42	185	4.02	4.7	14
25	120	5.39	4.3	17
50	190	4.9	6.6	24
40	200	5.78	8.5	22
45	220	4.69	7.7	27
35	200	5.09	6.1	27.5
45	190	4.94	6.7	27.5
30	140	5.81	5.7	24
40	215	6.02	6	24
35	185	6.16	6	24
45	217	6.14	7.5	26
25	150	6.02	5.5	21
40	210	6.08	7	26
30	190	5.7	6.5	18
30	190	6.06	5	22
30	150	6.27	6.5	20
31	165	5.51	5.5	12
31	180	6.53	5	22
30	170	6.3	5	22
35	170	4.9	5.5	15
26	175	6.2	6	24
23	175	6.47	5	22
30	170	5.78	5.56	18
30	195	5.05	6.4	27
30	188	5.9	7	26
45	160	5.3	6.7	21
46	185	5.27	8.5	21
34	162	5.59	5.5	24.5
33	188	5.39	4.55	21
26	162	5.31	6.4	24.5
25	165	5.61	5.5	17
25	165	5.61	6.1	30
31	173	5.83	5.4	28
30	165	6.59	5.3	23.5
30	145	7.29	5.2	24
31	175	5.2	6.6	23.5
38	212	4.86	5.7	24.5
26	165	5.23	7.1	21
22	140	7.16	6.5	20.5
40	185	5.35	6.2	26.5
40	200	4.79	7.8	26.5
35	185	5.23	6	22

39	182	5.2	6	21.5
	160	5.15	6.9	25.5
17	165	4.97	5.3	23
26	197	5.59	5.7	22
45	230	4.8	6.2	30.5
38	210	5.02	6.5	30
27	210	4.28	7.2	25
45	210	5.29	7.5	29
50	250	4.51	8	33
31	180	6.36	7.8	24
41	185	6.15	6.5	26
30	145	8.11	5	19
55	210	5.03	7.5	36
40	205	5.05	7	23
45	225	4.49	7.5	34.5
44	195	4.72	7	27.5
33	182	5.6	6.5	23
45	225	4.91	7.5	21
31	165	5.5	5.4	15
34	215	4.72	6.5	23.5
34	190	5.15	6.2	22.5
34	160	5.1	5.1	23
35	180	4.85	5.2	24
30	208	4.95	6	23
54	210	5.15	7.5	33
39	215	4.85	6	25
32	183	6.21	7.5	19
30	185	8.03	7.5	18
33	160	5.49	5.5	21
35	175	5.3	8.5	27
39	215	5.2	4	27
35	185	5.7	5.2	23
39	230	5.25	5.3	26
35	190	5.8	4.5	25
20	182	5.6	5.7	20
40	225	6.3	6.5	27
35	175	6.2	3.1	25.5
34	180	5.9	4.2	24
30	215	5.03	6.2	26.5
27	186	6	4.2	25
34	165	6.02	4.5	25.5
10	190	5.5	5.5	16
33	175	5.5	4.7	20
40	180	6	5	27
20	150	6	6.4	20
47	205	5	6	15
30	250	5.02	5.3	25
18	224	5	6.85	25.5
29	172	6.21	4.6	16
25	210	5.6	4	16
31	190	5.5	6.1	18.5
26	190	6	5.9	24
28	200	5.05	6.75	25

55	275	5.02	8.2	31
40	172	5.8	6	23.5
17	170	6	7.5	24
16	150	6.45	5	14.5
23	182	5.5	4.5	17
26	170	5.87	5.5	23.5
25	200	5.3	5	27
27	155	5.02	8	18.5
32	185	5.02	6.5	24.5
17	140	6	6	18
28	175	5.07	7	25.5
21	200	5.23	7	12
45	215	5.1	6.7	24
32	175	5.13	6	17
44	190	5.04	8.5	23
25	165	6.5	5	14
25	165	7.83	4.5	18
50	210	4.6	8.7	27
35	188	4.8	6	24.5
30	190	6.1	6.5	21.5
30	208	5.1	8	22.5
30	195	5.2	5	21
28	155	5.54	7	18.5
30	168	5.01	6.6	23.5
30	195	5.11	6	18.5
20	140	7.13	4.5	17.5
35	190	5.46	7	20.5
25	180	5.57	4.5	17
15	155	6.92	6.5	15
50	215	4.53	8.5	20
25	165	5.98	6.8	22
35	195	4.96	6.5	21
30	180	5.3	7.5	18
30	200	4.88	4.5	22
35	210	5.13	6.4	23
25	180	5.4	6.2	22
30	190	5.15	5.8	24
45	235	4.6	9.5	25
25	160	5.6	5	20
35	210	5.2	6.5	24
30	190	5.01	7	26
35	210	4.08	6.5	25.5
35	190	4.65	8.2	25
37	190	5.02	4.5	21.5
30	185	5	5.3	22.5
41	190	4.51	6.6	23.5
31	175	5.03	7	18
25	190	5.03	5.5	19
27	170	5.03	5	20
35	185	5	5.2	23
33	198	5	6	25
37	195	4.67	6.5	25
31	170	5	5	22

30	210	5.42	7	21
21	155	5.5	5.8	22.5
51	190	4.61	7.5	25
39	160	5.3	6	21
42	200	5	6.6	22.5
24	150	5.4	6	22.5
31	180	4.9	7.5	25
23	150	5.96	4.5	22
34	168	5.3	5	23
45	225	4.4	7	25.5
40	188	4.6	7.1	24
50	220	4.7	7.7	30
31	155	5.4	6	24
31	170	5.4	5.5	27
25	165	5.4	5.5	24
35	185	5.3	6.5	22
25	160	5.8	6	18.5
29	158	5.5	6	14.5
35	172	5.5	5.6	23
36	210	4.9	7	26
42	188	4.9	5.5	27.5
30	180	5.4	5.5	24.5
27	170	5.3	6	26.5
38	195	4.6	6.5	21
32	190	4.8	7	25
30	170	5.8	6	21
40	165	4.9	7.5	21.5
29	155	5.2	3.5	25
35	212	5.1	6.5	23
30	145	6	5.6	19
35	225	5	7.1	23
40	178	5.3	5.5	20.5
25	215	4.4	7	24.5
30	160	6	5	21
40	210	4.9	6.5	23
30	175	5	5	24
35	190	5.8	8	20
40	178	5.4	7.4	18
30	200	5.4	6.2	26
35	205	4.9	7	26
25	175	5.9	6.3	20
30	145	6.1	4.6	20
20	140	6.4	7.5	16
35	188	5.1	6	25
30	148	4.6	6.2	18
35	185	5.1	6.3	21.5
30	185	5.6	6	25
35	210	4.9	6.5	24.5
30	170	4.88	6.5	22.5
31	165	4.63	6.5	19.5
37	205	4.75	6.5	21.5
38	220	4.46	6.5	24
35	198	4.91	6.5	23

33	180	5.35	6	18.5
22	155	6.25	5	24
37	195	4.85	6	22
50	205	4.4	7.5	24
27	195	4.88	6.5	23
37	190	4.53	5.7	22
35	185	5.03	6	23
25	165	5.85	6	18.5
53	195	4.4	7.5	23.5
33	180	4.78	6.4	23
35	200	4.88	8	22.5
37	195	4.46	6.5	24
26	140	5.88	3.5	20
26	180	5.85	6.5	20
32	195	4.62	7	21
42	200	4.72	7	23.5
30	185	5	6.5	22.5
50	220	4.04	7.5	30
55	235	4.21	7.5	27
40	200	5	7	30
45	200	4.59	6.5	29.5
50	250	4.09	7.5	24
50	215	4.76	8.5	31
45	205	4.62	6	25
50	200	5.09	6.5	27
55	230	4.04	7	34
45	200	4.05	6.7	27
45	210	5.08	6.5	30
50	210	4.5	6.5	27
45	210	4.11	7	24
55	225	4.15	6	26
35	180	4.18	7	26
35	210	4.5	7	20
40	210	4.04	7.3	28
50	210	4.22	7.5	26
40	200	4.1	7	27
45	190	4.63	7	29.5
30	170	5.76	5.4	27.5
49	192	4.67	5.8	28.58
40	203	4.93	5.6	29.5
35	180	6	6.6	29.5
42	191	4.84	6.2	35
40	112	5.19	5.12	20.5
28	140	6.25	4.2	21
38	190	4.63	7.6	26
31	145	5.13	5.3	22.5
30	145	6.62	5.52	18.5
42	178	5.13	5.2	22.5
30	145	5.88	5.22	24.5
40	162	5.12	5	27
46	190	5.19	5.71	24
52	163	4.65	5.5	22.5
37	165	5.5	4.14	26

34	152	5.31	5.2	32
36	153	5.87	4.2	26
40	190	5.25	5.2	23
37	150	4.81	5.36	22.5
45	190	4.94	6.55	24
32	165	5.31	3.45	24
30	145	5.62	4.1	19
32	148	5.75	6	24
47	188	5.25	6.22	22.5
40	195	5.13	6.4	34.5
27	150	5.75	4.8	26
30	170	5.62	4.2	36
42	195	5.12	6.74	28
46	190	5.32	6.37	33
26	163	5.62	6.9	27.5
39	178	5.06	6.14	28.5
35	160	5.19	7.45	28.5
45	180	5	8	28.5
44	210	5.19	5.85	29
31	190	5.12	5.84	30
33	160	5.94	6.67	27
40	195	4.87	7.3	33
33	165	5.06	6.1	29.5
32	155	5.12	5.48	28
40	200	5.58	5.24	27.5
30	170	5.69	5.61	27.5
30	155	5.62	4.52	28.5
41	190	5.38	6	22.5
38	175	5.44	5.35	23.5
29	140	5.39	4.9	19.5
39	203	5.43	5.6	23
40	180	5.06	6.65	26.5
38	170	5.75	5.6	24
29	150	6.19	4.8	24
38	165	5.7	5.2	19.5
43	152	4.85	6.28	27.5
52	205	4.35	5.6	29.5
45	152	5	6	22
32	150	5.29	5.8	20
40	200	5	7.75	25.5
44	210	5.2	7.96	25
35	145	5.95	5.86	18.5
35	132	5.28	3.18	23
26	125	5.94	5.6	21.5
38	152	5.56	5.48	24.5
42	170	5.53	7.8	15.5
31	150	5.1	6.1	24.5
53	210	4.56	6.4	29.5
46	205	5.38	7.58	25.5
51	210	4.75	6.35	27.5
39	162	5.1	6.58	21
48	142	5.25	5.8	28.5
47	195	5	6.7	29

35	151	5.5	5	24
29	127	6.32	5	23
32	165	5.32	5.8	30
29	135	6.5	4.35	20
33	171	5.59	7.4	23.5
43	175	4.94	6.3	33.5
33	181	6.58	5	26.5
47	152	5.25	6.1	25
41	150	5.75	6.2	22
44	181	5.32	9.2	30.5
65	250	4.5	7.3	31
49	210	4.56	6.9	35
51	200	5.5	7.6	29
36	175	5.62	5.7	29.5
59	195	4.62	6.15	28
37	182	4.56	6.6	32
39	162	5.37	6.1	30
45	205	5.19	6	26
42	190	4.9	6.7	27
35	180	5.44	6.8	30
35	155	5.37	6.2	30
51	200	4.75	7.2	30
38	170	5.38	5.9	27.5
48	190	5.19	8.1	27.5
52	208	5.06	9.2	25
50	190	5.12	8.85	28.5
46	198	5.06	6.7	23
45	190	5.44	5.2	21.5
31	149	6.07	6.15	24
35	165	5.94	7.64	21
50	200	4.88	6.2	30
50	221	4.63	6.44	37
40	192	5.3	5.9	28.5
40	175	4.82	6.7	34
35	205	5.56	6.12	28
30	143	7.15	6.33	21
35	207	5.7	6.92	25.5
55	200	5.48	7.4	24.5
50	190	5.43	7.5	28.5
35	179	5.65	4.7	23.5
50	215	5.44	7.5	22.5
30	135	6.81	4.36	19
40	180	5.65	5.53	21.5
30	170	5.83	5	29
35	169	6.07	5.6	20
35	180	5.57	4.92	22
45	193	5.44	6.4	28
45	207	5.06	6.15	35
40	175	4.81	5.2	26
40	180	5.25	4.6	21.5
45	175	5.06	6.65	23.5
30	153	7.07	4.58	18.5
50	210	5	6.25	37.5

40	215	4.44	7.75	38.5
40	190	5.91	7.6	20.5
40	190	5.56	6.7	30
30	161	5.6	5	23.5
60	210	4.46	6.4	28
45	187	5	6	24
35	186	5.25	5.53	19.5
35	174	5.93	5.52	23.5
40	173	5.12	6	24
30	192	5.94	6.51	21.5
30	150	6	4.35	20.5
30	160	5.86	5.65	22.5
30	130	6.81	6.58	18.5
40	175	5.19	6.2	
41	175	4.81	7.1	
30	150	5.75	4.8	
30	175	6.12	5.7	
15	122	7.05	4	
45	133	5.18	3.15	
27	168	6.18	5.03	
35	160	6	5.46	
26	145	6.53	5.6	
37	192	5.06	6.15	
46	191	4.8	5.75	
35	165	5.37	4.7	20
66	235	4.4	7.7	33
27	180	5.01	6.4	28
30	185	4.95	4	23
16	150	6.69	5.1	21
32	220	4.18	7.2	30
42	200	4.87	7.2	31
40	203	5.46	5.9	20.5
30	140	4.98	5.1	19
42	210	4.91	7.3	31
35	180	4.09	5.9	26.5
31	165	4.78	6.85	21.5
35	230	4.34	6.2	29
40	215	4.18	5.8	31
45	210	4.88	7.4	29
35	170	5	6.1	23.5
25	154	5.7	5.7	22
40	180	4.95	6.2	26
39	180	5.07	7	24
34	160	5.35	6.2	20
48	210	4.07	7	29
40	200	5.14	6.3	25
21	120	6.38	4.3	19.5
20	118	5.5	5.3	19.5
38	199	4.59	7.4	22
33	200	4.44	6.4	30
35	170	5.19	5.7	30
37	182	4.5	6.6	28
38	165	5	6	20

44	210	4.05	6.5	31
48	180	4.47	7.1	27
32	177	4.66	7.5	25
23	138	5.6	5	23
42	205	4.5	6.6	28
40	197	4.63	7.15	26
40	220	4.56	7.4	28
41	188	5.16	6.2	27
38	182	4.69	6.2	27
41	165	4.91	7	18
32	160	5	6.7	20
40	190	4.45	7	30.5
40	170	4.9	6.5	27
38	182	4.75	7.3	20
40	180	5	7.8	21.5
38	190	4.9		27.5
28	170	5.02	7.2	22.5
36	158	4.37	7.2	20
31	165	4.38	6.2	26
49	215	4.43	7.8	24.5
32	180	5.13	7.1	21.5
44	210	4.5	8	22
43	185	4.16	8	29
29	145	5	7	20
46	197	4.35	6.7	21
34	175	5.01	5.7	22
42	170	4.6	7.6	22
31	187	5.03	6.5	21
46	205	4.15	8	25
19	125	5.56	5.2	17.5
36	160	4.53	7.2	27.5
20	130	7	5.9	20
32	165	5.66	7.15	
42	190	5.19	7.4	30
38	195	5.28	6.15	25
60	220	4.2	7.5	32
30	155	6.2	4.1	19
36	180	4.9	7.5	21.5
45	200	4.63	6.5	28
32	140	4.8	5.2	21
32	170	5.24	5.5	25
37	200	5.2	6.8	28
32	175	5.75	4.9	22
39	162	5.15	5.1	22
30	160	5.22	5.9	22
42	218	4.53	5.1	21
31	190	4.28	5.6	21
40	185	5	6.5	21
40	170	4.62	7.4	21.5
45	205	4.15	5.1	27
38	190	4.53	6.6	31
38	200	4.1	5.1	25
32	140	5	6.7	20

60	205	4.25	7.6	37
48	220	4.19	7.5	39.5
35	195	5	5.9	22
33	155	4.95	6.9	20
40	205	4.34	5.8	38
40	130	5.45	5.8	22
33	117	6	5.9	24
32	173	5.05	6.1	24
49	195	4.7	7	34
47	210	4.2	7.8	32.5
43	212	4.34	7.2	33
25	150	7	7.1	
46	200	4.3	7.5	32.5
65	227	4	6.1	40
63	220	4.09	5.5	33
38	200	4.2	6.7	26
25	155	6.1	6.1	18
35	186	5.12	6.8	23.5
43	170	5.75	6	25
50	225	5.41	6.2	24
31	190	6.22	5.2	27.5
26	190	5.97	5.6	29.5
47	240	4.09	6.6	34
37	210	5.32	5.2	28
28	165	5.88	5	30
32	190	6.03	5.3	25
20	130	7.32	3.9	19
24	130	7.06	5.1	18.75
26	150	6.59	4.57	20
38	170	5.57	6.01	25
32	180	5.5	3.25	25
28	205	5.15	4.45	25
35	210	4.75	5.77	32
28	170	5.19	5.5	29
42	205	4.97	6.41	28
38	210	5.24	7.51	24
50	230	5.06	8.4	35
33	195	5.43	6.51	29
35	225	4.8	8	31
45	210	5.32	6.34	28
32	210	6.32	5.88	35
35	190	6.23	5.44	28
	160	6.49	5.65	23
30	175	5.4	5.5	20
30	205	5.21	6.21	29
38	200	5.42	5.73	28
29	185	5.87	6.76	16
49	240	4.07	7.4	34
31	185	5.66	5.44	29.5
32	200	5.65	6.9	17.5
50	220	5.11	6.6	29.5
33	190	5.24	5.98	29.5
40	180	5.32	7.2	22.5

35	185	5.48	7.55	25.5
23	150	6.31	4.93	20
45	225	5.23	8	31
36	240	4.66	6.79	30
47	250	5.01	7.92	29.5
48	240	5.03	5.83	29
28	175	6.4	5.4	22
23	130	8.65	3	
30	180	6.06	5	33.5
29	200	5.16	7.6	27.5
31	165	6.56	8	15
20	135	8.56	5.1	
30	170	6.28	4.4	26
24	190	6.72	6	17.5
32	170	6.35	6.1	23.5
33	180	5.94	5.6	18.75
35	200	5.38	7.4	30.5
37	215	5.87	5.8	27
40	210	5.13	7	36
23	120	7.29	4.5	28
42	220	5.16	6	24
26	170	6.15	5.4	26
32	190	5.94	7	28
31	175	5.78	5.4	28
39	220	5.09	6	30
33	200	6.53	6	25
40	215	5.44	6.8	30.5
31	215	5.13	6.8	19
27	200	5.7	6	26
53	240	4.72	5.8	21
36	165	5	6.94	19
50	230	5.9	7.4	19
43	220	5.14	6.24	14
56	220	5.9	7.9	
37	215	4.88	7.8	33.5
32	210	5.22	7.4	32
34	200	5.84	5	23.5
56	245	4.12	8.5	40
38	220	5.47	8.6	32
33	190	5.78	6.4	25
37	220	4.72	7.2	30
39	230	4.6	6.8	33
42	240	4.56	9	28
37	220	4.97	6.8	28.5
31	220	5.69	5.2	29
23	185	5.17	5.4	29.5
39	175	5.7	7.45	18
39	210	4.57	7	30
42	200	5.28	7.05	27
34	200	6.25	6.2	26
35	210	5.31	6.3	28
36	210	5.22	6.2	28
37	210	5.75	5.4	34

36	210	4.94	7.5	33
55	205	5.03	6.2	30
41	200	5.37	6	21
34	190	5.84	6.4	26
37	175	4.94	7.2	27
37	190	5.44	7.6	32
38	170	5.47	7.6	39.5
41	205	5.31	6.6	30
39	220	5.13	5.8	25
42	210	4.14	7.2	36.5
46	215	5.37	7.2	32
37	190	5.34	6.65	32
35	170	5.47	6	30
38	180	5.65	6.25	30.5
37	160	5.38	5.4	27
37	175	5.98	7.6	30
31	170	6.3	6.4	25
39	225	4.75	10	34
37	215	5.25	7	30
50	225	5.03	7.8	34.5
37	205	5.18	8	29.5
42	225	5.9	8.4	32
41	210	5.06	7	34
40	215	6.09	5.7	20
22	185	5.59	5.8	25
24	170	6.19	6.2	27.5
33	194	4.5	6.92	23
32	145	6.4	7.5	19
38	210	4.78	10.44	24
39	232	4.69	11.3	28
50	200	5.25	9.3	24
32	180	5.32	9.4	24
42	198	5.63	7	26
55	232	4.81	9.47	26
51	232	5.33	9.7	25
40	190	5.38	10.4	26
30	135		8.24	
41	210	5.07	8.55	29
30	210	5.66	8.34	21
37	200	5.12	7.6	25
45	202	5.3	8.22	22
32	200	5.07	6.9	24
40	210	5.28	7.72	27
32	220	5.4	9.08	21
63	238	4.9	10.48	30
37	160	5.81	7.65	24
42	210	4.71	7.83	27
35	160	5.97	8.4	21
	100		6.1	
45	245	4.44	10.45	29
35	200	4.83	9.07	32
35	160	5.88	7.1	16
45	184	5.94	9.06	15

60	240	4.8	12.51	
50	212	5	12.25	32
45	212	4.72	8.15	28
60	245	4.53	10.3	26
36	172	5.31	5.6	28
33	180	5.42	8.8	24
29	180	5.62	9.15	24
52	238	4.78	10.7	26
45	222	4.78	9.45	22.5
43	200	5.8	10.9	21
55	200	5.22	8.93	25
36	180	5.85	8.4	25
31	148	6.72	7.23	20
45	240	5.07	6.8	25
40	190	5.03	7.58	23
43	204	4.91	7.6	31
38	170	5.88	6.04	26
31	160	5.97	9.66	
35	160	5.18	7.6	9
47	218	4.91	7.96	30
31	160	5.91	9.56	20
30	200	5.28	8.1	26
35	168	5.28	9.1	27
45	212	5.09	9.06	32
41	222	5.43	10.9	31
40	220	5.09	8.6	31
33	140	6.12	8.3	17
36	160	5.56	7.6	23
29	200	6.35	8.55	26
40	238	4.9	11.45	26
33	192	5.05	10.45	25
34	200	4.96	9.2	24
24	148	6.3	7.95	16
32	182	5.55	8.2	
35	200	5.1	9.1	20
30	168	6.02	7.8	20
45	188	4.8	9.4	29
31	120	7.5	6.6	
27	170	5.82	9	19
51	218	5.05	11.28	22
38	188	4.95	8.37	23
43	210	4.95	10.15	25
40	242	4.9	8.27	28
31	180	5.9	6.6	17
38	192	5.05	6.8	31
40	220	4.75	6.7	31
47	230	4.8	9.2	27
35	170	5.75	9.05	21
56	202	5.85	7.75	22
42	198	4.9	9.93	26
28	210	5.05	9.56	27
23	142	6	9.55	19
44	190	5.06	9.66	27

48	210	5.09	11.9	31
33	140	6.34	10.5	25
	220	4.81	11	31
36	200	5.15	7.3	20
30	138	6.78	6.12	18
38	201	5.44	8.4	23
51	152	5.25	10.1	24
40	190	5.2	7	31
53	202	5.38	9.45	22
	230		12.3	31
		3.8	10.35	
56	250	4.72	12.1	32
45	190	4.99	7.7	27
40	225	4.75	10.35	28.5
49	240	5.04	11.8	24
41	211	5.09	8.88	25
60	211	4.72	9.7	26
34	218	5	7.85	25
42	200	5.78	8.05	17
35	198	5.68	7.7	26
38	195		7.65	26
39	205	5.47	6.8	16
45	235	4.94	8.32	26
40	202	5.25	7.95	25
26	132	6.5	5	22
37	190	5.3	8.2	
28	110		4.33	18
45	243	4.75	8.68	27
37	160	6.3	6.67	
39	208	5.2	7.39	23
40	220	5.1	9.81	19
44	180	5.59	6.17	22
45	180	5.65	9.3	18
33	200	5.52	6.32	20
38	210	5.41	5.39	24
17	145	6.79	5.03	
27	148	6.84	7.71	24
30	180	5.81	6.73	24
46	205	5.75	6.27	22
37	210	5.16	8	28.5
31	138	6.37	5.7	24
34	140	5.57	8.05	20
52	210	4.97	7.2	22
31	159	6.09	8.84	21
45	200	4.92	7.37	25
40	188	5.19	8.24	21
44	208	4.97	9.97	19
40	179	5.22	5.91	24
35	188	5.51	9.69	22
31	198	5.56	8.2	21
47	210	4.61	8.4	26
39	195	4.94	5.33	26
50	198	4.98	7.58	27

25	160	6.1	7.29	23
40	202	5.6	9.67	8
54	240	4.78	8.42	28
39	180	5.44	5.89	18
35	192	5.65	5.7	26
31	160	7.5		22
43	230	4.96	9.75	28
37	170	5.38	6.84	19
38	200	5.37	9.76	28
35	218	5.28	8.5	20
26	188	5.62	6.51	20
40	200	5.19	9.13	25
32	179	5.72	7.95	14
33	179	5.12	6.93	16
23	160	5.59	5.5	15
36	152	5.87	5.4	18
48	188	5.25	8.39	27
27	130	7.38	8.69	18
25	152	5.7	6.6	19
39	170	4.7	6.8	19
28	155	5.84	4.2	21.5
42	175	5.53	6.4	23.5
35	125	5.8	5.6	16
45	185	4.87	7.3	26
40	180	5.81	6.5	27
37	175	5.1	7.4	25.5
33	150	5.69	7	18
36	140	5.05	6.3	26
45	175	4.81	7.2	26
48	160	4.9	5.4	29.5
44	170	4.75	6.5	28
42	115	5.5	6	24.5
50	150	5.2	6	30
32	170	4.94	5.2	28
45	185	5.16	8.1	24
40	200	4.72	8	28.5
43	190	4.62	6.9	26
41	175	4.5	8.5	26
41	170	4.92	6.1	23.5
52	190	4.62	7.5	26
46	180	4.78	9.7	23
33	170	5.57	6	28
29	160	5.41	5.5	26.5
34	155	5.6	5.3	14
25	170	5.5	5.3	12
38	160	4.94	6.2	26
39	190	4.85	5.5	27
38	155	4.6	6.5	29
42	200	4.78		21
34	195	5.45	8.2	23.5
48	210	5.45	6.5	24.5
40	220	4.65	7.5	26
37	185	5.1	6.7	21.5

35	205	4.95	7.4	23
37	200	4.32	7.5	29
22	152	5.95	5	23
35	180	4.65	5.8	31
50	200	4.44	7.4	29
38	190	4.5	8.8	28
32	180	5.05	5.3	21
50	200	4.57	8.1	25
34	175	5.28	7.1	24.5
34	180	6	5.6	22
33	170	5.35	5.5	24.5
40	180	5.1	8.5	25
30	160	5.08	4.5	13
53	200	4.69		
38	195	4.65	6.1	28
40	185	4.91	6.2	28
30	140	5.84	5.8	24.5
32	145	5.16	6.2	26
36	150	5.15	5.8	25.5
40	190	4.62	7.5	26.5
36	195	4.75	8	26.5
44	195	4.7	5.4	25.5
32	150	5.09	5.3	17
27	140	5.72	5.5	26
42	150	5.03	5.5	25.5
30	125	5.78	4.5	10
43	160	4.96	6.4	12
40	190	4.66	7.5	29.5
32	180	5.25	6.6	27
43	185	4.84	6.8	26.5
40	165	5.25	5.5	23.5
46	180	5.22	9.3	25
38	155	4.87	5.8	24
43	120	5.44	7	21
41	155	5.25	6.5	25.5
30	135	6	7	
41	160	4.94	6.6	25
40	160	4.78	7.5	26.5
27	145	6.12	5.1	17
50	180	4.4	8	16
49	185	4.84	7.35	25
41	155	5.81	7	22.5
37	165	4.94	5.2	19
27	135	6.06	5.5	19
36	175	4.78	7.5	19
37	160	5.7	9	
55	250	4.5	8.5	24
44	170	5.3	6.5	29
30	180	4.95	6.3	16
49	220	4.97	7.5	22
53	185	4.65	6.7	30
41	185	5.06	6.2	23
46	235	4.47	8.5	23.5

25	155	5.95	6.1	22
25	140	5.3	5.5	22.5
32	160	5.3	8.2	23
48	180	4.78	6	22
54	180	5	7.8	22.5
42	200	4.78	6.3	24.5
28	140	5.5605	5	24.5
56	215	4.59	6.5	27
31	190	5.8	5.4	21
36	190	5.25	6.1	20
43	190	4.66	6.4	28
41	180	5.1	5.5	18
24	158	5.53	4	20
34	145	5.4	5	13.5
34	135	5.53	5.5	26
42	155	5.81	6.7	22
34	145	5.77	4.5	19
48	210	5.12	6	14
41	195	4.75	5.2	16
30	125	5.65	5.5	26
29	158	5.37	5.5	13
42	205	5.38	6.4	18
34	145	5.83	7	12
27	145	5.3	5.2	13
44	225	4.5	8.5	25
43	180	5.1	7.8	
35	170	5.13	5.4	8
41	145	6.1	8.7	14
42	180	5.22	7.6	26.5
34	195	4.91	6.2	23
41	205	4.91	8.1	25
38	158	4.97	7.5	27
43	185	4.55	8	26
28	135	6	5.2	21
51	180	5.2	8.5	28
40	170	4.96	5.7	22.5
50	210	5	7	26.5
32	145	5.5	6.9	26.5
29	145	5.48	5.7	25
29	150	6.33	4.7	20
35	180	5.25	6.2	25
41	160	5.4	6.2	18
39	145	5.52	6.5	25
44	200	4.6	7.2	25.5
36	145	5.82	5.6	25.5
45	155	5.55	5.3	23.5
43	175	4.75	7.5	39
43	175	4.7	7.5	39
40	130	5.09	5.7	28
38	165	4.8	6	34
31	140	5.35	4.1	30
35	150	5	4.5	24
39	145	5.37	7	28

38	150	5	7	26
39	120	6	7.4	12
34	145	5.95	8.2	18
28	130	6.08	4.1	12
44	155	4.8	5.2	25
29	160	5.35	5.2	26
33	160	5.22	6	28
40	190	4.57	7.8	28
53	175	4.47	7.5	19
39	150	4.53	8.5	29
42	180	4.9	8.1	16
26	150	5.81	5.4	14
40	145	4.8	7.38	23
42	195	4.4	9.6	29.5
26	150	6.07	6.9	8
40	190	5.22	8.5	18
39	145	4.85	8	22
23	160	5.45	6.2	18
35	140	5.22	5	19
41	180	5.02	6.4	29.5
30	140	5.05	6.6	22.5
33	150	5.02	6.1	14
36	150	5.5	9.3	
44	205	5.05	7.5	27.5
26	160	5.55	6.5	25
40	205	5.4	8.5	8
42	150	5.55	6.2	9
35	198	4.65	6.6	27.5
40	175	5.07	5.25	8
46	165	4.95	9.5	28
46	170	4.5	8.7	22
34	150	4.7	6.2	11
41	160	5.53	7	15
35	160	5.35	6.3	9
24	130	5.25	6	10
33	175	5.45		
56	210	4.37		
53	210	4.33		
21	130	5.8	4.5	22.5
21	140	6	4	23
33	155	5.7	5.2	23
48	175	4.4	8	30
30	125	6.19	4.7	25
33	115	6.4	5	18
34	130	4.9	6.8	28.5
24	115	5.22	5.6	23
41	165	5.04	8	25
36	175	4.7	7.5	29
40	175	5.6	6.25	22
46	190	4.37	7.5	29.5
36	140	5.5	5.5	14
44	170	4.7	6.4	28.5
45	120	4.75	7	17

39	115	4.75	5.3	27
32	140	5.78	7.8	12
25	160	5.6	6.1	21
35	165	5.59	5.4	21
35	190	5.41	4.7	26
47	205	4.44	6.11	28.5
35	180	5	5.6	28
35	175	5.09	5.31	24.5
41	215	5.4	4.9	23
45	210	4.88	7.7	27
33	180	5.6	4.8	25.5
34	150	5.37	4.9	21
35	155	6.13	4.1	21
37	170	5.8	5.2	20
30	165	5.69	4.35	22.5
48	190	5.06	6.42	22.5
25	160	6.58	4.1	18
30	165	5.53	5.8	23
45	210	4.65	6.8	26
38	190	5.28	5.2	25
37	183	5.8	5	22.5
43	203	5.14	6.2	24.5
40	190	5.18	6	25
35	210	5.06	6.47	28
41	190	5.15	5.2	27
41	180	5.25	6.7	20.75
40	200	5	5.15	25.5
41	210	4.58	6.76	27
39	190	5.56	5.75	23
35	210	4.99	5.57	23
33	180	5.09	5.25	24
33	180	6.15	4.8	24
46	182	5.39	5.6	23
36	195	5	5.7	26
31	205	5.06	7.3	26
34	190	5.59	6.8	18.75
42	205	4.97	6.1	22
43	210	5.06	6.5	16
38	200	5.5	6.5	17
45	215	4.6	8.5	26
38	190	4.82	6.9	
22	165	5.87	4.9	27
25	145	6.03	5	16
32	210	4.72	7.1	25
42	235	5.17	7.1	16
26	162	6.07	4.8	23
39	190	5.7	6.3	25
60	250	4.12	10	29
49	210	4.8	7	24
41	200	4.91	6.8	25
40	210	5.11	6.9	30.5
52	230	4.42	8.3	30
40	210	5.18	6.6	20

37	180	5.76	5.6	20.5
30	180	4.38	6.75	25
35	175	5.42	8	20.9
25	169	6.27	5.25	17.8
35	200	5.29	5.8	25
40	230	5.31	7	25.5
35	200	5.3	6.8	29
50	205	5.09	5.8	31
32	205	5.6	6.85	26.5
43	220	4.81	7.8	28
40	195	4.78	5	24.5
52	240	5.13	7.8	24.5
42	220	5.15	7	32
36	175	5.6	6.78	19
58	250	4.7	9.5	21
52	220	5.5	7.8	30
40	170	5.1	6.8	22
43	220	4.66	7.45	24.5
35	172	5.06	6.88	23.5
40	175	5.2	4.8	24.5
43	232	5.39	6.4	26.5
35	180	6.2	5.2	24
45	220	4.97	6	21
30	220	5.1	6.8	25
30	230	4.2	8.35	26
25	170	5.9	3.35	
35	195	5.4	5.55	
35	170	5.85	4.35	22
40	180	6.16	5.5	
45	240	5	8.15	26.5
40	190	5.5	5	16.5
30	165	6.17	4	17
44	205	5.19	6.5	24.5
55	210	5.15	7.7	28
35	210	4.88	7	24.5
40	200	5.5	7	31.5
50	205	5.4	7.15	24.5
35	215	5	5.7	36.5
40	185	5.2	9	29.5
40	200	5.3	7.7	13.5
43	205	4.9	6.5	24.5
30	190	6	5.5	23
40	200	5.8	7.5	24
40	230	4.88	7	27.5
49	220	4.5	6.9	28
50	215	4.37	8	30
45	170	4.91	5.7	13
41	180	4.88	5.8	17
46	200	4.88	5.9	24
47	180	5.2	5.3	23
40	172	5.27	5.84	
31	191	5.11	7	24
37	182	5.16	7.1	19.5

41	183	5.22	6.5	22.5
30	170	5.45	5.5	21.5
27	183	5.81	5	21
34	178	4.85	6.8	
34	199	4.95	8.45	
34	179	5.87	5.65	23
33	174	5.53	6	17
43	205	4.85	6	25
27	150	5.31	3.75	20
40	190	5.01	6.4	22
25	189	5.25	5.45	19
41	189	5.12	7.52	21
29	205	5.19	5	28
37	218	4.58	7	24
43	241	4.86	7.15	
32	206	5.01	5.85	22
45	235	4.71	7.22	23
44	210	5.02	6.82	25
42	230	4.94	8.4	25
38	220	4.91	6.54	22
41	230	4.91	6.3	24.5
42	210	4.91	8.8	24.5
27	180	5.48	6.18	19
32	200	5.41	6.7	20
39	230	4.98	7.45	22
31	190	5.01	5.2	18
28	150	6.68	4.55	17
37	210	4.68	7.66	26
33	180		5.1	
32	200		8.2	
35	200		6.32	
29	200		6.8	
36	240		7	
35	190	5.81	5.6	19
36	170	6.45	7.1	16
45	205		7.45	
51	280		8.63	
30	165		6.5	
37	190		6	
35	205		6.25	
22	140	7	4.85	15
36	220	5.39	8.05	22
34	155	7.16	6.1	17
40	210	5.2	6.62	21
43	220	4.82	7.65	24
23	131	6.83	4.65	15
45	240	4.59	7.42	23
30	170	6.12	5	16
25	170	6.19	5	21.5
30	160	8.71	6.35	14
35	180	5.47	7	20.5
32	190	5.31	6.5	24.5
44	205	4.76	7.2	

40	220	4.96	8.3	24.5
40	200	4.69	9.4	
47	245	4.42	9.1	
31	198	5.44	7	
32	208	4.72	7	20
42	185	4.72	8.65	16.5
29	200	5.12	7	25
43	235	4.67	9.22	24.5
45	255	4.84	7.15	
37	221	4.82	8	24
37	185	4.75	5.7	
44	245	4.52	6.22	
31	205	4.95	6.54	
33	150	5.47	5.8	
34	232	4.85	9.38	
25	161	6.12	6.25	
44	185	5.44	7.74	
35	212	5.2	6.2	21
24	145	5.29	7	
28	155	6.18	6	
32	170	5.36	7.5	
23			6	
40	180	5.35	7	
49	215	4.73	9.3	24
39	213	5.06	7	
38	205	5.06	6.3	
38	210	5.05	6	
25	176	5.28	5.55	26.5
24	152	6.62	5.2	23
35	196	5.78	5.75	24
43	230	4.79	7.3	
37	163	5.5	8.65	
41	178	5.5	5.22	
24	145	6.62	5.85	21.5
38	150	6.03	7	16
36	208	5.26	6.8	21
39	200	5.35	8.5	
46	215	4.85	8.39	28
37	195	5.53	7.6	
30	140	5.71	6.1	20
41	172	5.71	7.1	18
38	187	5.35	7	
37	190	5.13	7	
38	185	5.07	8	
51	218	4.62	8.8	24
42	240	4.62	7.1	23
43	210	4.75	7.9	24
43	208	4.48	8.8	
40	228	4.54	7.9	24
36	220	4.6	8.8	20
32	150	5.39	6.3	17
25	170	5.78	6.1	20
38	187	5.19	8.5	23

32	175	5.19	7.1	21
38	200	4.7	7.5	
33	170	4.88	6	23
36	180	5.56	6	
35	185	5.2	6	16
44	230	4.47	4.48	
37	187	4.78	7.5	
34	183	5.12	6.75	14
43	208	5.03	6.15	22.5
41	187	5.75	8.45	17.5
50	220	4.04	7.5	30
55	235	4.21	7.5	27
40	200	5	7	30
45	200	4.59	6.5	29.5
50	250	4.95	7.5	24
50	215	4.76	8.5	31
45	205	4.62	6	25
50	200	5.09	6.5	27
55	230	4.04	7	34
45	200	4.05	6.7	27
45	210	5.08	6.5	30
35	175	5.53	6.5	27
50	210	4.5	6.5	27
45	210	4.11	7	24
50	225	4.15	6	26
35	180	4.18	7	26
35	210	4.5	7	20
40	200	4.04	7.3	28
50	210	4.22	7.5	26
40	200	4.1	7	27
28	190	4.63	7	29.5
27	170	4.87	8	27.5
30	176	5	9.21	25
35	178	4.8	6.5	27
32	171	4.97	5.8	25
32	165	5.95	6.2	27.5
			8.19	29
48	215	5.31	6	20.5
37	210	4.9	6	16
25	230	5.62	6.4	17
42	200	5.99	7.8	
45	213	4.8	7	22
40	215	4.68	7.5	22
31	205	5.14	7.2	18
40	200	4.9	7.2	
42	200	4.69	7.5	25.5
46	202	5.03	7.4	23.5
50	215	4.3	5.2	17
43	204	4.14	7.1	23
32	220	4.4	5.5	22
41	235	5.6	8	15
37	220	4.53	8.4	20
36	200	5.05	8	15

27	190	5.36	8.4	17.5
35	195	5.4	8	18
40	180	5.19	8	17
42	198	5	8	22.5
46	190	4.88	8	
42	190	5.19	8	21.5
40	185	5	8	
52	215	4.72	8	24.5
51	220	4.78	8	
28	200	5	8	22
32	200	5.1	8	21
25	185	6.1	8	17
24	150	5.5	8.2	
38	200	4.9	8	18
32		6.2	8	16
32	200	5.38	8.4	23
30	215	5.2	8	20.5
36	205	4.7	8	
27	205	5.3	8	22
42	215	5.03	8.2	
42	220	5.9	8	14
40	210	4.8	8	23
55	200	7.43	8.57	14
41	208	5	8.65	14
43	210	5.12	8.7	
20	185	5.8	8.5	18
23	170	6.93	8.6	12
27	185	6.12	8.9	16.5
25	215	5	8.5	22
38	170	5.8	8.9	23
35	155	5.19	8.5	
30	200	4.99	9.1	14
35	190	5.3	9.2	15
20	152	6.16	9.2	
44	215	5	9	16
41	210	5.2	9	17
41	210	4.78	9	
50	200	5.09	9	
44	245	4.4	9	23
42	227	4.65	10.75	
43	195	5.4	10.2	
43	215		10	
50	205	4.9	10	18
40	210	5.1	10	20
40	230	4.7	9.5	22.5
20	175	5.9	5.25	16.5
26	185	5.48	5.65	20.5
39	210	5.05	6.6	21
35	215	5.62	6.5	19
30	210	5.1	5	21
28	210	5.29	5.4	20
30	210	4.68	7	25
51	161	5.53	7.94	18.5

35	215	4.8	7	25
30	210	5.5	5.5	21
36	220	4.7	7.25	24
36	215	4.99	6.35	18.5
39	205	4.9	6	28
40	210	5.1	7.3	18
32	210	5.5	6	23
25	210	5.16	5.2	25
27	220	5.05	7.5	21
37	210	5.27	5.1	23
36	210	4.65	7.5	24
35	215	4.9	7.8	23
35	220	4.9	7	26
25	210	5.75	7.5	21
24	210	5.36	4.45	27
25	210	5.3	7.2	22
27	215	5.9	5	20
27	210	5.88	5.8	21.5
35	205	5.2	5.8	21.25
30	210	5.32	6.5	24
28	210	6.1	6	22
35	210	5	7.5	22
32	205	5.37	5.1	25
30	215	4.65	5.9	26
36	205	5	5.15	21
27	215	4.92	7	25
25	210	5.62	6	18
35	220	4.85	6.4	24.5
27	210	4.87	7	23
22	137	5.6	3.94	25
22	175	5.6	6.2	19.5
12	173	5.96	6.1	21
25	190	5.24	5	18
26	180	6	5	22
25	180	6.35	5.4	18
25	200	6.05	6	15.5
28	190	5.76	6.6	17.5
25	205	5.02	7.47	24.5
20	185	6.2	7	12
25	180	5.5	6.1	20
22	185	6.2	7.6	13
20	170	5.93	7.5	15
35	190	5.36	6.2	22
35	195	5.05	7.6	21
32	205	5.84	7.8	19
35	198	4.77	7.2	9
24	180	5.89	5.1	20
26	180	5.18	7.2	20
20	155	7.3	5.6	13
26	160	5.86	6.2	21
20	175	5.86	7.4	17.5
25	150	6.82	7.5	13
20	165	5.93	7.1	18

25	185	5.82	6.4	17.5
25	190	5.59	7	22
28	200	6.05	4.9	18
25	180	5.35	6.6	19.5
22	185	5.92	6.4	18.5
22	210	5.03	6.3	20
25	172	5.9	6.3	20
25	172	5.9	6.9	16
25	180	5.89	6.4	17.5
25	196	6.88	6	21
20	200	5.35	7.5	20
20	180	5.8	5.5	21
28	195	5.36	5.7	21
27	180	5.76	6.5	18
27	200	5.75	7.2	21
30	215	5	5.9	25
55	227	4.63	6.7	26
53	223	4.65	7.3	26
27	185	5.52	7.85	20.5
30	200	4.85	7	25
25	200	5.1	5.5	21
27	200	5.2	7.4	22
24	190	5.63	7.9	14
25	180	5.4	5.5	19.5
35	155	4.95	5.5	23
22	140	5.4	5.5	22
30	195	4.8	6	25
30	205	5.34	6.5	26
33	180	5	6	22
26	200	5.05	5.1	27
27	180	5.45	4.9	27
27	200	5.3	7.5	23
25	180	5.25	6.2	24
26	185	5.5	6	21
39	200	4.91	6.4	23.5
37	195	5.47	7.5	14
41	160	5.34	5.73	25
37	137	5.75	6.8	18
36	134	5.27	6.32	21
38	189	4.64	6.45	27
30	190	5.09	5.5	21
34	180	6.2	6.8	17
32	175	5.84	6.5	17
37	193	5.93	7.5	19.5
34	200	5.1	5.65	19
32	185	5.1	7.5	21
32	200	5.2	7.3	24
32	190	5.94	7.2	18
37	195	5	6	27
36	192	6.16	6.23	22
38	200	5.37	6.75	13
30	190	5.18	6	22
33	200	5.05	5.5	20.5

27	190	5.45	6.2	23
26	145	5.3	6.5	26
17	140	8.23	6.15	12
20	145	7.31	5.1	19
24	190	5.52	6.4	20.5
25	190	5.37	5.8	17
13	150	6.3	7	20
15	170	5.3	5.5	15
32	190	5.78	5.8	16.5
29	200	5.48	5.4	17
35	185	5.28	5.9	19.5
32	170	5.35	6.2	23
22	155	5.5	5	24
20	180	5.8	5.5	17
22	172	5.84	4.6	19
28	185	5.62	5.45	18.5
25	185	5.75	5.7	20
14	165	6.2	5.4	16
22	180	5.79	5.5	19.5
26	200	5.47	5.8	23
35	175	6.2	4.9	18
28	190	6.51	4.2	19
25	180	5.9	7.6	20.5
20	175	6.1	5.6	15.5
27	190	5.21	5.7	21
18	160	6.95	5	11
22	145	6.73	6.05	13.5
30	195	5.5	7	20
25	190	5.7	6	19
27	200	5.9	5.4	22
21	180	5.6	5	18
27	200	5.62	7.4	21
32	190	5.2	5.3	16
26	172	5.8	6.1	15.5
28	175	6	6.4	14
28	195	4.9	5.15	21
22	125	6.3	4	
26	200	4.9	7	27
19	170	5.65	5	23
29	170	5	4.3	27
20	130	5	5.1	25
30	185	5.4	3.8	18
39	195	5.28	6.15	25
30	140	6.59	5.79	21
39	180	5.6	6.2	21
13	139	7.13	4.83	17
26	153	5.01	5.12	26
34	131	5.93	5.01	21
37	172	6.27	5.5	24.5
	160	6.1	7	
33	200	5	5.9	18.5
22	175	5.8	4.9	19
22	180	6.22	4.8	14.5

22	180	5.87	5.7	18.5
27	195	5.22	7.9	21
25	170	5.94	7	18
32	190	5.4	7	23
31	155	5.59	7	
39	182	4.84	6.5	17
26	160		6.5	22
15	160	5.1	5	17
20	165	5.4	7.5	19
30	150	5.8	5.3	21
35	200	5.6	5.5	22
38	157	5.28	5.71	16
28	150	5.15	4.25	
29	163	5.28	4.64	26.5
27	195	5.84	6	24
27	160	5.88	7	17
20	180	5.09	4.5	16
20	160	5	4.2	25
22	170	6.06	5.2	24
31	180	5.1	5.3	27
22	140	5.6	5	22
22	190	5.1	5.2	26
25	170	5.3	5.2	23
35	160	5.4	5.6	24
32	190	5.1	5	25
38	204	4.54	5.83	27.5
42	240	4.67	7.5	19
47	245	5	7.7	21.5
37	260	4.93	6.5	21
40	235	5.9	7	20
23	190	4.8	4.5	25
30	170	4.85	7.5	25
30	175	5.3	6.2	22
24	140	6.5	4.15	23
31	160	5.33	4.6	25
28		5.8	4.1	
35	160	5.98	7.4	23.5
30	180	6.3	6.5	18
28	190	4.9	5.8	
30	175	6	5	
32	185	5.6	5.2	17
33	190	5.1	6.2	15
35	200	4.9	6.4	17
23	155	5.8	6.2	13.5
33		5.4	5.4	18
25	190	5.9	6	13
21	170	4.8	7	24
34	235	4.75	7.3	29
34	240	4.57	7	29
31	165	4.85	5.3	28
28	170	5.37	4.1	28
47	183	4.82	7.17	28
48	181	4.75	5.79	28.5

44	184	5.11	6.72	28
40	174	5.26	4.9	28
26	190	4.84	5	29
31	190	4.7	6	29
22	160	5.4	5.7	29
25	185	4.87	6.5	29
29	180	4.78	6.2	29
27	195	4.89	5.2	28
27	170	4.9	4.7	28
30	185	4.8	5.7	29
39	163	5.35	4.64	28
26	165	4.75	4.8	29
35	205	4.97	7.3	28
29	187	4.79	5.2	28
25	165	4.8	6.25	29
30	175	4.34	4.5	29
30	210	5	4.95	28
30	200	4.7	5..5	29
31	220	4.8	6	29
36	220	5.01	6.4	28
25	200	4.9	6.1	28
41	210	9.91	6.5	28
32	205	5.2	6	29
46	210	4.84	7	28
30	230	4.85	6.3	29
42	157	5.43	5.9	19
40	177	5.69	5.63	27
42	180	5	6.9	25
40	160	5.59	4.7	26
40	174	4.94	5.1	27
44	187	5.71	5.43	19
40	135	5.19	6.15	20
40	172	5.9	6.2	14
43	187	5.15	6.73	23
40	186	4.9	7.95	26
43	197	5.18	7.1	
50	190	5.6	7.8	
49	181	5.03	7	20
45	223	4.47	9	30.5
35	220	4.6	9	33
30	190	5.06	5.1	31
30	210	5.05	6.2	31
58	254	4.12	8.4	31.5
62	255	4.22	8.1	30.5
53	260	4.7	10	32
30	200	5	7.3	32
45	205	4.6	7.3	35
38	218	4.72	6.75	35
51	247	5.12	6.18	35
48	183	5.1	6.46	35
42	189	5.25	5.67	40
30	200	4.75	8	30
30	220	4.89	8	30

57	231	4.41	7.58	30
35	225	5.1	6.2	30
37	210	4.8	6	30
32	210	4.95	7.1	30
28	240	4.37	6.7	29
29	220	4.45	5.6	28
56	179	4.41	5.67	27
40	240	5.12	8	25
30	220	4.79	6.5	25
64	275	3.97	8.65	29
52	218	4.67	8.5	26.5
30	220	4.55	9	28
28	170	5.55	9	25
53	220	4.53	9.5	25.5
55	224	4.63	6.28	30
63	200	4.84	6.38	30
30	200	4.84	6	30
40	188	4.68	7.1	30
31	190	4.5	7.5	30
50	237	4.56	6.48	30
48	198	4.63	7.5	30
46	235	4.9	7.5	29.5
50	208	4.7	7.46	29
30	185	5.42	5.2	29
31	185	5.47	5	20
39	250	4.6	8.5	28
23	190	4.61	5.6	20
31	210	5.17	5.8	22
20	145		6.1	11
26	195	5.26	6	13
26	145	5.94	5.2	10
34	210	4.87	8.2	30
23	165	5.99	4.1	20
34	195	4.83	4.7	18
27	180	5.37	6.2	25
45	230	4.96	5.7	25
24	155	6.34	5.3	22
23	150	5.66	6.3	13
34	210			
36	195	4.77	5.9	
40	210	4.87	6.2	
29	175	5.65	5.5	20
35	185	4.97	6.6	15.5
32	200	5.07	4.5	30
20	155	6.02	3	28
30	175	5.65	5.6	28
18		7.47	4.2	16
30	195	4.95	7.4	19
48	235	4.73	7.2	28
36	180	5.78		
38	235	5.56	6	31
36	210	5.32	6.6	9
32	193	5.11	5.6	20

27	170	5.53	4.7	14
22	170	6.23	4.2	23
23	170	5.92	5.7	10
40	200	5.34	6.6	28
16	145	5.78	4.1	8
36	235	4.78	5.8	17.5
12	120	7.38	3.1	
14	120	6.89	3.8	
28	190	5.34	5.4	15
24	140	6.3	4.8	20
27	240	5.3	5.8	27
25	194	5.03	5.9	23
29	175	5.27	4.4	26
40	240	4.69	6	23
35		4.95	5	25
39	240	4.42	6.3	30
33	210	4.73	6	30
29	180	5.53	6.8	28
21	145	5.48	6.9	12
38	185	5.44	6.4	28
22	175	6.02	4.5	30
32	208	4.89	6.6	31
20	145	5.87	4	
29	175	5.83	4.9	
28	208	5.68	5.8	
25	145	5.84	6	
30	235	4.86	6.3	28
32	190	5.68	6	11
35	220	6.94	5.4	23
24	175	5.45	5.4	13
26	175	5.07	5.9	25
28	170	5.71	5.6	14
29	170			
26	178	5.62	5.4	12
33	204	4.87	6.5	10
35	218			12
25	178	5.81	4.5	20
21	175	5.84	5.1	25
37	204	5.31	7.6	12
34	230	4.49	7	25
29	202	5.31	8	30
33	193	4.92	6.4	25
36	191	5.19	6.7	13
32	192	5.61	6.1	20
26	185	5.85	5.3	20
21	190	5.05	6.2	29
28	205	5	6.5	28
30	183			31
37	210	5.03	7.5	24
32			5.8	
33	218	5.16	6.2	
34	213	4.63	6	18
32	175	5.15	7.1	20

50	195	4.87	9	32
50	220	4.41	8	28
50	170	4.5	7	
40	215	4.9	7.5	13
45	220	4.83	7.5	24
45	240	4.45	7	30
42	230	4.4	9.5	38
31	145	5.91	6	20
40	220	4.97	6.5	21
45	210	4.77	8.5	25.5
25	190	4.4	6	28.5
30	140		9.5	
30	165	6	6.2	26
50	230	4.66	7.1	28.5
45	100	5.65	7	14
45	210		7.5	30
35	165	5.3	7.5	
45	245	4.4	7.1	30
50	220	4.75	7.5	
40	160	6.3	5	27
35	180	5.03	8.5	30
50	155	5.73	7	22
32	200	6.8	6.5	20
50	210	4.61	6.1	29
50	215	4.7	7.5	20
40	170	5.35	7.5	12
30	180	6.23	6.5	
45	150	6.7	6	20
40	190	5.97	7	24
50	220	4.97	8.5	30
40	220	4.86	8.6	24
45	235	4.35	7.1	26
31	190	5.8	7	22
40	190	5.53	5.5	26
40	210	4.5	7	28
40	210	4.9	6.6	24
30	150	6.71	6.1	16
50	200	5.7	8	22
40	200	5.09	7.5	24
35	220	5.15	7.5	15
40	180	4.6	6.5	32
40	180	5.3	7.5	24
40	180	4.8	6.7	26
35	170	5.73	5.4	24
35	190	4.9	6.1	24
40	165	4.67	7.1	24
45	210	4.97	7.5	28
35	190	4.8	7.5	26
30	170	5.21	5.25	24
40	160	5.6	7.25	25
30	145	5.66	5.1	25
35	170	5.28	6.1	22
40	170	4.95	6.1	25

35	140	6.17	5.4	25
30	220	5.6	8.25	28
40	160	4.7	5.6	26
35	165	5.2	6.2	21
40	170	5.44	6.5	21
45	180	5.91	6.3	19.5
35	160	6.43	6.4	18
35	165	5.16	6	18
40	185		10	
55	230	4.45	10	32
35	190	4.54	8	30
35	165	5.63	6.5	12
47	250	4.47	7.5	23.5
48	215	4.69	7.7	28
48	210	4.72	6.6	24.5
40	155	5.62	5.1	
50	240	4.73	7.65	25
46	195	5.05	7.55	12
45	230	4.8	7.5	25
45	215	4.78	7	16
40	210	4.85	8.6	14
40	195	4.7	6.2	19
31	140	6.25	5.4	12
40	180	5.13	6.1	14
40	172	5.5	5.6	15
50	225	4.56	9	27
47	195	5.75	6.9	16
60	240	4.82	7	15
45	195	5.75	6	14
45	205	5.46	5.4	17
45	175	4.81	5	13
30	135	5.6	6.6	11
30	130	5.11	6.6	12
50	220	4.5	6.45	22
30	135		7.65	
40	180	5.06	5.5	12
45	200	5.15	7	22
35	150	5.94	6.6	
50	170	5.5	5.3	17
40	190	4.75	5.7	28
38	180	4.93	6.5	16
45	220	4.5	7.1	27
40	190	5.19	5.6	22
25	150	6.67	7.2	
35	155	5.7	5.15	20.5
32	145	6.05	5.85	16
42	210	5.06	6.95	23.5
30	165	6.05	5.9	14
45	235	4.4	9	24
40	185	4.7	6.4	25
43	220	5.1	9.4	20
45	210	5.19	8.5	19.5
47	230	4.89	9	20.5

38	185	5.5	5.2	14
35	163	6.02	5.6	14
32	185	5.18	6.75	12
35	180	4.98	7.35	
45	190	4.54	7.65	24.5
40	205	4.83	7.35	20.5
48	210	4.65	7.05	23.5
47	205	4.6	6.35	24
30	170	5.68	5.6	26
35	190	5.22	6.1	27
43	235	4.53	7.3	30
45	200	4.91	8.7	28
45	220	4.28	6.1	27
40	160	5.15	8.1	27
30	140	5.25	6.5	
40	160	4.88	6	17
50	200	4.7	8.2	24
35	130	4.75	6	19
52	228	4.94	7.5	19
32	147	5.5	5.1	14
25	155	5.59	5.6	27
35	140	6.47	5	24
30	155	6.03	6	24
35	205	6.2	7	28
25	170	4.8	6	20
30	165		6	
30	165	4.97	4.5	26
40	192	6.01	6	32
25	150	7	5.8	19
25	200	4.73	6.5	32
30	180	6.17	6	30
40	180	4.77	7.5	24
37	170	4.9	7	26
35	195	6.17	6.6	22
31	155	6.65	5	10
20	140	5.1	6.5	24
35	215	6.84	7	24
40	165	6.4	7.5	20
40	200	4.91	6.7	26
60	230	4.43	7.5	30
37	200	4.9	6.8	22
25	140	6.51	7	24
20	140	6.74	5	22
32	220	4.9	8	28
20	160	6.11	8.5	26
40	190	4.8	8	24.5
50	210	4.87	7	
50	205	4.63	8.5	19.5
30	170	5.1	8	27.5
40	210	4.72	8	32
40	155	6.2	7	22
25	180	4.09	6.5	30
40	190	6.2	5	18

35	200	6.11	7.5	24
25	165	5.33	7.5	24.5
30	165	5.53	7.5	28
25	185	5.16	8	
50	200	4.47	8.5	30
30	140	5.86	6.5	16
25	200	4.5	9.5	28
40	200	4.4	6.5	28
40	230	4.55	9.1	32
45	180	4.73	7	28
40	200	4.74	7.1	30
35	165	5.45	6.5	28
40	145	5.4	5	20
40	195	4.83	7.1	26
40	175	4.98	6.1	24
20	155	5.65	5.8	24
35	155	4.95	6.6	26
45	200	4.5	10	26
30	140	5.79	7.5	26
40	170	6.06	6.5	24
38	185	5.1	7	24
40	180	5.06	7	28
40	190	4.85	7.5	26
50	220	4.01	9.5	34
40	195	4.55	7.1	28
40	160	4.95	6.1	26
45	175	5.17	7	24
40	200	4.66	7.1	26
40	215	4.45	8	28
45	215	4.69	8	26.5
55	155	4	8.7	28
40	240	4.81	9	24
45	220	5.04	8	27
45	190	4.6	7.85	23
45	230	4.97	7.05	20.5
50	185	5.07	9.15	16
53	185	4.88	8.35	25
40	160	6.08	6.35	
40	190	5.54	5.5	25
50	250	4.44	10	26
40	175	4.8	7	20
45	195	4.67	6.65	23
40	210	4.32	7.5	21
40	185	4.91	8	17
25	155	5.18	4.2	12
43	175	4.81	5.7	19
40	225	4.75	9	14
49	200	5.07	7.35	20
35	160	5.57	7.2	12
55	255	4.73		
50	230	4.53	9	22.5
50	240	5	7.5	19
52	230	4.77	8.5	17

40	220	5.81	7.9	20
40	205	5.05	11	16
45	210	4.44	7.5	18
30	150	5.9	8.6	
49	224	4.63	7.2	19
30	180	5.18	6.2	14
35	160	5.54	7.1	18
35	155	5.19	4.9	20.5
35	175	4.97	7.4	24
40	175	4.83	6.25	
63	270	4.13	7.2	27
34	190	5.18	7.9	23
45	175	5.19	6.6	15
52	193	5.44	5.2	19
47	235	4.19	8.7	15
45	230	6.63	8.7	17
55	250	4.56	8.2	22
35	205	5.12	7.7	19
39	185	6.2	7.1	22
30	160	6.75	5.5	26
30	160	6.9	7	
45	220	4.85	8.4	24
35	180	5.06	7.4	11
40	185	5.4	6.5	18
60	235	4.56	8.7	22
41	200	5.18	6.2	15
34	173	5.85	8.1	14
30	215	4.85	7.1	28
40	200	6.09	6	26
40	210	4.6	7.5	34
40	205	6.03	8.5	26
30	200	6.17	5.5	30
40	200	4.83	7	28
40	210	4.41	9	30
30	185	4.68	9	23
50	190	4.65	8.5	13
40	210	4.66	7.5	26
36	210	4.9	8.1	20
40	185	6.1	8	18
50	215	4.44	10.5	25
35	165		9	
30	185	6.25	8	14
40	200	4.5	9.5	26
40	200	5.57	9	
30	180	5.41	8	22
30	165	5.28	5.8	22.5
42	208		6.8	
39	202	4.94	6	19
36	181	5.17	6.15	20.5
40	203	4.66	7.05	20.5
19	135	6.1	5.23	17
31	165	5.5	7.4	17
46	213	4.66	6.55	22

34	196	5.01	6.8	26
32	170	6.62	6.8	20
63	250	4.36	9.35	23
33	187	5.5	5.7	24
36	200	4.9	6.2	
43	194	4.66	7.94	24
35	152	5.09	6.1	24
35	210	4.48	9.4	15
32	170	5.64	5.92	18.5
40	246	4.95	8.27	22
35	210	4.4	8.22	25.5
42	203	4.68	6.88	22
34	171	5.4	8.38	21.5
35	217		7.43	
38	206		7.8	24.5
32	166	5.14	7	23
34	190	4.82	7.53	25
34	190	4.94	7.83	26
33	172	5.38	5.45	25
50	201	5.03	9.58	
41	178	6.43	5.25	16
24	162	5.43	9.4	21
32	170	5.03	8.5	26
30	150		7.36	
35	200	5.25	6.15	21.5
35	213	5.07	5.38	21
30	165	6.03	7.43	21
35	195	5.71	6.15	21
35	161	6.12	7.65	15
45	215	4.54	6.72	27
40	180	4.75	9.6	26
29	175	5.52	5	21
26	153	6.06	5.1	20
36	200	4.81	7.12	25
39	205	5	8.1	24
26	161	5.75	7	17
32	174	5.68	6	17
43	230	4.8	7.1	25.5
35	200	4.8	7	24.5
39	189	5.53	6.35	21
41	204	4.87	7.05	23
44	204	4.75	6.55	24
35	204	5.03	7.82	24
34	190		7.83	
35	160		4.83	
43	220		10.15	
35	195	5.19	8.67	22
30	172	5.83	6	21
29	178	5.43	6.37	24
27	134	6.24	4.5	17
24	118	6.51	4.9	15
28	110	5.59	5.1	15
20	117	5.75	5	23.5

18	137	6.13	4.7	22.5
27	162	5.66	4.6	23
17	133	6.19	3.7	23.5
19	145	6.45	4.1	21
19	132	5.91	4.8	20
23	127	6.08	4.4	26.5
26	172	5.77	5.4	21.5
19	125	5.18	3.8	19
27	150	6.16	5.7	18.5
24	150	5.44	5.8	15.5
26	167	5.7	5	28
21	121	6.51	5.6	20.5
23	114	6.43	3.6	18
17	150	6.75	4.8	25.5
26	121	5.69	3.8	25
27	150	6.01	5.3	25.5
30	155	5.77	5	25
19	152	6.34	5.85	20
31	159	5.62	5.05	20
29	174	5.81	4	21
26	161	5.69	5.8	25
25	168	6.3	4.4	21
30	150	5.56	5.61	24.5
32	152	5.94	5.25	21.5
29	139	6	5.35	20
35	178	5.56	5.1	25
20	120	6.67	3.32	20
23	143	7.22	5.6	20.5
25	147	5.34	7.1	25.5
22	123	6.15	4.5	15
22	130	6.8	4.73	20
28	121	6.09	4.86	24.5
35	196	5.61	5.32	24.5
23	140	5.32	4.3	21
16	116	7.27	4.15	19
26	144	6.02	5.35	23
25	149	5.52	4.9	23
20	108	6.11	3.3	27
26	156	6.02	5.6	21
19	129	4.94	4.6	23
25	149	8.14	6.3	23
25	114	5.93	4.5	25.5
23	131	5.46	5	25
25	145	5.71	4.8	25
25	171	5.13	5.98	24.5
28	160	5.94	6.6	24
22	136	6.7	4.7	25.5
30		6.55	5.1	18
25	110	6.02	5	20
27	150	5.15	6	14
36	160	5.02	5	23
20	100	8.23	3	
30	135	5.07	6.54	21

26	110	7	4.3	
30	145	6	5	19
20	119	6.03		
26	155	5.09	5.2	18
23	115		4.1	
31	125	6	4.5	19
27	105	6.21	6.4	22
27	125	6	5.15	22
26	140	6.01	5	23
17	105		6	
27	115	5.01	5.3	
33	140	6.1	6.1	
28	130	6.01	5	
24	135	6.06	5	20
39	170	5	7.1	21
34	150	5	6	22
29	130	6.09	6	21
28	155	6.05	5.5	20
21	100	7.04	6.04	20.5
25	130	6.24	4.85	22
27	145	5.01	4.67	20
16	110	6.06	4.6	19
29	160	6		
28	120	6.18		
23	110	6.15	5	22
20	110	6.07	4.93	22
25	120	6.09	5.5	20
34	170	5	6.22	21
28	120	5	5	19
18	120	6.2	4	17
26	150	5	5.28	16
28	140	5	5.9	
22	130	6.06	5	20
27	110	6.2	5	17
27	120	7.04	7	20
35	115	6.01	5.1	21
25	150	5	5	21
23	120	6	4.55	20
25	120	6		
36	130	5	7	23
37	130	5	6	17
25	120	6	4.4	25
30	130	6.09	5.5	19
29	140	5	5.5	18
28	110	6	6	20
28	100	6	5.5	
22	160	5	6.5	21
28	100	6.93	3.1	21
20	100		3.5	
20	100	6.18	4.5	24
26	140	6.05	4.6	19
24	150	6.1	5.1	17
24	120	6	4.9	20

39	150	5	5.45	24
17	130	6.01	4.5	22
28	120	6.11	5.3	25
25	100	7	4.35	22
34	155	6	4.95	24
22	110	6	3.65	27
34	140	6	4.7	25
24	120	6.1	6.5	27
24	90	6.17	4	26
26	140	6		
16	140	6.03		
20	110	6.69		
30	120	5.78		
26	120		4.5	28
34	110		4.5	24
27	110		3.15	25
31	120		4.55	20
24	120			26
33	145		5.25	21
23	149		5.85	19.5
13	110		3.87	10
17	112		5.05	
16	104	7.63	5.5	17
35	165	4.77	6.82	22
26	108	6.06	4.95	19.75
29	118	6.08	5.69	19
31	122	5.02	4.69	15
31	138	5.34	5.11	22.5
27	177	5.4	5.62	18
32	160	5.31	5.8	17.75
24	116	6.61	5.48	17.25
31	151	5.18	5.81	20
26	157	5.19	5.77	22
22	137		5.14	21.5
28	150	6.31	4.9	20.5
26	163	5.66	6.56	20
15	100	6.66	5.7	14
23	134	6.1	5.94	21.5
34	142	4.81	6	18.5
18	123	6.15	4.6	18.5
19	104	6.9	6.24	16.5
24	136	5.38	6.3	19.25
20	112	5.68	6.97	20.25
30	146	5.65	6.88	18.5
24	144	6.61	5.8	23
21	149	5.91	5.68	20.5
21	122	6.32	6	18.25
19	123	6.99	6.75	19
22	114	6.01	4.58	
26	128	5.15	5.64	17.5
26	163	5.51	6.18	23
27	127	7.55	5.15	20.25
26	120	6.12	5.45	18

28	160	6	6.78	21.5
31	145	5.66	6.2	22
33	140	5.84	4.25	16
33	130	5.46	6.5	18
23	110	5.89	5.52	17
22	110	5.71	5.5	17
25	90	6.88	5.2	14
27	110	7.11	6	17
38	165	5.7	6.2	17
25	109	6.38	4.5	22
31	120	7.02	3.5	23
32	160	6.82	5.5	20
37	120	6.53	5.85	16
22	100	6.53	5	19
31	180	6.01	6.5	19.5
40	175	6.05	7.7	22.5
33	130	5.5	4.5	26
23	110	6.32	4.9	20
30	100	6.35	6.8	19
28	110	7.36	3.4	16
22	130	6.38	5.4	17
25	90	6.88	5.2	14
23	90	7.21	5.5	19
34	150	5.8	6	23.5
33	130	5.77	4.5	24.5
33	115	7.84	5	19
19	90	7.37	4.5	13.5
34	120	6.65	5.5	16.5
37	130	6.37	5.1	
23	120	8.15	5	
25	100	7.65	4.7	19.5
30	130	5.34	5.6	13
32	140	6.6	6	20.5
25	125	7.31	5.2	15
24	110	7.09	5.4	16.5
34	130	5.34	4.3	20
26	110	6.87	5.5	17
20	115	8.12	4.6	16.75
30	140	6.52	6.4	19
20	139	6.72	5	18.25
25	145	7	5.5	13
28	172	5.81	6.9	18
20	120	6.62	5.9	21
29	155	6.72	5.9	20
15	140	6.53	5.9	20
25	135	6.47	7	14
20	130	7.41	6.5	14
25	150	6.1215	6	13
20	135	7.1	6.5	18
25	182	5.16	6.9	26
26	132	6.37	5.5	17
20	129	7.16	7	
20	120	6.68	6	15

20	120	7.22	6.9	14
25	150	5.94	6.9	23
20	143	6.25	5.9	21
20	145	6.18	5.5	19.5
15	115	6.16	4.8	15.5
23	140	6.73	5.1	7
27	145	7.1	4.2	18.5
30	142	6.29	6.7	18.5
40	146	5.6	6	18.5
30	180	5.86	5.4	17.5
25	130	7.35	5	20
30	152	6.59	5.15	19.5
32	160	5.75	5.35	21.25
35	131	6.75	3.35	21.5
22	147	6.15	6.2	19
21	127	7.47	4.9	14.5
31	115	6.71	5.8	21.5
26	150	6.58	5.6	22.5
37	135	6.61	5.55	19.25
31	144	6.15	5.85	20
27	145	6.6	5.6	21.5
24	133	7.3	6.2	17
32	160	6.11	5.5	15.5
25	133	8.54	5.39	19
12	145	8.25	2.6	20
19	143	8.86	4.4	22.5
13	150	4.72	3.7	22
18	157	8.4	3.7	22.5
22	106	8.57	3.3	20
24	181	7.12	5.56	20.5
25	160	7.73	3.85	19
18	138	8.1	3.8	19.5
15	130	7.73	5	19
22	120	6.55	5.01	17.5
24	115	6.7	5.3	18
18	110	6.84	5.1	16
28	155	6.25	5	20.5
24	124	6.37	5.2	17
28	115	6.59	5.58	15
	136	6.06	3.9	20
36	165	5.35	5.54	22.5
27	125	5.62	5	17.5
27	125	6.74	5.43	17
30	180	5.4	7.1	24.5
26	142	5.4	4.54	21
22	116	6.15	5.08	13
28	146	6.31	4.62	20
28	135	6.3	4.63	13.5
25	142	6.1	6.24	18
22	128	6.55	6	18
23	142	6.4	4.54	19
25	141	6	5.02	20
17	120	6.71	4.53	17.5

39	148	5.12	4.57	21
26	113	6.73	6.28	16
23	114	6.78	4.12	16.75
28	138	6.5	4.22	17.5
23	130	7.34	4.46	17.5
28	160	6.22	5.74	21.5
32	142	6	5.2	21.5
20	90	7.33	4.8	19
30	127	6.73	5.55	19.5
16	80	7.28	4.8	19
28	127	6.44	5.75	18.5
27	152	6	4.45	19
28	135	6.12	5.2	19.25
21	128	6.08	4.55	18.5
22	107	5.73	4.35	18.5
15	113	6.23	4.5	19.75
34	155	5.22	6.54	20.5
24	105	5.99	4.68	16.5
29	136	5.94	6.62	20.75
27	132	6.38	5.5	17.5
27	150	6.34	4.6	22
25	118	6.21	5.1	13
28	150	6.74	6.11	18.5
27	155	5.54	6.61	17.75
39	180	5.4	6.32	22
24	110	5.64	5.64	17.75
24	115	6.85	6.24	13.5
22	130	6.59	4.99	17.5
18	122	5.89	3.71	18.75
35	142	5.5	4.36	21.5
30	120	6.34	4.84	20
25	128	6.16	4.8	19.75
21	110	6.74	4.46	18.5
40	170	5.28	5.5	16
35	165	5.28	4.81	16.5
24	120	6.2	5.58	19
30	142	6.58	4.15	19.5
18	135	7	4.93	18.5
30	135	6.15	4.66	18
26	122	6.85	5.85	18
21	146	6.12	5.28	21
42	172	5.44	5.16	19
35	167	6.06	4.9	19
27	120	6.12	5.4	14.5
22	130	6.61	4.1	19
25	132	6.2	3.61	19
35	180	6.53	4.3	14
21	150	4.96	4.7	20.75
25	176	5	4.29	25.5
18	140	4.85	4.9	23.5
22	184	4.48	4.4	22.5
21	157	5.21	4.3	23.75
18	126	4.48	3.8	20.5

31	141	5.2	4.4	13.5
10	115	5.21	3.5	16.25
22	136	4.4	5	16.75
29	164	4.37	5	21
12	124	4.59	4.37	20.75
16	130	6.75	4.1	12.5
23	174	4.98	4.57	21.75
31	200	6.31	6.7	18.75
22	155	6.75	5.3	19
17	135	6.84	3.85	20
28	167	5.84	6.09	15.75
38	182	5.43	5.51	20.5
24	140	6.31	4.56	18.75
20	152	6.53	5.13	20.5
26	145	5.62	4.6	18
27	147	5.3	6.1	17
26	160	5.65	5.9	22
21	156	6.87	5.22	18
26	149	6.53	4.68	18.5
29	147	6.56	4.9	17
29	155	6	4.43	23.75
27	190	5.52	5.77	23.5
30	135	6.04	5.5	17.5
36	124	5.33	5.95	22.5
23	94	7.27	4.69	18
25	115	7.09	5	17.5
33	105	6.32	4.9	23
20	120	5.62	3.9	22
35	175	5.34	6	18
27	125	5.63	5.2	19
24	155	6.03	3.5	22
30	111	6.68	6	18
18	105	7.37	5	19.5
26	115	6.68	5	22.5
19	103	7.37	4.2	19
22	123	7.4	5.9	15
25	133	6.41	5	21
18	100	8.26	4	16
30	135	5.88	5.7	18
27	125	6.48	3.8	23
19	125	6.7	5.2	21
20	115	6.29	5.65	21
20	105	6.09	4.45	19
23	100	6.68	5.25	19
28	128	6.65	4.9	13
23	120	6.41	6.05	21.5
21	112	6.56	4.75	21.5
26	135	6.4	5.65	19.5
28	110	7.21	5.05	19.5
32	115	6.47	5.05	20.5
27	105	5.97	5.6	21
30	128	5.81	5.85	22
33	135	5.85	6	23.5

33	128	6.31	6.35	22
22	115	6.9	4.8	19
30	150	5.75	5.55	21
34	110	5.75	4	21
30	145	6.62	5.75	16
31	183	5.59	6.1	19
28	112	5.71	4.75	18.5
26	115	5.97	4.65	16.5
27	104	6.1	4.1	19
29	149	5.74	4.6	22
23	114	6.02	4.1	18
29	134	5	4.9	21
33	145	6.18	3.8	20
19	115	6.72	4.9	20
25	100	6.47	5.3	21
27	126	6.91	4	15
27	120	6.38	5	21
29	125	7.25	5	20
30	120	6.47	5	23
15	114	6.75	4.3	15.5
25	115	7.94	5.8	14.5
28	140	6.34	5	19
25	105	7.04	5	18
35	156	5.71	6	19
30	178	6.34	6	16.5
38	140	6.21	4.7	20
22	100	7.74	5	16
22	124	6.21	5.9	21
25	160	6.21	6	19
32	160	5.48	6.6	22
29	160	6.41	6.3	22
30	150	6.49	6.7	23
	160	7.18	4.5	19
23	120	6.37	5.8	22
20	150	5.36	4	21
22	130	5.58	6.2	21
25	150	6.13	6	19.5
22	180	6.03	6.7	22.5
25	160	6.36	5.3	20.5
22	140	5.53	6	21
30	150	5.48	6.4	21
25	130	5.22	6.3	21
22	150	5.81	5.5	20.5
25	130	6.48	5.3	20
35	150	6.48	6.4	20
25	150	6	6.5	20
30	150	5.38	6.2	20.5
20	160	7.21	3.2	17.5
15	120	7.05	3.6	18
28	160	6.41	4.2	21
27	150	6.53	6.5	18.5
	130	6.35	4.1	19.5
	120	7.21	4.7	18

	120	7.31	4.1	17.5
	170	6.38	4.6	19.5
	120	6.94	5.7	19
	127	5.48	4.2	19
	102	5.85	4.1	21
	130	7.09	5.6	18
	110	7.15	4.1	17
	145	6.31	6.1	21
	135	5.53	5.7	21
	105	6.98	5.2	19
	120	5.63	4.7	21
	140	6.92	4.7	18
	122	6.35	5	20
	120	5.81	4.5	21
	170	7.21	4.6	18
	148	5.36	5.1	22
	120	5.85	4.1	21
	180	5.21	5.7	23
	140	6.36	3.3	20
	130	7	4.7	20
	130	7.21	5.6	21
	110	5.49	5.6	20
	130	6.35	4.65	21
	145	6	4.9	19.25
	180	6.35	4.4	19.5
37	140	6.34	4.8	18
23	100	7.1	5	
23	133	7.04	6.1	18
25	150		5.5	
30	135	6.4	5	18
16	145	5.5	6	18
	122	5.85	5.2	21
	135	5.85	5.1	21
27	135	5.95	3.25	18
25	160	6.3	6.5	18
30	150	5.8	6	18
31	125	6.4	4.14	18
30	140	6.3	5.9	18
24	130	6.25	5.3	20
20	160	5.4	4.7	21
21	110	6.78	5.1	19
22	122	6.06	4.6	20
28	135	5.81	3.5	22
25	155	5.82	4.8	21
25	150	6.1	5.8	18
34	160	5.84	6	19
20	135	6.1	5	17
25	125	5.8	5.5	20
22	135	6.4	4.5	17
30	150	6.1	5.4	18
25	130	6.1	4	18
21	155	5.6	4.6	20
20	140	5.85	4.6	18

27	130	6.18	5.4	18
23	145	6.18	5.5	18
24	110	6.4	5.3	18
20	108	7.1	5.1	16
22	135	5.89	5.6	21.5
32	140	5.9	6.4	20
26	120	5.04	4.8	22.5
20	113	6.5	5.2	17
22	105	5.85	5.6	20
28	135	6.9	5.3	18
29	110	6.1	4.8	24
27	165	5.6	7	20
28	145	5.9	6.7	18
25	110	6.8	4.6	17
27	130	6.25	4.7	19
29	110	6.65	5.3	17
25	153	6.5	4.41	21
19	127	6.21	4.62	18.5
23	100	5.9	5.1	16
24	123	6.78	5	18
29	135	6.15	5.47	16
20	100	6.43	4.2	16
35	135	5.59	5.9	19
22	110	6.19	5.3	18.5
25	150	5.38	5.4	17.5
30	118	6.37	5.75	17
24	120	6.34	4.77	20
33	155	5.18	5.46	20
28	115	6.5	5.14	20
28	142	5.87	4.74	21
27	164	5.6	5.6	16
22	136	6.43	5.35	19
26	160	6.94	3.63	25
30	158	5.5	6.3	25
22	169	5.09	6.24	27
29	143	6.31	3.61	24
29	140	5.25	4.5	24
30	134	5.75	3.85	14
37	166	6.4	5.2	13
24	108	8.28	3.6	21
30	139	5.78	5.9	16
40	120	5.95	4.25	19
40	120	5.52	5.2	17.5
45	160	5.96	6	21.5
28	140	6.31	3.2	18
33	173	5.37	5.71	20
27	115	6.21	4.76	20
27	115	6.34	4.9	23
27	106	6.09	5.8	17.5
26	111	7	6	17
24	148	5.81	4.8	16
37	169	6.31	5.18	21.5
37	169	6.31	5.4	23

38	167	6.15	6	22
25	127	6.6	5.26	19
36	162	5.4	5.71	19.5
37	170	6.01	5.4	22
35	150	6.7	5.2	21.5
20	120	6.51	4.25	15.5
30	130	6.54	5.34	19.5
30	160	6.599	5.25	17.5
34	153	6.92	5.5	14.5
26	138	6.9	4.95	19.5
30	120	6.08	5.8	15.5
20	130	5.96	4.2	17
32	145	6.09	4.1	19.5
39	112	6.3	6	
29	126	6.07	4.92	15.5
32	145	5.68	4.6	22
24	134	6.26	5.08	20
28	155	5.8	6.3	21
28	143	5.87	4.5	21
30	120	6.34	5.9	17
30	120	5.25	4.8	21
36	120	6.32	4.15	15
23	102	6.87	4.4	18
35	130	5.82	5.8	15.5
30	120	5.79	4.2	21
25	165	6.35	5.4	19.5
22	105	7	3.4	22.5
25	110	7.5	4	13
24	160	6.91	6.28	17
24	145	6.47	4.92	21
24	140	6.78	4	21.5
32	160	6.1	5.05	20
25	153	6.6	4.95	18
23	110	6.69	4.3	17.5
25	140	6.66	5	17.5
24	160	5.42	5.5	23
25	145	6.5	4.85	18.5
28	152	6.13	6.25	20
28	160	6.07	5.05	22
32	185	6.3	4	17.5
26	190	6.02	5.9	23.5
24	175	6.2	5.4	22
25	180	6.5	5	17.5
25	180	5.68	5.5	20
25	170	6.44	4	21
25	150	6	5.4	21
24	139	6.42	5.38	20
23	140	6.1	4.7	8
22	155	5.35	6.1	22
32	140	7	4.9	22
45	160	6.22	7	21
25	145	5.58	6.5	21
28	157	5.49	6.6	20

16	130	7.69	3.67	14
22	125	6.56	3.96	15.75
19	138	6.94	4	7
22	140	6.35	3.98	16.75
25	160	6.19	6	16
12	110	7.19	4.68	15
17	135	7.6	4	16.5
18	110	6.69	3.67	12
19	135	8	5	13.25
24	155	7.06	5.8	16.75
24	130	6.03	4.22	14.25
27	140	5.75	3.7	15
19	120	7.37	4.4	18
23	130	7.12	3.3	13
21	140	7.56	4.06	13.75
15	120	6.78	4.2	16.5
29	170	5.95	4.4	18.75
20	120	6.81	3.9	13.75
37	180	5.59	5.74	17.75
23	148	6.34	5.1	16.75
22	130	6.5	3.9	17.5
28	160	6.06	5	17.5
24	130	6.96	4.13	15.5
29	160	6.81	5.74	13.5
18	140	6.46	3.9	14.75
35	135	6.12	5	18.5
21	140	6.25	5	17.5
27	155	6.37	5.2	15
16	100	7.44	5	15
22	120	8.5	5	18
23	130	7.6	6	10
14	140	6.34	4.7	19
17	120	6.52	2.5	12.5
22	160	5.75	6.8	17
20	120	6.75	3.2	11.5
20	111	7.28	4.75	9
16	128	7.32	4	21
31	150	7	4.1	17
26	120	6.06	3.86	15
25	140	6.31	5.3	20
23	150	6.38	4.89	15.5
27	160	6.15	4.75	18.75
22	130	6	4.7	14
24	148	6.34	4.12	18
23	130	7.91	4.16	17
37	180	6.32	5.4	23
37	180	5.85	5.4	23
33	147	6.59	4.12	24
22	148	6.34	4.81	19.5
27	164	6.12	6	21.5
22	130	7.59	5	23.5
27	142	6.46	5	19.5
34	163	6.33	6	22.25

	170	5.88	7.3	25
25	129	6.99	4.8	23
35	144	6	6.1	26
32	173	6.33	4.16	8
25	145	7.22	5	14
30	110	8.5	6	15.5
35	150	6.31	5	23
	130	6.28	5.4	18
25	120	6.78	5	27
30	140	5.97	6	20
35	150	6.28	4.8	22.5
17	129	7.93	4.43	19.5
30	125	7.06	5.35	21.5
30	170	6.18	5.31	15
30	120	8.4	4	17
20	140	6.66	5.17	19
30	171	6.4	5	15.75
35	140	6.62	4.96	25
16	130	7.57	4	21
25	132	7.56	5	23
17	140	7.1	5	23
25	140	7.34	4.95	21
22	137	7	5.5	18
25	130	6.53	5.72	27
25	170	6.53	6.43	15.5
25	138	6.19	5.72	24
30	154	5.87	5.46	21.5
25	148	6.62	5	19.5
35	145	6.22	5	20
25	140	6.9	5	19
30	134	6.13	6	20
25	139	6.97	4.56	17
20	117	7.28	3.41	19
35	180	5.84	5.2	20.5
30	145	6.5	3.76	16
20	123	6.81	5	18.5
20	120	6.97	4.39	19.5
25	108	7.1	4	16
30	150	5.68	5.22	22
35	157	5.38	5.38	24.5
25	110	7.25	5.27	15.5
25	150	5.79	5	18
25	172	7.22	6	21.5
35	180	6.85	5.25	25
35	170	6.53	6.2	25
30	148	6.92	4.82	21
15	130	7.26	5	17.5
25	120	7.97	4.35	18
33	120	6.16	6	20
30	180	6.47	5.92	23
19	137	7.38	5	22
15	110	7.63	4	18
25	135	8	3.87	21

20	129	8.34	4.14	18
20	140	7.3	5.34	22.5
30	160	7.26	5.89	18.5
25	128	7.81	4.46	14
35	165	6.41	5.8	16
30	150	6.5	5.36	26
30	150	6.94	7	16.5
25	165	6.81	4.34	21
30	93	7.62	3.51	16.5
30	149	5.28	5	7
50	150	6.59	6	24
	149	6.22	6.5	23
	170	5.97	7	20
	160	5.96	6.7	23.75
	179	5.93	5.19	22.5
34	180	6.31	5	19
24	134	5.28	5.9	20
20	90	6.47	4.78	20
24	119	7.01	5.4	19.5
23	90	5.43	6.83	17.5
31	121	5.93	5.4	16.5
20	123	7.01	5.26	15
25	131	5.93	4.6	19.5
31	118	6.28	6.12	18
27	131	5.37	5.66	20
30	141	5.21	6.05	22
25	110	6.99	6.3	21
17	99	6.47	5.07	14
27	134	6.37	5.42	20
24	103	6.59	5.47	19.5
27	143	5.93	5.1	20
27	130	5.93	5.1	19
31	120	5.07	5.41	20
17	122	5.73	5.85	18
24	131	5.97	4.11	23.5
22	142	6.09	5.05	19.5
22	107	7.01	5.4	18
30	127	5.01	6	19.5
28	123	5.1	4.67	19.5
30	130	6.1	5.76	17
29	112	6.22	4.57	9
23	115	6.3	5.01	17
18	102	7.28	4.7	9
23	123	5.2	6.01	18
53	111	6	5.36	20
19	140	5.73	5	18
26	164	5.48	6.41	24.5
26	140	5.81	4.1	22
20	100	7.46	5.7	19
24	120	6.84	5.3	14.5
30	160	5.4	6.1	22
20	140	5.96	6.229	14.5
16	115	6.28	4.75	22

30	140	5.62	4.85	19
24	125	6.34	5.6	19.5
25	155	5.75	4.98	20
23	110	6.43	5.35	14.5
23	127	6.01	5.65	15
19	134	6.35	4.7	12
18	123	6.23	6.7	19
19	141	5.75	6.6	20
33	170	5.2	7.03	20
19	140	5.34	6.59	18
24	130	6.01	4.39	20
25	133	6.25	6.73	20
18	107	6.42	6.93	18
27	115	6.39	4.6	19
28	121	5.5	5.15	17
21	145	5.3	6.7	15
24	127	6.1	4.72	17
13	115	7.12	5	15
29	115	6.03	4	18
24	160	5.78	7	20.5
20	115	6.15	5	20.5
22	120	5.88	5.25	
27	153	6.16	6	15
31	150	5.84	5.73	17.5
26	118	6.31	4.22	18
29	131	6	6	19
23	109	6.88	6.1	16
22	111	5.2	4.68	
36	140	5.35	5.75	22
27	139	5.4	5.89	20
19	100	6.6	5.7	21
31	160	5.3	7	22.5
30	130	5.54	4.7	20
38	159	5.44	5.9	21.5
36	143	5.22	5.7	22.5
31	136	5.94	5.6	21
34	130	5.65	4.9	20.5
25	107	6.5	5.1	20.5
29	122	6.31	8.9	18.5
25	102	5.78	4.65	18.5
23	116	6.63	4.45	21
23	99	6.25	4	19.5
26	100	6.5	4.3	20.5
22	108	6.78	6.2	16
35	111	5.85	4.5	22.75
26	117	6.06	5.55	19.5
26	93	6.66	4.1	18
37	146	5.28	7.05	20
24	92	6.52	4.5	21.5
19	91	7.31	4.78	17
34	137	4.69	6.37	20
37	119	6.56	5.95	17.5
36	98	6.56	4.87	16.5

44	119	5.91	5.43	21
24	80	7.98	3.89	17
38	119	6.49	5.29	19
34	108	7.45	5.73	22
33	120	6.92	4.92	23
28	106	7.95	4.7	20
22	103	7.92	4.31	18.5
32	115	6.86	4.43	22.5
26	91	6.72	4.15	22.5
32	129	7.05	4.19	20.5
23	89	8.09	2.76	18
34	116		5.12	21.5
33	124	6.71	5	21
34	89	6.71	4.76	20
30	99	8.12	4.32	16.5
36	106	6.36	5.88	17
36	105	6.13	5.44	24
31	113	7.09	4.84	21
27	93		4.3	18.5
32	113	6.77	5.1	20.5
28	104	8.33	4.65	18.5
39	139	5.37	6.51	24.5
27	103	6.27	5.47	18.25
25	138	5.59	6.12	19
29	98	6.22	4.4	18
22	109	6.45	4.16	19.5
22	122	5.35	5.12	15
23	97	6.72	4.13	19
23	97	5.5	6.35	22
33	107	5.78	6.22	22
33	98	5.07	5.23	22
40	115	5.55	5	17
28	152	6.13	5.81	22
20	132	7.77	4.73	17.5
28	144	5.04	5.33	21.5
15	128	6.25	5.28	23
21	155	5.65	5.72	22.5
19	115	6.75	4.23	19.5
15	104	7.05	6	15
22	103	6.84	3.5	20
23	115	6.75	4.53	16.5
27	115	7.13	5.11	19.5
27	170	7.14	5.25	17.5
30	152	6.05	4.98	23
31	129	6.41	5.29	22.5
22	136	5.59	4.73	16.5
36	120	5.84	4.5	19.5
22	111	6.87	4.5	14.5
33	107	6.21	5.7	18.5
21	93	6.28	4.34	17
24	93	6.78	4.5	17
26	109	6.09	4.52	18
39	115	5.84	4.85	18

39	87	6.25	4.95	20
37	103	6.73	5.11	16
28	108	5.78	5.38	20.5
	116	5.68		
29	97	6.45	4	18.5
40	134	5.65	5.6	22.5
23	91	6.4	3.3	22
39	131	5.62	6.32	25
27	104	7.1	3.8	13.25
34	99	6.31	4.49	16.5
30	92	6.31	3.84	16.5
34	100	6.25	4.58	13.5
38	164	5.9	5.1	19.5
37	142	6.25	6.8	23
37	146	6.19	5.8	22
34	179	5.87	5.3	22
28	138	6.28	6.44	10
29	122	6.32	5.2	19.5
27	185	6.16	5.5	18.5
30	128	6.16	5.4	21.5
12	179	6.7	3.3	17.5
30	174	5.72	5.2	23
29	130	5.75	4.95	23.5
34	107	7.2	6.65	21
30	116	6.16	5.2	22
27	120	7.25	6.1	19.5
22	110	7.22	4.2	17.5
26	157	5.55	6	23
20	120	7.28	3.9	15
31	160	6.16	5	21
17	100	6.78	4	14
20	104	7.53	4.3	22
27	126	5.97	6	18
21	115	6.09	4.5	17
26	145	6.5	4.2	19.5
26	112	6.72	4	19
25	127	6.75	4.8	20.5
20	114	6.94	4.3	19.5
43	180	5.15	7	23.5
31	125	5.72	5.85	24.5
27	115	6	5	21
36	125	5.07	4.95	26.5
28	125	6.71	5.1	18.5
31	120	6.31	5.1	20
33	160	5.72	5.95	25
30	145	5.43	5.4	25
26	125	6.16	6	23
30	115	6.71	5.95	21
27	130	6.92	5.8	19
27	105	6.12	4.2	25
	100	7.19	4.55	22
27	120	6.17	5.4	22
26	113	7.19	3.9	19

22	127	7.12	4.5	19
23	149	6.87	5.1	
28	178	6.13	5.55	21
28	157	5.69	5.5	21
20	124	6	4.4	21
30	117	7.5	5	20
34	167	5.56	6.5	21
21	110	7.75	4.6	14
23	134	6	5	22.5
37	140	5.94	5.1	23
17	110	6.41	4.15	20
21	127	6.84	4.3	
31	167	5.69	4.8	22.5
31	137	5.75	5	18.5
33	163	5.88	6.6	19.5
34	156	5.69	5.5	21
31	152	5.62	4.9	22
32	144	5.41	5.45	18
28	124	6.38	4	21
25	152	6.1	4.6	
	135	6.19	6	
30	137	5.38	5.9	
	134	6.56	4.9	21
	140	5.63	4.4	
25	121	7.34	3.99	
31	138	6.81	5.56	
36	145	6.54	5.38	10
33	148	5.87	4.95	11
18	102	6.35	5.38	
31	128	6.13	6.5	
34	142	6.47	5.8	7
38	142	6.31	4.64	20
30	130	6.78	4.27	21
28	102	7.21	5.3	19.5
30	115	6.32	5.3	7
25	95	8.47	4.75	18.5
30	135	6.29	5.4	20
27	112	6.37	4.85	26
22	120	7.28	5.8	26
35	130	6.19	5.1	26
31	110	6.13	4.97	8
35	130	6.12	4.75	24
30	110	6.25	4.3	24
31	125	6.1	4.38	25
39	160	6	5.23	
36	155	5.81	5.2	17
25	145	5.5	5.18	17
25	100	6.82	5.85	18
22	122	6.28	5.6	
22	140	6.62	5.4	9
18	110	6.38	5	18
27	130	6.7	5	17
25	125	5.9	4.4	18

24	112	6.22	5.2	17.5
23	124	5.69	5.6	18
32	148	6.25	5.2	20
35	122	6.5	4.85	
20	130	6.56	3.95	22
25	119	5.88	4.8	7
25	112	6.69	5.2	
22	140	6.6	5.7	19
28	110	7.4	4.6	
17	110	6.69	4.8	8
28	125	7.06	4.95	11
21	104	7.44	4.4	14
20	135	6	4.9	11
28	126	5.91	4.4	
21	125	5.66	5.3	
27	135	6.78	5	
30	159	5.56	6.1	9
30	155	5.58	6.75	15
23	128	5.69	5.1	14.5
27	118	6.9	6.1	12
21	130			
25	150	6.73	6	25
20	118	8.22	5.8	21
35	188	6.08	6.3	24
20	95	7.59	5.1	19
19	105	7.7	5.6	19
23	124	6.93	4.8	20
20	160	6.8	4.6	24
27	130	7.1	5.15	21
21	138	6.56	5.3	23
21	128	6.96	5	22
23	116	6.96	4.5	20
21	110	7.76	6.2	18
24	115	7.23	5.2	19
23	120	7.67	6.1	19
22	154	7.39	5.5	23
18	116	7.64	5	18
17	147	7.19	5	16
31	144	7.26	5	15
18	147	7.68	4.58	20.5
20	152	7.87	6	20
29	182	7.36	6.6	24
21	126	8.15	6.1	18
24	136	8.1	4	21
32	157	6.76	7.1	22
21	137	6.9	4.6	19
25	140	8.16	5	15
29	142	7.35	4.72	18
	115	6.85		17
23	135	5.4	5.3	15.5
17	145	5.64	4	14.5
15	99	6.99	4.37	10.5
23	150	5.6	5.2	17

19	170	6.14	4.28	21
13	117	6.7	4.7	16
11	134	6.69	5.44	15.5
30	160	5.78	5.03	17
17	141	5.93	4	17.5
27	151	5.21	5.43	19
21	126	6.34	4	18
29	130	6.01	5	17
18	165	5.47	5.36	22
22	127	5.99	4.56	21
24	164	5.29	4.88	22
29	157	5.27	5.7	24.5
18	119	6.45	5	18.5
19	127	6.23	6.2	19
24	173	5.74	4.9	23
21	125	6.57	5.03	19
16	132	5.66	5.3	17.5
14	137	5.53	5.46	17
26	187	5.48	5.46	20
19	144	5.86	4.54	20
20	150		5.55	14.5
25	130	6.89	5.45	
30	145	6.14	6.5	
35	120		6.4	
25	120	5.38	5	25
20	115	6	6	17.5
25	165	5.4	5.2	26
25	100	7.14	4	27
25	130	5.54	4.1	30
43	140	5.63	5	
30	140	5.07	5	27
32	148	5.22	5.5	22.5
28	150	5.32	5.1	19
28	180	5.1	5	
30	165	5.2	4.2	
20	100	8.3	4.9	
45	150	5	5.9	
35	170	5.67	6	
30	150	6.63	5.5	18
37	135		5.5	21
30	160	6.26	5.1	21
35	180	5.86	5.5	23
40	160	6.63	7	21
35	155	6.25	6.1	23
25	140	4.84	6	19
25	160	6.14	5.5	22
30	140	6.46	3.7	16
23	130	6.5	5.1	12
36	120	5.34	4	
30	160	5.61	5.8	25
40	180	4.73	6.5	
35	150	6.07	6.5	18
30	130	6.3	3.8	

39	150	5.21	6.5	
38	180	4.76	5.2	20
45	180	4.52	5.8	23.5
35	180	5.44	7.1	24.5
27	130	5.42	6.2	
25	100		3.2	
35	130	5.13	5.88	22
30	170	4.69	5.7	24
30	150	6.43	5.5	21
30	150	6.13	5.2	19
25	140		6.2	
25	145	5.11	5.55	10
20	140	5.55	4.98	21.5
20	130	5.35	3.87	20.5
20	130		5	
25	140	5.3	4.9	
20	130	4.85	5.4	19.5
25	120		4.7	20.5
30	170	5.24	5.5	22.5
30	110		4.73	
20	110		4.5	20
35	140	5.21	5.15	20.5
30	140	4.76	5	21.5
30	140	5.41	4.47	13
30	140	5.13	4.68	
30	190	4.85	5.23	22
20	140	5.21	6.06	23.5
30	140	7.2	5.1	19
30	150	6.8	5	23
25	140	7.4	5.3	19
35	180	5.29	5.8	
42	180	5.13	7.1	
35	185	4.56	5.8	11
32	160	5.51	5.1	
35	170	6.18	6.3	22
30	160	6.8	6.5	16.5
30	200	6	6.5	18.5
30	152	5.63	6.5	18.5
20	135	5.9	5	19.5
25	150	7.21		18
30	180	6.2	6.4	23.5
20	178	5.02	6.5	19
21	100	5.8	6	22
30	120	5.4	4.5	19.5
30	170	5.28	6.5	20
30	162	5.9	7	18
30	150	4.89	5.5	19.5
30	155	5.8	6	20
20	125	6.1	6.5	18
20	118	5.8	5.5	19.5
40	140	5.27	5.5	18.5
40	140	5.19	6	18
10	125	5.9	5.5	18.5

15	130	6	4	19
20	120	6.8	5	22
25	155	5.18	6	23
35	140	6.8	5.5	23
30	175	5.28	5.5	19
35	165	6	6.5	19
30	130	6.32	5.2	20
25	130	6.34	5.1	23
30	110	6.5	5.5	29
15	110	7.16	5	23
30	158	6	5.8	25
35	164	5.73	6.6	26
23	130	6.12	5.4	20.5
30	160	6.3	6.5	18.5
28	130	7.57	4.6	15
30	150	5.98	4.6	18.5
28	150	6.84	5.7	20
22	130	7.09	5	13.5
30	170	6.63	6.5	23
25	140	6.42	6.1	15
27	151	6.27	5.05	18
22	165	6.65	6.6	20.5
28	128	6.65	7.3	17.5
25	145	6.85	5.9	19.5
26	125	7.41	4.7	20.5
21	130	7.52	4.4	18
23	121	7.88	5.4	18.5
20	110	7.23	5.8	
26	153	7.36	5.6	18.5
26	167	6.62	6.1	18.5
19	110	8.03	5.9	17
49	172	5.59	6.4	19.5
16	115	6.91	3.3	20
19	109	6.56	4.8	21
29	154	5.49	5.6	25
19	135	6.08	4.9	24
23	127	5.19	5.8	28.5
30	173	5.27	5.8	23
29	156	5.44	5.6	20.5
29	177	5.49	4.5	20.5
24	155	5.38	4.3	19.5
23	148	5.59	4.9	24
27	155	5.69	5.5	22
25	139	5.78	4.7	20
18	112	6.72	3.6	20
13	138	6	4.5	20
27	137	5.92	3.7	21
29	135	6.34	5.2	19.5
25	150	5.63	3.9	26
41	158	5.07	5.9	25
23	125	7.21	4	21
29	125	5.53	6.8	24
20	110	6.5	5.27	17.5

23	138	6.07	5.25	23
26	141	6.38	6.5	23
28	127	6.5	5	24
30	147	5.8	6.7	25
35	142	5.67	6	26
16	146	6.96	4	24.5
24	180	6	6.2	29
23	143	6.07	4.3	25.5
32	140	6.48	4.38	20
30	165	5.36	5.1	24
25	173	6.21	3.7	26.5
23	170	6.03	5.5	30.5
15	145	5.93	4.7	23
30	112	6.52	3.5	24
23	123	6.31	4.2	23.5
26	150	6.19	4.9	24.5
30	163	5.62	4.5	25.5
25	132	7.13	4.5	23.5
22	125	6.03	4.4	21
19	140	6.52	4.3	19.5
20	158	6	5.3	21
20	135	7.29	5	20
23	150	6.5	6.7	23.5
23	139	6	7.2	21
25	140	6.22	5.8	21
27	110	6.88	4.2	24.5
20	168	5.53	4.72	20.5
26	169	6.07	5.5	22.5
20	115	6.48	3.81	17
20	143	6.4	4.9	23
25	139	6.12	4.6	18
22	136	6.04	5.9	25
25	175	5.78	5	20
25	142	5.44	4.6	20
22	110	6.31	5.6	25
15	124	6.04	3.2	15
23	118	6.91	4.1	23
25	139	6	4.8	24
21	127	6.81	5.65	20
15	121	6.08	4.8	15
25	157	6.28	5.1	20
25	148	5.59	6.7	22
24	134	5.6	3.9	22
20	102	8.32	4.8	18
25	146	5.81	5	18
30	155	5.84	6.9	27
25	142	5.66	6	27.5
24	115	7.13	4.1	27
19	115	6.6	4	22
22	128	7.19	4.45	25
25	150	6.41	5.2	19
28	139	6	4.5	27
26	83	7.19	4.5	18

10	115	6.87	4.1	18
25	152	6.89	4.9	19
17	135	6.18	4.45	22
28	140	5.98	4.55	26
29	163	5.72	4.2	27.5
19	140	6.19	5.2	25
28	100	7	5	16
25	150	5.31	4.5	23
28	150	6.22	5.1	16
20	120	6	4.55	21
25	130	6	5	16
26	130	7	5.5	20.5
30	120	6.12	5.5	23
27	120	6.35	6.1	17
21	120	6	6	16
18	100	6.3	4.5	
25	100	6.03	5	19
26	130	6.01	5	22
29	140	5.1	6.5	20
35	140	5.11	6.25	18
26	130	6		
26	140	5.1	6.11	20
26	110	6.07	4.3	21
25	150	6.12	6.52	20
26	120	7	4.29	24
28	130	6.14		
17	100	7.1	4.5	20
24	120	6.11	5.93	21
22	100	7.11	5	19
25	105	5.01	4.06	19
25	120	6	4.34	19
26	120	6.02	4.7	22
26	170	5.01	6.5	18
32	180	5.3	7	22
25	180	5.06	5.5	23
32	125	5.24	6	22
25	160	6.31	5.2	18
28	130	5.3	4	
27	110	6	5	20
30	160	5.09	5	16
27	120	6.08	5.1	23
30	110	6	5	
32	140	5.1	6	20
21	115	6.29	4.5	
32	130	6.1	5.5	19
22	120	6.01	5.1	19
30	130	6.11	5	21
20	135	6	7	19
24	110	6.01	6	19
39	180	5	7	24
24	120	5.11	5.3	23
28	120	5	5.35	21
30	110	6.04		

30	100	6	6	20
27	130	6	4.9	
30	140	6.29	4.8	18
30	120	6.07	5.5	21
22	110	6.09	5.9	19
28	120	7.02	4.43	
24	120	6.09	4.22	20
25	100	6.42	4.91	20
27	150	6	6	
24	130	7.1	5.1	17
27	120	6	4.45	
33	120	6.01	6.1	
26	130	5	5.2	25
20	110	6.05	6.5	
26	130	6	7	18
23	120	6.02	5.1	21
26	115	6	6.55	21
26	110	7.02	6	22
28	130	6	5.5	17
31	125	5	6.1	18
26	120	6	6.5	
30	170	5.01	6	16
30	140	5.68	4.5	23
24	100	6.84	4	22
22	120	6.01	4.4	25
30	115	6.04	5.3	24
32	150	5.11	5.4	22
35	175	5	5.45	26
30	175	6	5.2	24
26	130	6	6.45	19
30	135	6	6	24
33	120	6.05	4.2	20
34	150	6.07	5.98	19
23	100	6	5.8	20
28	170	6.08	6.2	23
20	155	6	5.2	26
23	120	7.01	5.8	18
27	100	6	5.5	23
26	140	6.07	5.55	25
26	130		5.1	26
24	100		4.5	24
23	125	6.01		
24		7		
23			5	
22	120	6.18		24
25			4.5	
21	100		5.5	19
29	130		3.5	26
28	100			22
30	110		5.5	25
35	110		5.5	21
27	120			15
25	110			21

17	128		4.7	17.75
22	130		3.8	15.25
27	127		5.04	
11	137		4.66	
27	162		5.6	
25	135		4.59	
14	143		5.8	
27	160	8.64	6.1	
15	142	8.96	4.1	
24	160	8.76	5.92	
17	120	9.28	5.25	
23	116	6.43	6.24	21
38	159	5.35	6.35	17.5
31	125	6.01	6.1	17.75
38	165	5.8	5.83	20
25	154		5.51	20
25	127	6.21	6.1	
19	100		5.23	20
32	130	5.24	4.92	18.25
34	150	4.5	5.98	21
25	142	6.21	4.7	21
25	120		5	
33	129		5.36	
29	124	6.06	4.95	20.5
34	161	5.2	5.29	20
27	125	6.08	6.94	20.5
22	143	6.75	4.62	14.5
21	134	5.68	5.82	19
26	106		4.42	15
25	131	6.6	5.52	
30	128	6.67	6.15	20.5
22	117	6.59	5.12	18
37	143	5.2	7.53	23
23	120	5.85	5.62	
35	132	6.01	7.16	20.5
34	148	6.27	6.6	18
37	135	6.02	6.85	18.5
25	120		6	
30	142	5.61	5.16	21.5
29	103	7.8	5.01	11
33	141	5.35	6.97	
36	123	6.3	6.7	18.5
46	144	4.97	7.05	20.75
32	151	5.43	6.2	19.5
38	129	5.37	7.11	19.5
30	115	6.23	5.62	18
41	131	6.03	7.47	17
30	129	6.61	5.22	20
23	104	6.14	6	18.5
37	124	6.6	6.2	22
29	125	6.7	5.8	19.25
25	115	6.84	4.86	20.5
28	135	6.25	6	16.5

39	140	5.77	6	20.25
34	160	5.6	5.3	16.75
33	116		5.5	
31	127	5.83	6	17.75
31	134		6.78	15.5
19	118	6.71	4.8	15.75
23	150	6.77	5.85	18
26	132	6.9	5.4	17.75
25	130	5.41	6.2	17.5
20	138	5.06	5.1	18
16	103	7.17	5.1	14.25
24	151	6.38	5.7	20
18	115		6	16.5
32	159	5.94	6.25	20
25	129	5.79	6.5	16
25	129	5.81	6.5	16
31	103	6.41	4.62	14
32	139	5.22	6.81	20.25
35	189	4.72	7	20.5
29	80		5.33	
14	87		3.94	
	165	4.82	6.75	18.5
27	100	6.68	5	
32	100	7.37	4.5	16
20	100	5.86	5.8	15
32	170	6.2	5.5	15
30	140	5.96	5.4	17
25	140	5.45	6.5	18
40	120	6.32	5.5	17
36	110	6.12	5.7	18
42	120	5.8	5.6	17
36	140	5.6	6	17
21	110	6.1	5.5	18
40	140	5.96	5.5	19
20	110	8.05	5	16
39	130	6.01	7	19
35	109	7.18	5	20
30	140	6.68	6	19
22	100	7.28	4.5	16
29	100	8.25	4.5	16.75
35	110	6.4	5.75	19
26	130	7.02	5.2	18
30	120	6.75	6	18
27	85	6.95	4.8	20
45	160	6.5	7.5	20
34	120	6.35	5.9	21.5
43	110	5.26	5.1	24
32	90	7.23	5.25	18
27	120	6.75	5.75	19
37	130	5.68	6.25	20
21	110	6.56	5	16
28	120	6	6.25	11.5
36	139	5.9	6	17

20	95	7.05	5.25	15
31	140	6.02	5	17
40	120	6.32	5.5	17
36	110	6.12	5.75	18
42	120	5.8	5.6	17
20	110	7.55	5	16
38	130	6.01	7	19
26	135	5.77	5.25	17
27	120	6.84	5.2	16
19	150	7.06	5.8	19
41	140	6.11	6	24
29	160	5.85	5.1	22
25	150	6.5	5.2	19.25
29	120	7.56	5	17
30	150	5.81	5.3	23
43	145	5.34	5.5	22.75
37	120	6.03	5.8	18.5
37	120	5.5	5.7	18.5
31	120	6.25	5.8	17.5
26	93	6.63	5	18
30	135	6..82	5.5	19
25	120	6.13	6	11
25	130	6.66	5.5	20
41	135	5.84	5.25	19
28	150	7.31	5.5	20.5
18	90	8.81	5	15
41	170	6.21	6	19
22	100	7.65	4.5	13
31	110	8.68	5.2	21.5
32	105	7.12	5.6	15.75
36	150	5.41	5.7	20
26	110	7.84	4.1	17.5
35	110	6.25	3.4	20.25
21	110	7.21	3.6	15.5
20	110	7.3	5	16
25	125	6.9	5.5	21.5
32	150	5.65	6.5	20
15	125	5.94	6.5	16.75
26	180	5.43	7.5	20
29	160	6.12	6.7	21.5
30	165	5.4	6.5	14
25	110	6.67	5	17
27	138	6.17	4.9	17
25	135	7.09	5.3	19.5
29	125	6.62	4.3	17.5
25	155	5.74	4.5	15.5
23	130	5.84	5	20
38	162	6.29	5.6	22.5
30	120	7.53	5.3	15
38	152	6.35	5.9	18
30	190	5.64	5.1	18
39	157	5.97	4.75	14
37	140	6.52	4.3	19

30	156	6.1	6	14.5
30	130	7.4	4.7	22
25	168	6	4.75	21.5
25	120	7.4	6.4	16.5
28	135	7.3	5.25	20
28	128	6.74	5.2	20.5
29	122	6.29	5.25	21.5
28	105	7.16	5.7	17
17	105	8.15	4.2	15.5
29	145	6.8	6.4	21
27	145	6.22	5.7	20.5
31	168	5.95	5.3	20
20	124	7.27	5.75	21
27	148	6.1	6.4	22.5
29	121	6.84	5.95	21
28	115	6.89	5.3	18.25
32	150	5.97	7.05	21.75
19	138	9.34	3.7	20
14	143	8.47	4.6	17.5
16	156	8.53	4.5	19
20	145	9	4.6	20
21	150	8.19	6.74	22.25
21	133	8.94	4.52	22
20	143	8.19	5.97	19
18	123	8.37	4.49	19.5
18	134	7.63	5.46	17.5
16	146	7.28	6.37	17
15	130	7.65	4.56	
27	143	7.43	5.69	20.5
19	143	7.82	5.44	20.5
24	167	7.4	4.9	20.5
13	125	8.5	5.9	21
24	216	7.69	5.69	22
14	164	8.49	4.15	19
25	142	8.84	5	19.5
20	169	8.47	4.2	19
18	166	8.36	5.28	18.5
20	146	8.65	4.13	20
15	135	8.28	4.42	18
22	150	7.81	4.7	17.5
16	163	7.72	4.2	19.5
22	133	7.33	4.9	20.5
16	139	8.74	5.54	20
34	145	6.25	5.65	20.5
33	135	6.16	5.01	20
29	158	5.44	7.35	19
37	152	6	6.65	14.5
40	152	5.75	6.71	19
42	168	5.38	6.04	20
26	132	6.31	5.43	18.5
36	130	6.6	5.23	19.5
29	140	5.47	4.5	20
37	138	6.41	5.8	20.25

40	142	5.53	5.9	19
45	170	5.63	5.1	22
26	138	6.37	4.84	16
37	127	5.84	6.65	18
21	130	6.75	5.65	15.25
23	117	6	4.45	17
24	140	6.85	5.7	17.75
40	175	5.33	6.53	18.5
31	135	5.87	5.72	16
28	160	5.3	6.52	21
40	160	5.6	7.4	18.5
28	132	6.33	6.23	19.5
28	155	5.32	5.95	17.5
24	135	6.83	5.1	16.25
25	135	6.5	5	15.5
35	148	7.35	5.84	17
26	142	6.69	5.95	17.5
24	103	7.3	5.8	13.5
30	145	5.36	6.55	19.25
33	152	5.83	6	18
33	145	5.5	6.83	16.5
24	145	5.53	5.83	17
17	120	6.83	4.52	17.5
23	152	6.33	4.6	18
35	145	5.84	5.23	17.5
23	105	5.6	6.23	19.5
36	107	6.65	4.9	16
25	120	6.23	4.45	15.5
26	145	5.38	5.9	21.5
37	130	5.36	4.85	18.5
23	125	6.12	4.85	20
28	117	6.17	5.85	21
27	115	6.26	6.4	17.75
37	115	5.87	4.9	18.5
25	115	7.78	5.15	17.5
25	145	6.85	5.1	18.75
26	115	8.96	4.85	17
30	132	5.84	6	14.25
28	150	6.38	6.05	19
40	152	6.05	6.17	18
25	160	6.13	5.48	13
25	115	7	5.35	17
40	150	5.7	5.84	20
25	138	5.8	5.75	10
41	160	5.68	6.25	18.5
40	175	5.53	6.35	15
42	194	5.22	6.33	23
28	126	6	5.42	18.5
27	120	6.38	5.85	13
29	120	6.25	5.7	18
22	115	6.87	5.85	17
30	142	5.72	4.75	18
27	112	6.2	5.55	19

32	150	5.52	6.85	20.5
35	150	6	5..6	22.5
26	142	6.2	6.4	19.75
31	153	6.1	6.43	18.5
30	150	6.12	6.63	15
30	147	5.81	6.06	20.25
33	150	6.4	4.9	18.5
25	120	7.38	5.68	13
23	136	5.8	4.74	22
24	132	5.56	6.7	20.25
27	115	7.25	3.9	19.5
33	152	6.52	5.05	20
35	145	5.84	5	18
37	162	5.62	5.28	21
35	190	5.16	6.28	19
23	142	6.35	5.45	14.5
30	137	6.72	5.94	15
25	117	6	5.33	20
25	126	6.77	4.1	20
35	160	5.91	5.34	20
33	142	6.44	3.98	21
32	135	5.8	5.52	19
28	175	5.26	5.7	20
22	138	5.75	4.5	14.5
23	154	6.62	5	17
17	135	6.75	4.8	16.75
15	107	6.65	4.2	15.25
24	130	5.84	5.3	15
25	173	6.4	5.5	14.25
28	157	5.06	8	18.25
23	135	6.41	5	15.75
27	129	5.87	5	18
32	187	5.78	3.6	20
11	123	5.8	3.9	19
27	140	5.75	4.2	17.25
29	145	5.59	5.1	18.5
30	178	4.84	4.9	21
22	142	5.4	3.8	23
20	126	5.12	4	22
23	140	5.2	5.5	22.75
31	160	4.75	4.7	23.75
19	142	4.93	4.4	22.5
20	174	4.31	5	25.75
28	168	4.62	4.6	22.75
27	157	4.52	5	18.75
24	182	4.71	4.2	19.75
20	139	6.12	5	16
19	118	6.53	4.5	12
26	131	5.78	5.2	18.5
16	107	6.75	5	15
20	116	7.43	5	16
22	131	6.62	5	16
21	125	6.15	5.5	17

24	160	6.37	5.8	17.25
32		4.72	6	16.75
27	120	5.62	4.36	15.75
29	182	4.9	5.1	18
28	150	5.12	5.1	18
35	156	5.25	5.7	18.75
33	190	4.37	6	17.75
21	112	6.28	5.12	16.25
27	175	5.25	5.28	24
21	136	6.53	4.24	19
23	171	5.59	4.68	19.75
	160	6.3	4.36	23
25	145	7.51	4.27	17
20	155	6.68	4.92	16.25
23	135	6.62	5.67	18.75
20	121	7	5.8	15.5
27	154	6	4.95	20
16	142	6.5	75و3	17.75
16	122	6.4	4.2	14.5
29	143	6.18	5.98	14.75
23	160	6.52	5.53	17.5
33	180	5.47	6	18.75
24	142	6.28	5.28	18
34	114	7.3	4.55	15.75
21	144	6.54	4.06	15.5
16	117	7.2	4.5	15.75
25	132	5.46	4.05	17.75
20	114	6.8	5.2	17.75
25	122	6.46	4.71	17.75
23	126	6.78	4.99	17.75
26	172	5.87	6.97	19.75
24	179	5.81	5.96	20.25
23	137	5.93	5	18.25
25	142	6.75	5.07	18.25
24	158	6.84	5.68	16.75
20	130	6.96	3.92	20
23	124	6.86	4.94	16.75
25	154	6.4	4.15	18
23	168	6.9	4.64	20.5
26	150	6.9	4.76	18.25
32	154	5.75	5.3	20.5
29	150	5.93	4.96	20.5
22	149	6.18	4.82	22.75
23	164	6.7	4.74	18.75
28	152	6.76	5.52	22.75
27	137	6.36	6.51	21
33	129	5.73	5.7	19.25
19	100	7.45	5	14.5
27	189	5.96	5.5	20.5
21	124	6.5	5.3	20.75
16	122	6.71	4.1	20.75
22	144	6.8	4.97	20.75
27	140	5.96	6.53	18.75

24	127	6.5	6.37	17.5
19	150	6.8	5	17.25
21	150	7.16	5.49	16
23	143	6.4	5.11	15.5
28	175	5.93	6.52	18.75
28	172	5.5	6.1	19.75
25	140	6.43	5	21.75
22	166	6.3	5.63	18.5
28	170	6.47	6.32	16.75
31	110	6.53	5.51	15.5
31	135	7.46	5.53	17.75
25	145	6.78	6.52	17.5
31	134	7.34	4.69	17.25
23	150	7	5.63	18
31	150		3.64	
22	142	7.15	6.1	21.75
20	134	7.71	6.62	21
20	109	8.31	4	18.75
27	155	6.44	6.51	17.75
23	172	5.84	6.02	19.5
23	140	8.46	5.98	17.25
21	124	7.21	5.08	16.75
31	173	5.9	5.98	22.25
30	230	6.25	6.95	26.5
20	106	7.5	6.15	17.5
35	235	6.34	5	23
29	235	5.56	6.2	24
30	125	6.77	4.9	16
27	111	7.03	5.1	18
30	147	5.04	6	19
30	130	6.84	6.05	21
36	138	6.31	4.8	21
30	110	6.71	6.1	19
28	130	5.71	4.3	14
35	105	6.09	5	19
34	140	6.75	4.3	18
29	114	6.59	4.7	
28	145	6.38	5.05	24
15	100	6.01	5	21
30	145	5.78	6.8	19
25	135	6.12	5.1	16
22	125	6.47	5.05	18
22	125	6.47	5.1	18
29	125	7.53	4.6	18.5
21	116	7.74	5	18
29	120	6.15	5.1	20
26	124	6.09	5.05	17
26	150	6.41	7.1	20
27	105	6.12	5	22
26	142	6.12	5.2	22.5
25	110	6.75	5	18
22	120	6.66	5.8	20.5
32	126	7.15	5.2	20.5

21	128	7.36	5.3	22.5
27	135	6.41	5.4	17
22	125	6.5	4.9	18
22	104	7.06	6.7	17
30	125	5.62	6.05	22.5
26	120	6.22	4.95	23
25	110	7.12	5.5	16.5
32		5.37	5.2	14
27		5.55	5.6	22
31	125	5.02	5.7	18
33	150	5.9	6.6	22
34	120	6.19	5	20.5
27	125	6.34	5.24	19
23	120	5	5.1	17
22	170	7.69	4	21
46	178	4.96	8.2	21
31	130	5.96	5.65	20
23	120	6.69	5.6	18
27	115	5.78	5	20
29	135	6.22	6.5	17
25	130	6.09	4.1	17.5
16	104	6.84	4.1	17
35	146	6.26	6	20
22	195	7.81	4.2	18
	105	7.81	5	18
27		5.59	5.3	23
20	110	7.51	3.5	23
23	115	6.38	5.6	20
28	135	7.23	4.2	14
34	127	5.75	5.5	17
27	105	6.99	5.2	13.5
28	130	6.71	3.6	20.5
30	125	5.78	4.75	25
29	128	6.34	5	22
20	115	7.63	5.3	19
20	105	7.29	5	17
20	92	7.71	4	17
30	153	5.74	6	17
27	138	5.76	6	24
26	117	6.62	4.7	20.5
26	135	6.86	4.8	19
22	123	7.04	5	19
32	160	5.07	5.5	18
20	115	7.07	4.1	
25	160	5.02	4.5	19
35	170	5.28	5.45	20.5
20	130	5.77	6.7	17
	225	6.12	4.8	22
25	125	6.59	5.9	21
23	125	6.31	4.6	20
30	170	6.3	5.6	21
42	150	5.31	5.2	22
34	140	6.36	5.6	20

27	220	5.25	6	23
22	140	5.3	4.5	19
32	145	5.2	6	23.5
30	170	5.36	5	23
25	160	6.85	6.8	19
30	140	5.95	6.7	21
	170	5.36	6.2	22.5
21	135	7.21	4.3	19.5
28	160	5.31	5.3	22.5
29	160	5.43	8.3	21.5
23	103	5.45	5	19.5
21	120	6.94	3.3	21
27	220	5.38	6.6	21.5
25	160	5.63	5.8	21
21	130	6	5.8	20
25	130	6.53	3.6	19
28	160	7.31	5.5	17.5
22	150	7.1	4	17.5
29	150	6.31	6.3	19.5
30	160	6.02	6.6	19.5
35	150	6.37	5.8	20
23	150	5.71	6.4	21
30	160	5.87	5.6	20.5
24	120	5.91	6.5	21
30	160	5.64	6.6	21
31	160	6.48	4.3	19
22	150	5.63	4.5	19.5
30	150	7	6.7	18.5
20	150	5.92	5.4	18.5
35	160	5.48	6.3	21
20	150	6.41	5.7	18.5
	140	7.28	4.7	18
	100	7.35	5.1	18.5
	152	6.19	4.8	21
	150	5.45	5.9	22
	122	6.31	4.2	19
	155	6.44	4.7	22
	130	5.48	5.1	22
	98	5.81	4.4	20
	160	5.63	4.7	21
	110	5.41	5.5	22
	110	6.35	5.9	21
	220	6.95	5.2	21
	140	5.63	5	21
	210	5.8	5.9	23
	130	5.31	5	21
	120	5.35	4.7	21
	170	5.71	5.7	22
	110	6.98	5.8	19
	155	5.58	4.1	22
	120	5.91	4.7	21
	103	5.58	4.7	20
	170	5.32	5.6	21.5

	160	6.18	4.2	19.5
28	135	6.8	3.8	18
20	120	6.55	4.7	20.5
26	160	5.01	6.5	21
23	160	6.43	5	14
30	145	5.81	5	21
21	135	6.04	5.2	17
25	160	5.8	4.2	19
17	140	5.8	6	19
17	115		5.7	
30	140	6.19	4.7	23
30	125	6.8	5	18
27	115	5.87	4.9	15
26	130	5.25	4.3	19.5
20	145		5.8	
25	170	5.94	5.5	21.5
20	120	6.25	5.4	16
23	135	5.79	5.6	22.5
25	165	5.79	6.5	23
25	170	5.41	4.9	23.5
37	175	5.71	6.6	20
29	168	5.38	5.8	18
25	160	4.85	4.7	19.5
21	150	5.72	5.1	18
32	145	6.47	4.4	18
30	168	5.48	5	21
30	165	5.8	5.5	21
20	130	6.18	4.1	18
23	165	5.9	4.5	21
25	150	6.12	4.5	20
25	165	5.4	4.5	21
30	165	5.84	5.5	20
35	170	6.1	4	20
30	160	5.3	5.6	21
26	132	5.23	4.3	19
23	140	5.71	5.6	18
28	125		5.8	
27	140	5.45	4.6	17
24	140	5.72	5.9	20.25
25	170	5.3	5.2	21
37	185	5.9	5.8	18
29	170	6.38	6	18
27	160	6.36	5.9	19
20	160	5.72	5.8	19
29	120	5.36	4.7	20
25	170	5.85	6.7	18
26	120	7	5.8	17
30	175	5.4	5.2	19
27	140	6.1	5.5	18
20	135	7.1	5.8	17
30	145	5.9	5.5	18
13	160	5.9	6.5	18
25	160	6.4	5.2	18

35	145	5.8	5.6	19
30	195	5.8	5	20
27	165	6.33	4.9	18
28	135	6.2	5.5	18
30	170	5.9	5	19
25	140	5.22	5.1	20
25	160	6.8	6	18
17	90	7.18	5.5	
22	115	7.7	3.6	
20	115	6.1	6.2	20
22	100		4.5	
20	130	5.9	6.7	18
18	118		4.2	
33	160	5.17	6.7	24
28	157	5.8	5.1	15.5
19	119	7.5	4.8	19.5
27	163	5.84	5.1	22
24	113	6.93	4.9	17.5
27	105	7.8	4.9	20
24	125	5.78	5.1	20
27	140	5.07	4.2	20.5
25	120	5.97	5	18.5
30	140	6.38	5	16.5
20	160	5.39	4..5	22.5
30	160	6.84	6	17.5
26	155	6.15	5.94	22.5
30	137	5.9	4.24	20
22	117	7.5	4.56	16.25
26	161	6.5	5.5	16.75
29	161	5.71	5.6	12
29	147	6.12	4	19
25	135	6.89		21
38	160	6.07	7.5	25
33	165	5.31	5.25	25
27	130	6.27	6.09	25
25	168	5.68	5.65	25
32	190	5.75	6.46	27.5
34	173	5.54	5.88	31
32	180	6.35	5	24
25	139	6.15	5	14
24	134	6.34	4.6	16
27	123	6.93	4.35	16.5
23	137	5.96	4.8	12
33	159	6.06	5.28	20.5
30	150	6.06	4.8	20
29	185	5.25	6	18.5
23	142	5.3	5.28	20.5
38	147	6.71	5	16
24	155			
20	135		5.8	16
17	150	5.41		20
24	135	4.97	4.98	11.5
26	164	5.21	6.1	17.75

25	107	6.15	4.1	20
30	158	5.93	5.1	17.5
26	157	5.4	4.74	20.5
36	161	6.21	6.3	20
36	161	5.31	6.2	21
31	152	6.93	5.95	7
29	123	6.59	4.7	18
26	150	6	5	14
27	125	6.25	5.1	18
20	132	6.09	6.4	14
34	146	6.2	3.14	18
20	125	7.18	4.43	19
27	137	5.93	4.31	18
14	115	7.87	4.32	14.5
24	157	6.18	5.2	21
18	112	7.37	5.55	18
29	148	5.78	5.81	18.5
30	170	6.31	5	22
35	150	6.26	6	19.5
22	138	6.12	4.1	16
25	146	6.85	5.17	19
34	130	6.2	4.85	21
40	150	6.6	5.56	17.5
28	120	6.37	5.3	19.5
26	103	6.6	4.84	17.5
20	128	6.75	4.5	14.5
26	156	6.81	4.6	17.5
25	120	6.21	4.01	21
29	148	6.34	4.9	17.5
31	112	7	4.6	12
30	170	6.68	5.1	19
26	130	6.72	4.8	19
28	140	5.98	5.78	22
23	128	6.5	5	19
30	100	7.15	5.7	16
25	169	5.62	6.3	20
30	130	6.38	5.9	10
26	129	6.4	3.95	18
25	147	7	4	21.5
30	156	5.52	2.5	16.5
30	160	5.31	5.1	21
25	90	6.47	6.7	14.5
24	150	6.7	5.4	17
27	140	7.02	5.4	14.5
22	160	7	4.6	16.5
20	100	6.97	4	21.5
19	110	7	3.66	11
28	130	7.3	4.3	21.5
28	140	6.9	5	18.5
30	140	6.31	4.05	20
20	120	6.87	4.95	20
26	165	6.25	4.2	19.5
25	145	6.15	4.95	18.5

27	170	5.7	4.96	22
25	145	6.16	5.78	22
22	150	6.7	4.8	20.5
31	130	6.5	3.67	22
30	155	6.5	5	20.5
23	170	6.5	5	22
25	165	6.99		19
23	155	7.1		19.5
24	160	6.88	5	22
20	150	7.12	5	21
23	160	6.5	4	19
27	148	7.01	4.92	21
24	159	5.78	4.83	18
25	150	6.32	6.27	22
22	140	6.75	4.95	18.5
26	165	7.22	4.1	16
22	170	6.69	3.05	16.5
20	125	7.1	4	18
40	170	5.19	6	21.5
28	150	6.54	6	21.5
30	170	6.2	5.35	22
25	140	6.17	5.5	18.5
30	140	6.03	5	18.5
37	155	6.19	5.56	19.5
27	175	6	5	21.5
22	170	6.1	5.7	22
26	170	6.66	5.05	18
27	170	6.3	5	22
30	184	5.7	5.7	22
30	180	6.66	5.4	15
32	170	5.84	5.55	19.25
19	185	7.65	4.24	18
23	187	5.86	5.87	20
25	190	6.25	5.8	19.5
30	180	6.29	5	21
30	170	6.53	5.7	19.5
34	177	5.96	5.6	21
31	172	6.02	4.8	17.5
24	165	6.75	4.9	20.5
24	135	7.08	4.7	
22	130	7	4.1	21.5
30	149	5.67	6	19
	135	5.59	5	21
22	160	5.85	5.77	15
24	112	5.47	5.13	19
30	150	5.89	4.33	19
24	122	6.14	4.74	18
24	159	6.31	4.73	17
24	174	5.97	6	22
25	165	6.04	4.9	20.5
27	140	6.23	7.3	19
29	160	5.93	7.8	15.5
38	161	5.48	6.75	21

28	135	6.71	6.7	16
24	125	6.43	6.5	19
24	145	6.4	5	12
26	180	6.04	5.87	17.75
25	160	5.18	4.85	18
25	155	5.85	4.72	17
22	140	6.31	5	17
29	140	6.22	5.45	20
23	110	7.03	4	16
28	180	5.58	5.5	14
23	150	6.47	4	10
26	160	5.93	5.95	12
18	110	7.78	2.63	14
10	150	6.4	3	19.5
11	80	8.44	4.35	14.25
28	140	7.25	5.54	16
22	130	6.63	4.59	15
15	150	5.9	5.8	16.5
24	170	6.64	3.42	17.75
26	150	7.53	5.23	14
18	120	7.31	4.74	13.75
22	140	6.84	4.4	17.75
16	105	8.34	3.63	12
23	180	5.95	5.74	15
22	160	6.21	4.79	12.75
17	150	6.96	4.1	15.5
28	165	5.87	4.07	16.75
20	150	6.31	4.9	16.75
23	150	5.84	5.2	14.5
29	190	5.53	5.15	13
29	179	6.53	4.5	16.5
29	165	5.1	5.2	17.5
22	135	7.68	6	18
19	130	7.72	4.5	14.5
29	164	5.72	4.3	17
29	140	6.91	4.8	14.5
27	155	6.72	6	15
16	120	6.72	5.8	14.5
22	130	6.22	5.8	15
21	132	7.03	5.3	19
27	155	5.6	6	19
21	155	6.22	5.8	18
24	150	7.54	5.2	14.5
27	131	6.18	3.7	18
25	130	7.09	4.43	15
37	155	5.96	4.67	19
37	160	5.96	5.5	20.5
26	150	5.84	4.37	16.75
27	140	6	3.75	15
22	150	7.09	6.1	17
32	160	5.71	5	17
27	130	7	5.45	16
25	140	6.75	5.25	18

26	160	6.21	5.2	18
25	130	6.64	4.97	14
25	140	7.46	4.98	17
30	140	7.25	4.74	16
25	140	5.75	4.52	18
40	150	6.5	5.1	17
22	150	5.75	4.32	19
27	130	6.84	4.75	19
24	140	6.93	4.8	15.5
26	170	6.36	4.82	17
28	160	6.25	5.2	15.5
28	140	7..16	6	13
24	120	6.97	5	16.5
25	119	7.66	4.5	16
24	132	8.32	6.2	12.5
23	130	6.59	5.3	13
27	150	6.6	5.2	17.5
25	140	6.65	5.2	15
24	120	6.97	5.5	14
32	168	7.34	6.55	16
30	180	6.07	5.73	17.5
31	138	6.56	5	17.5
30	171	5.97	5.9	16
23	160	6.07	5.94	18.5
25	150	6.4	5.3	14
27	150	5.85	5.7	17.5
25	128	7.34	3.9	12
27	130	7.53	5.35	14.5
26	140	6.69	6.19	16.5
17	110	9.06	5.3	10
21	120	7.47	4.34	15
24	135	6.84	4.88	14.5
22	131	7.88	4	
24	150	6.86	4.52	14.5
20	101	6.29	5	11
25	140	6.31	4.36	12
23	146	6.28	5.25	13
30	113	6.16	5.35	26
20	121	6.5	5.12	21
31	172	5.84	6	25.5
26	142	7.52	5.33	17.5
26	158	5.3	5.41	21
27	124	6.77	5	17
28	152	6	6	20
27	138	6.5	6.5	22
26	149	6.25	4.58	21.5
15	120	8.64	4.9	19
29	110	8.37	4.9	14
31	120	6.75	5.4	17
24	150	6.5	5.8	17
35	180	5.5	7.45	23.5
35	165	6.16	6.28	19
30	148	6.91	6.4	22

	120		5.18	10
30	159	6	4.3	20.75
25	140	6.46	6.86	18
30	180	5.93	6	21
35	147	5.38	6	24
30	179	6.25	6	19
35	162	6.19	6.43	
30	130	6.96	4.7	
30	150	6.63	4.93	16.5
31	170	5.89	5.89	19
18	100	7.47	5.76	18
22	150	6.85	5	20
33	145	5.9	5.7	20
25	170	6.44	5.98	21
45	141	6.53	7.14	21.25
25	142	6.38	4.59	23
35	168	5.75	5.51	23
25	181	5.97	6.61	20.75
30	148	6.3	5.8	26.5
26	130	7.31	5.28	19
35	170	6.25	6	16
25	149	6.88	6.1	21.5
32	151	6.84	5.3	18
15	111	8.29	5	12
18	140	7.81	5	15
30	190	7.6	5.51	16.5
25	170	6.63	5.3	15.527
28	179	6	5.65	14
25	123	7.56	5	16
35	150	5.53	5.58	22
21	120		5.11	
30	167	5.91	6.2	16.5
29	110	7.41	4.9	17
28	180	5.81	7.5	22.75
28	150	5.69	6	22.5
26	130	7.12	4.94	17
30	150	5.97	7	14
24	144	6.16	5	16.25
36	150	5.85	6.65	19
35	140	5.62	5.31	21.5
35	169	5.78	6	20
39	172	5.19	7.39	26.5
30	142	6.37	5.46	20
35	180	5.61	5.32	20
25	158	6.84	5.85	21.5
25	147	6.13	4.44	20.5
26	160	5.39	5.51	22
30	170	6.6	7.42	15
24	128	7.31	5.7	21.5
38	158	6.53	6.8	21.5
45	180	5.31	8.1	25
34	158	6.25	5.65	23
34	158	6.82	6.4	21.5

34	150	6.35	5.5	18.5
35	170	6.19	6.34	14
28	160	6.06	6	22
27	142	6.88	5	20.5
26	159	6.31	5	18.5
30	135	6.09	4.68	16.5
34	150	6.34	5	17.5
32	155	6.1	4.68	21.5
41	136	6.32	4.5	20.5
27	135	7.53	6	17.5
23	129	7.44	4.3	12
35	180	5.13	5.63	24
25	158	6.13	5.62	15.5
35	114	5.88	6	21.5
35	140	6.97	5.1	28.5
31	135	6.82	5.7	21.5
36	140	6.55	5	20.5
34	148	6.69	4.55	20.5
28	143	7.12	6.8	15.5
35	170	5.78	7.25	25
30	150	5.57	5.5	20
30	183	6.53	5.63	19
25	100	8.12	4.47	17
20	140	7.1	4.39	21
20	120	7.62	6	21
35	160	6.82	4	24
37	165	5.96	7.6	23
28	144	6.37	6.1	21.75
26	130	6.84	5.25	18
29	165	6.49	5	21
20	170	6.35	5.45	16.5
25	132	6.31	4	19
25	130	6.3	5.2	13
24	148	6.72	5.25	22.25
30	170	6.26	6	20
25	105	7.3	4	17
23	140	7.1	6.85	17.75
30	140	6.91	4.59	22.25
17	130	7.31	6	15
	185	5.69	7	21
	190	5.2	7.5	23.5
22	118	6.28	4.3	17
17	100	5.58	5.59	18
23	131	5.8	4.75	20
34	132	6.19	5	17
10	80	7.41	5.6	15
20	140	6.03	5.9	17
23	105	7.69	5.3	13
32	127	5.8	5.77	19.5
21	132	5.39	5.6	22
27	105	6.15	5.7	20.5
29	125	5.9	6.07	19.5
34	157	5.57	6.7	21

27	125	5.91	6.77	20.5
25	123	6.1	6.29	16
30	122	6.5	6.62	16
29	120	5.9	5.9	21
34	120	6.04	5.07	18
17	110	6.4	5.29	16
28	140	6.3	7.66	18
23	140	5.6	5.27	20
17	125	5.39	5.5	20
27	130	5.55	5.7	21
26	142	5.72	5.44	21
38	150	5	5.27	27.5
29	149	5.2	5	20
24	125	5.41	5.3	22
25	120	5.32	4.86	14
27	110	6.4	6.53	19
26	151	5.21	5.85	22
53	125	5.39	6	13
45	113	5.73	5	17
19	120	6.01	5.7	19
20	135	5.2	5.43	17
19	124	6.59	4.44	20
30	127	5.85	5.7	12
19	115	6.93	5	12
27	105	6.16	6.4	16
29	115	5.3	5.7	23.5
26	122	6.5	6.07	14
19	100	6.89	5.9	17
20	125	6.1	5.68	20
30	147	6.03	5.75	18
27	140	4.85	6.32	24
29	155	4.49	5.5	21
38	130	4.67	6.53	20
21	105	5.89	5.2	14
55	150	5.34	6	21
23	157	5.63	4.82	20.5
27	101	6.1	5.47	7
33	132	6.18	5.9	17
34	134	5.32	6.18	19
29	123	5.9	6.7	11
23	129	6.5	6	16
20	119	6.59	5.52	17.5
25	115	6.66	4.3	16
29	148	6.18	5.52	17
31	132	5.81	6.8	17
24	145	5.73	6.3	18
19	115	6.01	5.35	16
21	103	6.41	4.83	14
29	129	5.47	5.3	17
23	101	7	4.47	15
23	105	6.01	6.5	20
29	121	6.15	4.72	7
29	133	5.45	5.1	20

35	135	5.44	5.12	19.5
50	140	6.12	5.75	19
20	120	7.3	4.47	17
32	115	6.25	5	21
25	150	6.3	5	17
32	120	5.9	5.1	22
29	138	7.5	6	19.5
15	97	6.62	5.1	16.5
24	118	6.18	6.1	17
24	130	5.9	4.9	17
14	110	6.1	5.6	16.5
20	117	5.9	5.35	19.5
38	171	5.05	6	20.5
29	110	6.4	6.1	18
25	115	5.93	5.8	19.5
28	172	5.09	6.9	15
24	114	6.78	6	7
20	142	5	5.1	19
23	141	6.09	5.2	19
23	112	6.21	4.91	19
23	137	5.61	6.51	17
27	117	5.41	4.7	16
20	135	5.31	5.62	23
21	81	7.01	5.25	14
15	127	5.21	4.86	15
48	122	5.57	4.78	19
24	109	6.25	5.07	18
27	131	5.75	6.48	18
31	120	6.41	4.81	13
25	131	5.5	6.8	18
27	124	5.56	8.26	10
23	118	6.09	6.2	16
24	125	6.09	5.44	14
22	143	6.31	6.4	18
28	123	5.1	5.1	27
27	126	5.6	5.7	19
18	130	6.01	5.91	19
20	115	6.34	5.7	18.5
23	117	6.22	5.81	19.75
26	122	5.8	5.9	20
19	110	5.8	4.25	17.5
26	131	6.28	5.9	16
19	80	5.94	5.1	
25	132	5.4	5	22
35	115	4.6	7	21.5
21	137	5.66	5.78	19
25	147	5.25	6.34	19
	100	7.8	5.2	15.5
29	124	5.81	4.8	19
29	165	5.06	5.7	25
30	160	5.03	5.5	23.5
29	140	5.7	4.75	21
25	120	6.93	3.9	19.25

31	165	5.9	5.1	18
16	110	7.12	4.46	14.5
19	120	6.28	4.35	11
27	140	6.15	5.3	20.5
28	130	6.37	6.1	19
18	129	5.8	6.02	14
23	120	5.5	5.68	20
23	170	6.37	4.89	21.5
40	135	6.03	7.1	18
23	140	6.62	4.7	20
18	110	6.79	5.03	18
15	103	6.44	4.5	17
17	110	6.83	8.5	12
24	130	6.3	4.63	15
28	150	5.1	5.43	18
31	150	5.4	6.03	15
28	120	5.46	5.7	19
27	150	5.1	5.96	18
19	132	6.15	6.3	19
24	112	6.12	4.63	19
25	142	5.73	6.45	21
30	100	6.47	4.9	18
27	110	7.56	4.3	10
24	127	6.29	5.6	18
22	99	6.9	5	12
32	133	5.91	5.6	15.5
20	123	6.16	6	19
36	152	5.03	5.1	23.5
27	119	5.66	6	19.5
25	107	5.6	4.3	25.5
39	135	5.07	6.8	21.5
21	108	7.06	5.63	11
24	130	6.35	5	16
18	122	6.04	4.27	22.5
21	111	5.88	5.37	23.5
41	122	5.06	6.5	21
31	119	6.09	6.25	19
32	108	5.65	5.5	21
17	128	5.66	6.1	21.5
33	97	5.84	5.35	18.5
30	115	7.02	5.8	19
28	118	5.94	5.1	20
27	110	6.06	5.5	21
22	99	6.72	5	21.5
37	132	5.47	7.1	21
31	103	5.73	6.35	21.5
29	104	5.78	6.9	18.5
20	90	6.84	4.1	17.5
35	100	6.08	4.85	16
35	113	5.98	5.8	16.5
36	146	5.37	6.43	21
44	117		6	
32	98	6.94	5.1	18.5

31	127	5.88	5.45	9
36	140	5.91	8.3	10
27	98	6.22	4	18
34	139	5.81	6.55	19
31	121	6.09	5.01	17.5
40	130	5.37	7.4	20
39	152	5.44	6.45	19.5
44	133	5.41	5.8	12
29	91	6.87	4.8	16
28	106	5.31	5.6	15
27	118	6.22	5.8	16
29	117	6.22	5	17.5
27	119	5.78	5.1	20.5
31	110	6.35	5.7	18.5
23	101	6.12	4.8	20.5
26	101	6.9	4	21.5
36	122	6.5	5	17
21	80	7.22	3.9	18
33	130	5.37	6.1	19.5
25	129	6.44	5.11	21
29	102	7.02	5	19
34	108	5.19	5.4	24
32	114	6.22	6.15	20
24	84	6.68	4.6	17
28	96	6.25	5.5	20.5
29	126	5.53	5.5	19
32	132	5.78	6.2	17
32	105	6	4.8	22.5
27	89	6.22	4.5	17
25	130	5.44	4.6	20
27	107	5.94	4.11	17
27	97	6.5	4.5	19.5
33	93	5.44	5.4	20
24	115	6.09	6.1	19
44	138	5.4	6.15	21
24	93	6.35	5.3	15
37	133	5.65	5	20
31	120	7.09	5.9	16
31	90	7.16	5.59	17
36	100	7.06	4.2	20.5
44	145	5.2	5.75	21.5
34	126	5.81	5.35	18
28	107	6.22	5.54	20
30	103	5.91	6.32	17
33	103	5.81	5	20
27	91	6.32	4.9	18.5
28	122	6.47	4.6	23.5
14	105	6.53	4	17
34	140	5.53	5	22.5
32	121	6	5	19.75
25	103	6.31	5.3	19.5
30	122	5.75	5.35	19.25
27	117	9.31	4.5	17.5

33	112	6.53	4.5	22.5
29	140	5.81	5	23.5
29	123	6.62	7.25	16
33	119	6.01	7.1	20.75
24	118	5.94	6.64	22
38	108	5.8	4.9	18.5
32	119	5.54	6	19.5
25	99	5.9	5.22	17
37	144	5.22	6.9	20
29	91	6.03	5.2	17
27	110	6.37	5.7	19.5
24	91	6.69	4.6	16.5
31	109	5.81	5.4	19
42	108	6.25	5.55	16
37	129	5.6	6.65	18
28	113	6.34	4.45	17
42	128	5.47	5.93	19.5
29	111	6.19	6.44	17
30	94	6.75	5.5	16.5
34	108	5.9	6.1	17
26	101	8.19	5.6	12
36	119	7.34	5.27	15
28	83	6.03	4.28	13
30	123	7.57	4.51	20
30	104	7.08	5.38	21
32	117	6.95	4.54	20
45	130	6.54	6.1	21
40	126	6.64	5.81	24
32	105	6.74	5.16	20.75
21	83	7.09	4.4	14.75
19	82	7.16	4.12	12
28	111	6.7	5.47	22.5
39	117	6.89	5	20.5
25	88	7.75	4.86	17.5
44	119	7.18	5	20.5
33	112	7.41	4.91	22.5
27	94	9.33	5.1	17
47	161	6.44	5.3	24
30	108	6.44	5	21.5
26	98	7.85	4.45	19.5
44	137	8.29	5	17.5
28	104	7.79	5.13	17
24	92	8.12	3.51	20.5
37	129	6.36	3.74	17.5
21	86	6.07	5.82	17
26	115	6.37	4.48	16
33	118	5.59	5.7	19
22	102	5.53	4.8	24
21	92	7.78	4.65	14
30	137	6.62	4.6	18
24	88	5.5	5	19
25	110	6	4.85	18
20	109	6.53	5.73	21

29	113	5.65	7.48	21
36	104	5.31	6.1	22
35	152	4.73		25
27	109	6.08	6.8	18
28	124	5.93	5	20.5
20	122	6.89	4.19	21.5
21	135	6.17	4.43	16.5
22	101	6.59	5.48	18
32	146	6.94	5.19	21.5
26	135	7.03	5.18	20.5
24	145	7.37	4.8	20
13	120	8.07	5.23	21.5
32	150	6.22	4.93	17.5
22	121	6.53	4.29	20.5
28	110	5.35	6.11	24.5
30	137	5.8	4.87	19
49	142	5.59	8.5	25.5
28	96	6.47	3.9	17.5
34	113	8.03	4.31	
31	81	6.53	5.2	16.5
26	102		4	18.5
29	140	6.44	4.8	17.5
33	141	5.59	6.4	20
31	113	6.75	6.5	15.5
38	161	6.09	6.8	17.5
30	143	5.96	5.7	21.5
26	134	6.07	6.24	20.5
23	106	6.17	5.6	19.5
40	152	6.8	5.72	18
35		5.97	5	19
40	144		5.87	
40	145	5.54	4.9	
39	162	5.31	5.9	20.5
37	162	7.25	7.3	19.5
39	145	6.3	5.6	17
	171	6.06	5.8	24.5
37	170	6.16	6	18
30			5.38	
45	165	6.45	5.8	17.5
27	170	6.02	6.5	16.5
28	160	5.3	5.8	21.5
27	170	6.36	5	19.5
32	130	7.25	7	
30	135	5.04	4.8	26.5
24	100	7	4.95	20
35	130	6.29	6.4	22
22	135	5.07	5.3	20
33	135	5.72	5.05	18
23	115	5.53	5.1	20
31	118	5.49	4.5	22
25	115	5.97	5	23
18	95	7.41	4.7	16
17	90	7.29	4	16

33	120	6.09	5.1	21
30	164	6.04	5.95	22
32	130	5	6	18
25	110		4.5	
25	105	7.12	6.25	19
23	120	6.09	4.7	17
25	115	6.03	4.95	19
25	135	8.01	5.65	17
24	122	6.53	5.2	16
29	121	6.71	6	20
42	150	5.07	6.7	23.5
30	120	6.13	5.9	15.5
25	122	6.03	5	19
25	121	6.13	6	16
31	125	6.5	4.7	19
30	115	6.77	5	19
20	115	7.19	4	16
33	140	6.09	6	20.5
33	125	6.02	6.55	19
39	160	5.06	8.3	21.5
35	130	5.78	6.05	17
31	130	6	4.95	24
16	100	9.06	2.5	7
35	135	6.17	4.95	16.5
25	135	6.04	5.2	18.5
31	125	6.46	5.95	22
33	155	6.04	6.1	18.5
20	110	8.11	4.2	19
22	105	6.92	4.95	19.5
21	120	6.42	4.8	23.5
25	120	6.97	5.65	18.5
33	130	5.05	5.75	24
29	120	5.04	4.85	19
34	120	7.22	4.8	18
38	145	5.78	6	20.5
32	230	6.72	4.2	17
21	110	8.19	5.2	19
24	105	6.72	4.65	16
31	125	6.22	5.1	22
25	130	5.01	7.5	21.5
33	130	5	6.85	16.5
24	120	5.78	5.2	22.5
31	110	6.8	6.65	20
25	250	5.45	6.9	22
25	200	7.04	5	15
20	195	8.16	5.1	15.5
20	205	9.74	4.1	12
25	235	8.25	4.8	18
36	220	7.75	5	15
23	145	5.62	5.1	21.5
20	130	6.94	4.8	20
25	120	6	4	19
20	105	6.21	4.56	19

25			5.8	
36	124	6.47	4.9	23
	100	6.94	5.75	22
30	105	5.72	5.25	22
29	150	5	5.95	21
22	118	6.44	5.3	17.5
26	100	8.1	5.3	19
27	120	6.02	5.35	16.5
25	125	5.04	5.05	22
32	157	6.06	6	24
26	120	7.9	5.5	21.5
27	120	7.71	4.95	19
26	135	5.98	8.4	22
34	128	5.87	5.1	20
30	134	5.58	8.5	22.5
43	160	5.19	8.5	22
20	145	5.7	5.1	22
27	130	6.25	4.5	17
20	165	7.06	4.7	22.5
26	132	6.07	4	18.5
28	178	5.75	5.9	26
26	148	6	4.9	21.5
22	148	5.8	4.5	21.5
24	132	6	5.3	20
12	103	7.15	4.15	16.5
30	149	5.56	5.6	21.5
30	133	5.59	5	22
24	140	5.65	5.45	22
27	152	5.59	6.4	22
26	117	6.43	5.1	19
21	127	6.16	5.8	17
27	127	5.97	5.1	20
31	132	5.41	5.5	23
39	195	5	6	23
33	147	6.06	4.6	22
33	148	5.78	6	22
30	160	5.59	5.2	21
32	149	5.91	5.4	19
23	149	6.44	4.95	19.5
15	100	7.78	4.3	14.5
32	172	6.5	4.9	19.5
34	185	5.72	5.45	21
25	142	5.63	5	23
35	160	6.44	6	20
21	148	5.78	4.9	17
24	120	6.47	5.45	16.5
25	117	6.18	5.4	22
31	135	6	5.45	20
32	166	5.41	5.4	24
27	148	6.19	4.5	21.5
30	165	5.59	4.95	20.5
30	149	5.69	6	20.5
31	149	5.78	6	19

19		6.6	4	17
25	122	6.31	6	19
33	150	6	5.9	19
22	122	6.34	5.2	19.5
29	164	5.25	6	17.5
28	146	6.37	5.85	
28	139	5.94	5.7	20.5
26	135	5.82	6.5	22
25		6.63	4.2	19
25	107	6.75	4.45	20
17	122	6.21	3.6	20
27		5.87	4.4	
26	132	6.09	5.6	
	130	6.53	4.1	
21	124	6	4.7	
13	106		4.5	
	167	5.71	6.2	23
	152	5.81	5.5	
30		5.82	6	20.5
	147	6.03	5.9	
30	127	6.28	4.3	
	147	6.21	4.6	18
	143	6.41	5.6	
	120	7	5.15	
27	128	6.87	4.85	
33	149	6.1	4.45	8
33	145	6.31	4.95	8
29	130	6.21	5.64	15
23	120	7.47	3.63	
25	120	7.1	4.63	8
26	138	6.38	5.53	11
32	110	6.81	5.51	
36	148	6.25	4.94	
32	165	5.87	4.9	
42	132	6.16	4.9	8
39	138	6.66	5.63	
28	120	6.94	5.78	8
28	118	7.19	4	14
30	105	6.97	5.26	7
33	130	7.28	4.27	7
31	181	5.88	5.98	13
30	135	6.56	5.62	9
33	140	6.97	5.05	8
28	115	7.6	4.73	
27	112	6.75	5.1	
28	115	7.41	5.05	
34	140	6.69	5.27	23
31	151	5.85	5.78	23
26	126	6.72	6.63	
33	129	6.53	4.88	
28	120	6.75	5.58	11
31	140	6.38	5.63	18
22	132	7.09	4.66	9

34	152	5.78	5.42	14
27	110	6.73	4.58	
35	162	5.85	6.06	20
29	140	6.34	5.05	
31	130	6.41	5.1	17.5
29	132	6	4.85	19.5
37	110	6.37	5.98	20
33	130	6.5	4.4	20
45	151	5.66	5.81	20
36	140	5.88	5.79	20
25	120	6.53	4.9	9
35	161	5.53	7.24	27
25	100	6.47		8
35	125	6.22	7.16	
27	115	6.25	4.84	10
29	134	6.78	4.7	8
	151	5.85	5.4	
24	102	7.18	4.05	8
22	122			
24	120			
23	130	6.41	5.2	
20	125	6.34	5	
22	128	6.91	5.2	14
26	142	5.8	4.5	9
22	139	6.44	5.15	
23	100	6.66	4.95	
22	109	8.16	5.35	
26	130	5.85	4.95	13
26	122	6.41	5.7	18
26	127	6.06	5.1	19
31	129	5.84	5.1	7
30	150	5.62	5.4	14
23	125	6.47	6.4	
29	125	6.53	5.6	
32	120	6.03	5.1	16
31	135	6.21	6	15
28	118	5.19	4.75	18
23	95	8.06	4.75	
28	129	8.25	4.85	19
28	122	6.59	6.8	
34	134	6.09	5.5	20
27	145	6.47	4.6	
28	116	8.91	5.5	
30	130	6.44	5.2	
33	139	6.12	5.4	19.5
38	148	5.63	4.75	19.5
33	122	6.09	5	
30	115	6.29	5.6	
22	112	7.03	3.8	
25	130	6.28	5.65	
23	120	7.13	5.4	
21	141	5.91	5.25	9
22	110		5.4	

23	110			
31	124		5	
28	145		5.55	
30	148			
24	133	6.3	4.6	19
20	124	6.96	5.15	14
24	133	6.37	5.3	22
23	132	6.93	4.1	18
23	163	7.7	5.6	23
33	152	7.1	5.2	23
33	140	6.9	4.4	20
20	153	5.9	5.7	22
26	144	6.35	5.2	16
35	191	5.95	7.2	17
30	135	7.1	7.1	16
30	145	6.85	5.2	15
20	122	6.9	5.1	21
20	128	6.87	6	23
27	152	6.71	6.5	25
28	162	6.08	5.9	22
25	154	6.09	5.7	23
21	134	7.2	5.15	28
23	133	7.06	4.3	22
14	131	6.9	4.8	19
34	143	7.26	5.1	17
17	124	7.9	3.8	14
32	160	7.16	4.8	26
20	139	7.39	4.1	21
19	115		4.4	19
23	100	9.23	3.7	24
11	106	8.87	4.9	21
19	115	8.06	5	23
13	132	7.4	5.06	23
21	121	6.43	4.4	21.5
16	140	6.35	3.75	15.5
19	120	5.17	6.23	16
18	170	5.51	5	19.5
21	132	6.5	4.5	18.5
17	145	6.89	4.6	16.5
10	128	7.1	5.8	19.5
17	124	6.39	6.1	23.5
14	107	6.39	4.8	19.5
14	110	6.47	4.6	18.5
16	138	5.96	5.3	18.5
18	111	6.08	3.7	20
20	143	6.03	5.2	22
22	132	5.33	4.9	24.5
22	160	5.69	5.3	21.5
23	180	5.37	4.69	25.5
13	115	6.3	4	19
10	142	6.46	3.8	16
12	130	6.9	3.45	14.5
24	128	6.4	3.4	19

12	140	6.1	6	16
22	139	6.14	5.02	16.5
21	149	5.89	6.43	18
23	153	5.38	6	18
28	157	5.07	5.44	19
27	139	5.6	4.41	16.5
22	143	5.7	4	19.5
25	160	5.69	5.1	19
27	123	6.01	4	18
17	127	7.63	4	18.5
25	141	5.93	6	19
21	130	6.79	5.61	17.5
13	124	6.41	4.19	19
18	132	6.49	5.15	18
20	167	5.39	6.77	21
17	119	6.45	5.42	16
22	151	5.84	5.55	18
15	115	6.2	4.43	18.5
12	145	6.38	5.47	16
20	168	5.49	6.01	19
22	127	7.05	6.22	17.5
30	190	5.17	6.6	23.5
30	110		5	
30	150	4.85	6.5	21.5
35	150	6.13	6	
25	140	5.5	5.5	
40	180	5.85	6.8	20
37	165	5.46	7	22
40	162	6.58	5.5	16
25		7.19	5.1	16.5
30	130			14.5
25	110	6.71	5	21
22	170		5.5	
25	100	6.45	4.5	13
25	123	5.7	4	14.5
30	130	5.18	5.5	18
30	125	5.62	6	14
30	135	5.3	6.5	15.5
30	134	5.5	6	19.5
20	165	6	4.1	
55	180	5.71	6.7	23
25	120	7.26	4.6	20
35	130	5.62	6.5	23.5
40	130	5.2	6.5	20.5
25	129	5.18	6.5	19.5
25	125	5.28	7.5	25
25	145	5.3	6.5	22
30	133	5.18	6.5	23.5
35	120	6	5.5	13
25	100	5.52	6.5	18.5
30	160	5.38	6.5	15
25	135	5.18	5.5	18.5
35	170	5.75	7	19.5

25	130	5.38	5	18.5
30	125	5.4	6.5	19.5
20	125	5.08	4.5	29.5
30	125	5	7.5	22.5
30	103	5.12	5.5	22
25	120	5.63	6	17.5
15	170	5.38	6.5	18.5
35	155	5.5	5.5	28.5
35	123	6	4	19.5
35	145	5.19	6.5	17.5
30	125		6.5	15.5
35	150	5.9	5	17
25	175	5.38	7	20
25	130	5.63	5	18.5
30	145	5.48	6.5	20.5
35	145	6.08	5.5	19.5
22	132	5.08	7	24
40	225	5.06	7	23
35	150	5.38	7	25
30	100	6	5.5	
35	145	6.18	6.5	16.5
30	170	5.28	7	18
25	144	6.02	5.5	20
40	170	6	5.5	20.5
20	137	5.9	5.5	19
40	190	5.8	6.5	13.5
40	170	5.5	7	13.5
40	160	6	6.5	20
35	185	6	6.5	20.5
25	150	5.52	6.5	18.5
30	125	5.28	5	18.5
40	135	6.18	7	19.5
35	160	5.88	6	20.5
25	125	5.38	6	19.5
35	160	4.82	6.5	28.5
30	160	5.48	6.5	18.5
30	100	5.43	5.5	18.5
20	118	5.19	6.5	18
25	130	5.8	5.5	18
40	170	5.85	5.5	20.5
30	180	5.3	6.5	20
30	160	5.38	6.5	18.5
25	116	5.17	6.5	18
20	140	5.83	4.5	23
31	120		5.1	18
23	120		6.28	15
30	120	6.9	5.84	10
32	145	5.9	6.19	
32	110	6.98	5.33	
30	130	7.07		15
32	105	6.97	5.75	
25	125	5.67	5	19
28	130	5.47	5.3	27

38	140	5.17	5.7	29
34	130	5.49	4.7	22.5
35	160	5.48	6.7	23
30	175	5.43	7.3	24.5
44	225	5.49	6.1	30
45	190	5.21	7.6	29
25	150	6	5	23
34	130	6.74	5.2	19
25	160	5.09	5	20
28	160	5.14	5.25	23
28	180	5.37	5.9	23
35	158	5.25	5	27.5
30	130	6.53	4.7	30
20	149	5.78	3.1	
30	155	5.8	3.2	28
35	160	5.82	6	29
30	150	6.2	6	25
17	140	6.4	4.5	22
30	165	5.14	5.2	25
25	135	6.07	5.6	21
28	150	5.6	5.9	24
30	135	5.63	6.7	25
30	150	4.94	4	23.5
30	160	5.03	5.5	30
30	195	5.3	7	25
33	118	8.5	4.93	
19	122	6.57	4.5	
34	130	7.2	4.5	
35	190	5.53	6.4	27
20	150	5.22	5.75	21
20	110		3.6	20
25	130		4.4	20
25	120	5.34	4.45	22
20	110	6.54	4	
20	110	5.83	5.5	
30	140	6.38	5	
40	170	5.31	5.7	24.5
40	160	5.82	5.85	23.5
30	140	5.56	5.8	
25	150	5.47	4.9	
25	130	6.67	4.73	22
30	180	5.41	6.8	22.5
30	120	5.45	5.15	
20	140	5.41	5.1	
40	160	4.66	5.8	
30	140	5.95	5.2	
35	160	5.58	5.1	
35	120	6.2	5.3	
40	160	5.53	6.2	
20	110	7.42	5.1	
30	160	5.47	4.6	
30	160	5.3	6	22
20	120	7.81	5.6	15

30	140		5.4	16
25	160	6.45	5.5	18
38	220	5.58	6.2	22
40	180	5.77	5.5	22
35	150	6.91	5.2	23
37	165	5.54	5.7	23.5
20	130	6.51	5	
25	160	6.1	5.6	
30	145	5.4	5.5	
20	140	6.41	5	
36	160	6.21	5.5	
33	152	5.82	4.5	21
16	130	8.7	6	
24	130	6.5	4.5	15
23	120	6.2	5	20
27	120	6.38	6.6	22
37	150	6.6	7.7	
38	171	6.24	7.4	22
36	140	6.3	6.6	22.5
34	180	6.15	7.1	22
37	160	6.29	5.7	22
33	180	6.02	6.3	21
20	110	6.6	4.9	
25	155	5.6	6	
20	160	6.38	6	
30	140	6.17	6.1	
30	150	5.6	6	
30	110	7.67	5	
20	110	4.5	5.5	
15	110	7	5.2	
30	130	4.4	5.6	
18	120	4.5	5.5	
25	120		4.1	22
28	130	6.3	5.4	18
28	160	6.5	4.2	20
20	150	5.12	5.05	18.5
30	120	5.34	4.2	
20	130	5.71	4.95	
20	115	5.86	5.5	
30	145	5.42	5.18	
23	125	6.4	5.3	18
24	130	6.8	5.6	20.5
15	135	6.3	5.2	16
32	140	6.71	5.8	19
30	145	5.93	5.4	
30	145	6.15	5	
38	175	5.29	6.2	
20	125	5.47	5.3	9
28	130	5.75	5	
17	120	6.6	4	
32	170	5.2	5.8	
31	110	6.2	4.5	
39	150	5.84	4.75	

36	150	6.34	5.8	19
32	140	5.37	6	
30	145	6.46	4.8	16
18	120	6.91	5.2	
30	150	5.94	6	15
37	120	6.04	7	18
41	150	4.59	7	23
35	160	5.38	6.2	
	170	5.05	6.8	23
45	150	5.69	7.4	22
30	160	6.5	6	21
20	160	5.98	6.3	24
29	160	6.73	6.81	18
30	145	6.41	5.3	
30	150	6.15	6.6	19
37	133	6.13	6.6	15
26	110	7.07	6.75	
34	148	6.35	7	
38	164	5.51	6.2	21
35	120	5.71	5	21.5
20	150	5.71	6.1	22
40	170	5.64	6	21
38	145	6.05	6.1	12
25	158	6	3.15	
43	168	6.09	7.17	19
30	160			
20	110		5.2	
25	110			21
29	120		4.5	
20	110	7.06		
25	140		5.5	
32	110	5.72	6.4	26
30	120	5.82	5.1	28.5
30	180	5.78	6.7	26.5
25	140	6.36	5	21
24	130	6.62	6.9	
28	150	6.19	5.4	24
25	110		4.2	23
25	130	6.26	6	23
15	122	6.28	5.2	29
38	194	6.09	7	25
30	140	7.71	5.2	26
25	140	6.8	5.5	26
25	170	5.88	6.5	26.5
30	130	6.38	5.4	27
40	155	6.11	5.4	21
30	165	5.29	6.5	19.5
	140	5.35	5	19
20	110	5.79	4.35	21.25
20	110	5.67	5	
29	140	6	5.25	17
25	135	7.5	4.5	17
35	135	5.84	5.9	19.5

20	120	6.74	5.2	16.5
25	150	5.25	6.2	17.5
22	140	5	5.5	19
24	110	6.94	5.4	16.5
25	130	6	5.6	19
29	125	6.34	5.3	18.5
25	125	8	5.25	18.5
29	150	5.78	7.2	19.5
26	140	6.74	6	19
35	190	6.25	5.3	18
29	150	6.5	7.1	18.5
26	140	6.32	5.5	18.75
20	115	6.37	4.9	14.5
24	160	6.44	5	18.5
20	145	5.84	6.55	22
20	120	6.79	5.7	14
29	140	5.75	5	21
24	145	6.66	4.9	19
20	130	6.13	5.2	20
48	180	5.34	8.7	23
30	170	5.85	6.6	18.5
24	155	5.75	5.8	20.5
25	130	6.35	4.7	20.5
30	160	5.22	5.7	19.5
15	120	7.03	4.8	15
35	140	4.51	5.3	18
37	160	5.35	6.2	22
32	160	6.51	5.9	21
45	190	6.3	6.7	21
30	140	7.52	4.7	19.5
27	140	5.66	5.9	18
28	140	7.03	5.9	18
30	150	6.57	6.4	18
30	150	5.96	5.7	16
33	150		6.1	19
30	150	7.38	4.7	18
20	125	6.81	4.45	18.5
30	155	5.66	5.6	20
23	135	7	6	17.5
37	155	5.6	6.2	17.5
23	130	6	5.4	18.5
24	122	6.78	5.9	16
35	151	5.54	5.2	21.75
27	130	6.24	6	19.5
25	125	7.82	6.2	17.5
27	144	6.41	6.4	20.5
18	128	7.37	5.4	16.5
31	173	6.12	6.5	21.5
28	145	6.84	6.5	18.25
30	135	5.77	5.5	18.5
35	150	6.03	5.9	20.25
30	140	6.6	5.75	18
25	150	6.07	5.5	18

25	130	7.04	5.65	17.75
30	160	5.02	6.1	16.25
25	120	7.34	6.1	18.5
25	120	7.15	5.7	20
25	140	7.09	5.25	15.5
35	160	6.04	4.6	19.5
30	140	6.02	6.25	22
25	130	7.27	5.75	16.25
25	120	6.06	5.2	17.5
30	140	5.02	6	16.5
25	120	6.6	4.75	17.5
30	120	6.06	5.5	15.5
30	130	5.97	4.9	20.5
30	140	7.09	5.6	18
30	130	6.06	5.9	19.5
30	120	5	6	19
30	135	6.89	6.1	16.5
30	120	6.99	3.6	19.25
25	153	6.34	6.6	19.25
35	131	6.03	4.1	23
22	138	6.54	3.6	21
26	168	5.56	5.3	28
24	163	5.85	4.2	27.5
25	179	5.85	3.64	24.5
28	155	5.65	4.9	28.5
32	165	6.95	4.4	27.5
21	156	6	4.5	25
28	144	6.93	4.9	24
20	128	6.11	3.75	21
23	145	5.68	4.4	24
25	150	6.38	4.5	25
31	166	6.06	5.2	24.5
29	105	6.34	4.6	18
30	176	6.47	4.6	18
25	153	5.88	4.3	21
26	165	5.43	5.48	22
31	168	5.66	6	18.5
24	162	6.05	5.9	20
25	138	6.5	3.9	20
23	142	6.89	6.2	16.5
23	134	6.5	5.18	21
26	129	6.94	5	17.5
32	169	6.44	5.8	24.5
28	130	5.79	5.9	26.5
26	135	6.23	6.1	24
25	125	5.81	4.3	21
19	120	7	4	21.5
30	156	5.85	4.3	19
26	122	7.22	5.2	22.5
18	130	7	4.85	21.5
29	155	5.41	5.3	24.5
26	136	5.75	4.3	24.5
25	149	5.59	4.3	21

27	148	6.22	5.3	20.5
32	190	5.7	6	20
35	159	5.75	5.8	19
35	199	5.21	5.9	18
35	180	5.93	4.9	21.5
30	142	6.72	4.6	15.5
35	145	6.06	5.9	16
30	151	6.43	5.4	13
25	155	6.51	4.1	20
35	178	6.14	4.5	25
28	158	6.1	5.6	19
30	133	6.93	4.1	15
15	144	5.84	5	18.5
30	157	5.92	3.7	18
35	168	5.9	4.25	25
25	130	6.81	3.55	19
25	119	6.97	3.6	20
21	115	6.96	3.8	18
26	161	5.79	4.9	16.5
30	163	5.81	5.4	21.5
25	121	6.82	4.2	13.5
32	174	5.36	5.9	27.5
27	151	6.56	5.1	18
30	172	6.31	4.3	17
35	175	6.65	5.2	27
40	177	5.35	6.9	30
25	142	5.71	4.9	29.5
27	151	5.67	5.6	17
23	122	6.16	6.2	17
22	110	6.88	6.3	
27	143	6.28	4.6	
20	122	7.25	4.3	20
21	119	6.49	5.3	25
20	143	5.86	4.7	21
26	182	5.6	6.1	23.5
27	177	5.89	5.7	23
23	150	5.91	5.4	19.5
28	152	5.97	5.6	19.5
26	151	5.67	6	21
19	147	5.51	6	20
23	158	5.19	5.3	21
28	134	6.31	4.6	19
19	122	6.79	5.4	22
28	143	6.69	5.6	24
29	141	5.59	6.1	24
25	166	5	6.5	25
19	128	5.93	6.2	23.5
23	119	6.31	2.9	22
23	153	5.88	2.8	19
25	140	6.3	6.1	22
25	152	6.19	5.2	21.5
32	157	5.41	4.9	26
28	168	6.21	4.7	19

25	145	5.84	5.2	20
29	144	5.91	6.3	12
18	123	6.01	4.8	21.5
23	127	5.73	4.8	24
23	156	5.99	6.2	22.5
19	124	6.86	4.8	22
18	125	6.91	6.2	8
27	129	6.28	6.4	21
25	164	6.31	4.8	21
27	159	6.19	6.3	24.5
30	136	5.77		24
30	178	5.94	5.4	19
19	126	6.19	5.5	24
20	138	6.5	5	10
30	168	5.66	5.2	25
21	141	6.55	5.6	23
20	130	6.57	4.8	28
31	169	5.81	5.4	24
30	171	5.59	7	27
21	127	6.27	4.9	24
24	169	6.02	5.4	22.5
22	143	6.1	5.4	24.5
38	172	5.71	6	28.5
16	141	6.1	4.9	24
25	171	5.99	5.9	23.5
13	163	5.78	4.9	24
28	164	5.85	5.7	19.5
19	156	5.71	5.6	24.5
26	148	6.79	6.1	21.5
16	141	6.53	5.2	19
29	160	5.6	4.9	25
28	172	5.78	5.3	22
32	159	6.02	5.12	26.5
28	168	5.5	6	25.5
29	139	6.56	4.39	23
26	158	6.06	5.6	26.5
23	150	5.9	4.65	25
22	160	6	5.2	22
23	145	6.82	4.5	18.5
24	151	6.41	5.55	24
24	168	5.63	7.2	19
27	140	6.16	5.05	25
24	110	7.14	2.8	21
23	164	6.39	5.25	18
21	164	5.81	5.25	23.5
35	110	6.07	5.55	21
27	120	6.07	6.55	21
26	100	6.89	5	18
35	135	5.81	5.1	19
31	135	5.34	5.55	19
27	105	6.04	4.5	
24	115	6.74	5.5	18
24	125	6.41	5.1	18

31	130	5.3	6	21
30	140	5.71	5.5	22
28	130	6	5.5	19
26	125	6.75	5.55	22
25	110	6	4.55	21
39	125	5	6	21
19	100	6.78	4.5	16
24	110	6.04	4.5	21
23	130	6.9	6	21
47	155	5.05	6.55	27
26	125	5.78	6.5	17
21	110	6.03	4.5	22
23	140	6.05	5.32	20
26	140	6.93	4.55	21
28	130	5.9	5.45	
30	130	6.05	5.5	19
31	160	6.09	5	19
37	170	5.17	5.5	
24	150	6.21		
40	165	5.07		
30	130	6		
37	170	5.17	5.5	
28	150	6.03	5.5	23
24	110	6.93	3.5	17
36	100	6	5	20
25	120	6.77	5.28	
30	130	6.13		
18	125	6.82	5.74	19
28	147	6.54	2.8	18
18	115	6.83		
33	130	5	4.7	26
30	120	5.91	4.7	22
26	145	5.9		
20	130	7.94	5	
20	120	6.35	4.93	19
33	150	5.57	6	19
32	125	5.66	4.5	22
27	100	7		
20	120	7.06		
33	130	5.93	6.5	19
30	130	5.22	7	23
34	150	6.82		
31	130	5.9	6	18
25	130	5.86	6.1	22
31	120	6	6.5	19
27	160	6.03	5.5	19
28	160	5.71	6.15	22
42	140	5.95	6.55	22
31	140	5.71	7.1	22
30	140	6	5.25	
25	115	5.6	5.55	22
26	130	6	6.1	21
27	135	5.94	6.1	22

28	150	6.01	6.5	22
27	140	6.01	5.5	29
20	100	6.57	4.55	15
34	140	6.52	7.1	20
22	110	6.5	5.5	19
38	150	5	5.5	22
22	120	5.84	5.5	20
19	120	6	5	20
29	130	5.56		
24	130	5.46	5	
27	125	5.86	5.1	21
28	115	7	6.45	24
26	130	6	4.5	19
18	120	6.17	4.25	19
22	150	5.03	6	20
15	100	7.2	4.55	19
27	150	6	6.25	21
23	120	6.06	5.1	24
15	90	7	4.5	17.5
27	170	5.21	7.35	23
28	110	7	4.5	22
26	125	6.07	5.5	22
30	125	7.01	6	18
27	140	6.04	5	19
32	150	6.23	5.55	22
30	125	6.1	6	13
17	100	8.32	4.55	19
28	110	6		
23	80	6.12	4	19
30	130	7	4.44	19
23	90	6.01	6.37	22
25	100	6.02	5.65	22
25	110	7.15	5.8	24
26	130	6.17	5.85	
30	140	6.19	6	20
33	130	5	6.34	22
25	130	6.04	4.87	21
23	120	6.07	5.5	18
23	100		5.5	17
26	120	6		
28	130	6		
26	135	5.7		
28	130	6		
28	145	7.01		
16	100	6.07		
30	125	9.71	4.97	17
18	154	7.71	5.28	20.5
21	118	9.39	4.25	
35	168	7	7	22
26	157	9.07	5.48	21.5
21	140	8.21	4.7	21
14	145	8.45	4.85	21
22	180	7.9	6.36	

24	174	7.95	6.33	
19	160	7.63	4.93	21
17	160	7.9	4.3	21.25
23	129	8.99	4.77	
31	200	7.47	7.4	21.5
15	145	8.51	5.16	
21	180	8.12	6.35	
13	140		4.8	
22	155	8.63	5.3	21.5
15	121	9.05	3.9	20
22	145	8.43	5.45	21
19	130	9.47	5.3	
13	126	9.88	4.75	
29	192	7.94	6.8	22
16	145	8.56	5.1	20
29	144	8.67	6.9	20.5
27	128	5.78	5.7	20.25
27	133	6.99	5.52	19.5
33	145		5.4	19.75
28	153	5.58	8.26	18
34	156		6.3	22
43	180	5.16	7	24
33	132		6	22.5
35	141	5.96	7.06	21
37	183		6.2	23.25
42	185	5.59	8.9	20.5
37	147	5.9	6.45	22
32	155	6.38	6.75	16.5
39	183		7.25	19.5
34	142	6.4	5.8	8
27	131	6.5	6.23	20
39	129	6.56	5.3	18.75
43	124	5.94	7.1	18
29	134	7.22	5.3	20.5
33	166	5.5	5.95	
35	144		5.3	8
40	147	6.25	6.72	19
40	147	5.82	7.1	
30	158		6	18.5
32	158		7.3	17
35	141	5.91	5.4	19.5
30	126	6.18	6.46	19.75
37	151		6.4	19
33	152	6.53	6.26	23
35	168	6.03	6.6	
38	196	5.87	6.25	19.5
36	119	6.8	5.55	20.25
30	156	6.08	5.75	16
36	124	6.59	6.6	11
44	170	6.4	7.12	24.75
32	137	6.03	7.2	19
28	109	7.35	5.82	23.5
41	155	5.07	7.44	20

29	145	5.56	5.93	19
22	143	6.44	5.27	19
48	136	6.12	7.5	21.5
43	145	5.93	6.45	19.25
30	127	6.37	5.32	20
27	111	7.44	6	17.5
	135		5.9	
	158	6.03	6.95	21.25
	110	6.99	4.9	20
	138	6.6	6.3	20
	147	6.08	7.7	21.5
	125	6.77	4.6	19
	113		5.4	
	139	6.9	5	19.75
	140	6.2	5.5	13
	136	6	6.25	22.75
	120	6.81	6.45	
	137	6.61	5.55	20
	128	6.41	6	9
	147	5.87	5.9	10
	135	6.02	5.65	22
	131	7.31	6.37	
		7.7	5.2	
	125	6.84	5.7	17.5
40	140	5.2	5.89	18
33	139	5.48	6.3	7
36	125	5.43	6	7
30	123	5.6	5.6	
31	114	6.4	4.88	17.25
36	119		5.59	10
35	150	7.69	5.89	11
33	131	6.7	5.78	19.5
33	140	5.22	4.7	22
22	133	6.66	4.53	24
37	175	5.47	7.48	20
45	158	5.95	9.02	20
29	115	7.74	5.88	19
	141	5.15	5.5	21.75
	132	7.03	6.2	15
32	123	6.79	6	18.25
27	128	7.2	6.45	20
43	202	5.28	7.94	22.5
33	132	6.19	5.08	21
34	158	5.9	6.76	23.25
25	135	6.51	5.5	21
25	130	7.01	6.34	18
37	139	6.02	6.99	26
27	132	7.1	6.57	18.5
25	153	5.71	6.34	17
38	150	6.03	5.09	21
17	127	7.01	5.44	20
30	145	7.23	5.68	18.25
23	116	7	5.06	19

24	109	7.22	5.46	16
33	154	5.27	6.16	21.25
35	168	6.18	6.9	21
25	107	6.51	5.4	18.5
27	142		6.2	16.5
27	148		5.85	18
29	130	6.27	6	21
38	139	6.07	6.1	20
40	140	6.11	7	23
30	126	6.19	6.2	17.5
33	125	7.01	4.9	18
25	158	5.14	5.7	21.5
32	152		7	21.5
37	160	5.29	6.95	22
25	119	6.05	7	20
30	125	7.2	5.62	15.25
28	114	5.9	5.2	20
42	141	5.79	5.8	
52	124	6.45	6.2	19.25
20	123	7.5	4.34	19.25
44	153	6.61	6.1	20.5
29	137	5.59	6.16	21
39	142	6.51	6.46	20
42	147	6.35	6.44	20.75
42	149	5.67	5.43	19
20	129	5.7	5.94	18
30	152	5.55	5.59	24
31	122	5.22	6.13	19.5
24	129	6.79	5.43	20
36	162	6.47	6.35	19
27	134	6.41	5.27	23.5
22	142	7	6.1	17.5
31	148	6.51	5.83	20
46	140	6.25	5.12	20
26	130	6.77	6	20.5
34	144	6.11	6.95	19.5
43	183	5.25	8.21	22.25
34	159	5.65	7.15	19.75
28	148	6.02	7.23	18
33	157	5.88	5.7	20
38	167	6.01	7.2	20.75
24	119	6.5	6.35	19.5
36	152	5.93	6.71	19.5
35	156	5.8	7.77	19.25
26	123	6.53	5.83	
31	142	6.27	6.6	
42	186	4.4	7.5	21
36	147	5.74	6.3	16
38	161	5.57	7.22	
32	123	6.27	5.6	20
31	141	5.72	5.73	19.75
30	102	7	5.8	9
31	178	5.93	6.35	18

29	142	5.96	7.15	
28	180	5.58	6.14	22
30	146	6.01	6.55	19.5
39	145	5.84	5.5	18.5
30	112	7.53	4.9	19.75
30	156	5.13	6.55	18.25
37	159		6.17	
	138	5.65	5.26	20.75
33	120	6.09	5.5	23
22	110	7.75	5.6	18.5
29	160	6.12	6	17.25
24	110	6.78	5.6	19
30	120	6.34	5.6	18
22	110	6.87	5.9	18
31	160	6.56	6.5	21.5
24	130	6.4	6.5	20
36	110	5.96	6.1	17
36	130	5.5	5.5	16
37	110	6.62	6	18
24	160	5	7	14
40	160	5.81	7.5	12
27	150	6.2	5.5	12.5
23	90	7.21	5.5	19
	170	4.9	7	24
34	150	5.45	6	23.5
26	100	5.89	5.5	13
42	140	5.68	6.2	14
28	140	6.28	6	18.5
22	130	6.07	5.6	16.75
17	140	6.9	5.5	19.5
31	140	6.37	6.5	14.5
23	100	6.71	5	18.75
33	110	7.56	6	19
27	140	6.12	6.5	19.5
24	115	6.9	5	14.5
31	100	5.96	5.5	19.5
26	125	7.03	7	15
46	160	6.3	6.5	20
19	80	7.2	5	10
	145	5.26	6.5	23
37	115	6.1	5.75	20
37	140	5.68	5.85	19
23	140	7.5	5	10.5
40	115	5.4	5.5	18
37	130	6	6	25
25	100	8.12	4.5	16
23	130	6.65	5.5	14
33	120	6.09	5.5	23
25	120	6.5	6	22
37	160	5.83	6	24
32	130	5.45	6.2	24
23	130	6.37	6.5	17
22	130	6.26	5.5	19

24	160	5	7	14
40	160	5.82	7	12
26	150	6.2	5.5	12.5
25	100	5.89	5.5	13
42	140	5.68	6	14
	170	4.9	7	24
27	120	6	5.5	19
30	140	5.88	5.5	24
35	130	6.15	5.2	13
33	120	6.2`	5.7	22
43	130	5.84	5.5	18.5
33	130	6.5	5.5	18
30	130	6.71	6	14.5
24	150	6.7	5.5	18
39	172	5.3	7.5	22.5
29	130	5.8	5.5	17.5
30	135	6.76	7	18
25	130	7.62	5.6	17.5
20	150	6.21	5.6	18
27	120	6.78	6.2	17.75
28	120	6.78	7	19.5
31	150	5.75	7	24.5
24	150	6	6	28
27	120	7.25	4.5	20
34	150	5.4	6.5	26
37	130	5.21	6.55	19.5
24	120	6.71	4.5	15
22	100	6.68	4.5	16.5
21	120	7.35	5	
21	150	5.5	6.2	21
32	140	6.81	5	15.25
35	130	6.21	5.5	21.5
33	170	5.9	6.2	18.5
27	150	6.25	5.7	16
32	170	6.53	5.7	20
33	190	6.09	6.7	20
26	130	7.09	6	19.5
30	160	5.59	6.8	19.5
25	130	6.49	6.8	15.5
28	160	6.22	6.5	19
30	170	6.01	6.8	17.5
20	150	6.4	5.8	18
30	180	5.28	7.5	23.5
30	120	6.73	5.8	15
40	160	6.22	6.8	17.5
20	100	8.03	5.8	16.5
25	180	6.15	6.5	20.5
35	160	6.36	6.2	22
20	120	7.03	5.2	18.25
30	170	6.3	7	20.5
30	170	6.01	5.8	20
16	120	6.09	4.8	19.5
20	180	5.98	6.8	20

40	150	5.55	6.5	18.5
30	162	6.21	6.5	21
40	200	5.83	6.5	21
30	130	7.19	5.8	16.5
20	140	6.74	5.5	15
30	180	5.72	6.8	19.5
30	170	5.41	7.4	20
29	150	6.25	6.2	17
28	185	5.54	7.2	19.5
35	190	5.25	6.7	22
26	150	5.87	6.9	20
30	150	5.93	6.5	20.25
36	170	5.34	6.9	22
25	140	5.78	5.9	19
30	140	6.16	6.7	19.5
30	160	6.75	6.9	18
22	160	6.78	7	19.25
36	160	5.53	6	20
24	150	6	6.3	19.5
35	160	6.1	7.5	18
36	140	6.24	5.9	19.5
29	180	5.91	7.5	21
26	160	6.19	7.9	16.5
29	160	5.82	5.5	20
26	160	6.19	8	19
20	140	6.22	6	20
25	150	6.03	6.5	18
22	150	5.75	6.4	17.5
20	120	7.85	6	16
32	140	6.31	7.6	19.5
30	150	6.21	6	21.5
		7.5	5.6	
18	136	7	5.4	18.5
18	121	7.14	4.65	18.5
24	135	6.78	5.35	22
20	118	7.71	5	16.5
24	111	8.1	4.9	17
24	122	7.59	4.4	18.75
	168	6.92	5.1	19.5
25	150	6.63	5.1	18.5
29	160	5.53	5.15	18.5
	119	6.44	4.4	19
30	150	6.69	5.1	21
20	122	7.81	5.55	17.5
24	135	6.82	4.45	21.5
35	145	7.31	4.8	19
25	150	6.34	5.5	20
20	138	6	5.6	20
21	134	7.35	4.7	20.5
22	129	5.82	4.5	21.5
25	156	6.32	5.1	21.75
12	125	7.59	4.5	19
28	175	6.15	5.2	20

18	110	7.48	4.7	
47	137	6.51	5.3	21
34	160	6.29	5.2	20
27	151	6.47	6.65	19.5
25	122	6.98	6.25	18
25	111	7.4	5.9	16
25	118	7.21	6.3	15
20	135	7.23	4.8	17
22	133	6.51	6.1	19
24	141	6.98	5.45	21
31	162	5.86	6.5	20
30	155	5.9	6.7	19.25
31	131	6.73	6.4	20
34	175	5.84	6.3	24
28	160	6.65	5.4	20.5
17	117	7.77	6.2	17.5
26	127	6.55	5.5	16.5
20	109	7.68	5.4	17.5
30	180	5.86	7.5	20
16	127	7.66	4.85	16
28	134	5.6		21.5
25	140	6.51	5.45	
29	163	6.66	5.7	21
25	140	5.98	6.2	20
25	120	6.79	4.2	19
20	131	6.91	5.3	16.25
31	160	6.01	6.35	20
21	118	7.4	5.4	20
27	132	7.21	5.7	18.75
12	95	9.79	5.4	
19	125	6.78	6.5	18.5
26	154	5.91	5.85	22.5
28	140	7.32	5.85	22.5
34	155	6.42	7.05	21
24	125	6.98	6.2	17
37	180	5.63	7.9	25
24	135	6.59	6.1	20
33	164	6.33	5.9	20
27	124	6.19	5.45	15.5
29	165	6.31	5.9	20
26	135	7.35	5.45	
30	148	6.21	5.85	
12	101	9.93	4.7	16.5
10	120		4.8	18.75
15	150	9.15	4.3	16
37	136	8.59	5.25	17.75
24	154	7.38	5.38	19.5
23	164	8.57	4.62	20
40	180	7.78	6.16	20.5
24	171	7.3	6.09	24
24	154	7.84	5.28	23
19	123	8.03	5.14	
17	134	8.38	4.25	20.5

19	104	8.21	4.28	16.75
35	167	5.15	5.26	20
36	166	5.55	5.95	22
34	150	6	5.7	16.5
25	125	6.3	5.95	18
27	123	8.06	5.6	9.5
35	144	6.23	4.6	12.75
20	117	7.3	5.2	13.5
21	146	7.37	3.9	13
27	159	5.8	5.2	11
26	108	6.05	4.75	16.25
24	146	5.96	5.15	12
18	125	7.5	4.76	16.5
33	156	5.36	5.75	16.5
27	120	7.2	5.95	11.5
32	157	5.46	5.26	18.75
33	116	6.8	4.88	12.5
35	135	7.06	5.86	18
41	167	5.5	5.9	18
15	150	8	4.92	12
28	125	6.3	6.38	15.5
40	170	5.31	6.57	23.5
36	160	5.9	6.52	19
27	145	5.31	6.95	17
25	122	6.29	6.6	16
34	135	5.5	5.65	21.5
24	125	6.2	5.8	19
32	140	5.5	6.65	19.5
25	152	6	5.8	16
27	110	6.06	6	16.5
22	130	6.25	6.67	16
44	165	5.59	5.83	23.5
24		6.75	4.82	19
35	155	5.28	8	17.75
42	172	5.8	6	20
27	145	6	5.05	20
44	175	5.3	7.7	22
27	130	5.87	6.5	18
28	150	5.56	4.5	21
26	148	5.53	5.55	19
34	132	6.53	5.6	19
27	115	6.5	4.68	18.5
35	148	6.5	6.73	18
51	155	4.95	7.02	22.5
23	115	6.9	4.7	19
35	160	5.62	6.52	19
39	130	5.75	5.5	22
30	156	5.8	5.45	17.5
23	130	7	5.45	16
22	135	6	4.23	21.25
39	175	5.27	6.58	20.25
26	127	6.52	5.84	20
28	135	5.33	6.45	17.5

28	115	6.52	5.94	16.5
33	140	6.2	5.85	13.5
40	130	6	4.4	15
25	145	6.2	6.72	15.5
31	165	5.38	8.4	16.5
25	150	6.8	5.1	18.5
34	158	5.6	6.05	20.5
25	115	6.53	6.8	17.5
22	130	6.1	4.5	17
34	125	6	5.85	19
29	125	5.83	6	21
30	142	6.31	3.95	16.5
23	115	6.44	3.73	16.5
24	140	6.05	5.35	19
40	145	6	6.25	19
24	115	6.5	3.5	16.25
31	138	5.78	5.86	18.5
35	135	5.71	6.62	19
27	138	5.87	4.25	17
28		9.4	5.72	10
36	138	5.21	6.6	15.5
36	160	5.82	5.54	20
35	165	6	6.26	20
28	153	5.81	5.73	17
26	135	6.05	5.8	19
24	140	5.68	5.52	13
35	163	5.78	5.9	20
26	144	6.25	4.2	16
21	135	6.9	3.7	16
28	143	6.24	4.2	16.5
19	132	6.12	4.3	18
23	149	5.78	4	17.75
30	136	6.78	3.8	17
22	136	6.15	4	18
21	135	6.59	4.1	19
20	163	5.93	4.7	20
28	178	5.98	3.8	19.25
22	152	6.3	4.5	17.75
21	142	6.34	4.5	17
21	125	6.4	4.2	16.75
25	137	6.2	4.2	16.75
28	160	6.78	4.7	18
28	180	5.53	3.8	20
27	165	5.78	4.3	19
17	140	6.21	4.4	19
28	169	5.68	5.6	17.5
22	170	6.71	5.5	16
30	146	5.25	6	22
27	145	6.12	4.5	18
30	142	6.5	4.7	18.25
34	133	6.23	3.8	16
26	160	5.31	3.9	19
21	165	5.68	4.8	19

30	148	6.16	5.5	18.75
19	164	5.35	6.1	17
31	152	5.45	5.5	16.25
16	105	7.1	4.7	14.5
30	150	5.47	7	18.25
28	179	5.21	5	20.75
22	162	5.75	6.2	17.25
28	130	6.68	5.3	18.75
24	126	6.63	5	17
23	153	6.63	4.8	19
23	140	6.22	6	17.75
18	158	6.28	4.8	16.75
18	133	6.35	5.8	15
22	142	5.37	4.2	15.75
22	145	5.43	4..5	16.75
28	150	5.9	4.7	18.75
25	142	4.81	4.7	19.5
27	162	5.59	4.6	17.75
22	130	5.34	5.1	18.25
20	166	5.75	4.83	14.5
15	145	5.93	4.6	19.5
22	124	6.53	5	13.51
			5.2	18
33	177	5.34	6.2	17.75
28	153	5.9	5.2	15.75
20	131	5.78	5.5	15
36	125	6.84	4.5	15.75
16	121	6.21	4.8	15
24	139	6.43	6	16.75
29	158	6	6	15
10	109	7	4	13
18	127	6.43	5	17
26	154	6.34	5	17
22	165	4.93	5.8	16.75
28	170	4.87	5.2	20
26	172	4.53	5.9	18.75
30	130	5.18	5.3	15.75
27	145	6.9	4.35	15
23	125	5.48	5.7	15.5
21	148		4.1	
28	166		5.5	
27	160		4	18
20	138	5	4.99	20
34	172	4.65	6.32	18
25	116	5.37	6.07	16
21	141	5.18	6.36	18.75
24	171	4.81	5.71	20
27	155	5.82	6.3	17
31	163	4.81	6.1	18.75
22	173	5.31	5.15	19
23	137	5.31	6.34	21
27	174	5.31	4.86	16.25
22	167	4.87	6.39	17

33	172	4.78	6.06	19
30	144	4.9	5.38	17.25
23	171	5.46	5.72	18
23	193	4.93	6	19.75
26	134	5.5	5.41	
24	157	6	4.9	16
26	145	5.6	5.4	16
27	140	4.98	5.64	
32	150	5.78	6.11	17.25
13	100	6.6	3.38	18.75
31	180	4.65	6.2	25.5
21	137	6.61	5	19.75
20	143	5.9		
30	161	6.31		
24	135	6.56	5.85	17.25
23	162	5.93	7.87	20
27	168	6.25	6.1	19
20	128	7.2	5.33	17
28	126	7.6	5.3	18
21	155	5.68	4.39	17.25
21	159	6.34	5.17	17.5
24	159	5.62	6.74	17.5
21	120	6.68	5.2	18
21	150	5.71	3.7	17.25
23	157	6.2	5.66	17.25
25	158	6.63	4.14	22
33	180	5.56	5.95	18.5
24	134	5.82	5.45	19
31	136	6.16	5.62	16.5
34	186	5.4	5.65	20
24	191	6.62	5.65	17
29	137	5.98	3.73	17
38	160	5.53	5.47	19.5
19	136	6.21	6.55	19
25	158	5.68	6.1	12.75
25	171	6.53	4.6	14.5
17	134	7.6	5.5	
29	156	6.19	6	15.5
18	157	6.25	5.2	20
20	135	6.94	4.8	19
21	130	7.16	4.8	13.75
27	135	7	5.25	19
27	151	6.34	5	18
15	145	6.19	5	14.25
22	140	7.18	6	18.75
17	120	6.18	6	20.25
22	145	6.44	4.8	21.5
16	110	7.5	5.3	18.75
18	138	6.9	4.8	17.5
24	130	7.16	6	13.25
33	146	6.53	4.8	16
25	175	5.59	6.5	15.25
20	150	5.75	5.4	20.75

21	122	6.78	5.4	21
29	150	6	4.9	18.5
17	130	6.66	2.5	19.5
26	160	4.95	5.2	23.5
26	138	6.46	4.33	22
26	160	5.35	4.99	16.5
19	140	7.2	4.07	17.5
20		7.19		
33	110	7.47		
25	185	5.72		
21	144	6.25		
30	186	5.68	5.24	22.25
25	173	6.56	6.48	21
26	139	6.58	6.58	20.25
22	150	5.96	6.48	20.25
23	170	5.71	6	19
36	172	5.93	6.96	23.75
21	150	6.18	5	19.25
28	132	6.7	5.96	15.5
20	142	6.5	6.24	16.75
30	150	6.61	5.64	18.75
19	149	6.79	4.37	19.75
34	164	6	5.95	21
23	133	6.8	6.53	17.75
34	200	6	7.24	21.75
33	196	5.6	5.49	20.25
29	150	7.12	6.9	19.5
22	132	7.03	5.24	18.75
26	150	6.81	6.52	17.25
35	150	5.44	4.92	14.25
28	153	6.15	4.59	18.25
30	165	6.74	5.9	21.75
35	180	5.66	5.3	20
25	123	5	5.29	21.75
26	145	6.7	5.47	
25	225	6.77	5	21
26	115	6.66	6.4	19.5
30	140	6.02	6.2	18.5
25	140	6.69	5.5	22
25	105	7.17	6.3	17
25	125	5.97	4.2	24
30	135	7.6	6	19
20	130	6.9	4.8	21
20	125	6.31	5.5	21
28	120	7.92	5.55	14.5
22	132	7.14	5.9	19
43	164	5.03	6	20
37	117	6.79	5.9	19
38	145	5.46	5.1	22
25	131	6.09	5.67	18.5
25	120	7.13	5	
33	128	6.71	4.65	14
31	112	6.74	6.7	17

33	135	5.47	5.1	
31	121	6	6.5	22
26	252	6.69	6	19
21	140	6.15	7	22
34	275	6.8	4.8	20
20	160	8.24	6.5	15
30	145	6.17	5	10
20	115	6.07	5.8	23
25	229	7.49	5	19
25	120	7.05	5	18
34	140	7.49	6.5	8
25	120	6.13	6	20
25	145	6.16	6	17.5
21	122	6.75	6.2	17
21	227	7.28	6.4	17
24	244	6.81	4.9	19
31	236	5.78	5	17
20	245	6.53	5.05	19
24	145	5.6	7.35	19
30	135	5.06	5.85	20.5
35	135	5.93	5	18
33	125	6.75	5.65	16.5
30	105	6.53	5.3	20
28	125	6.05	5.35	18
25	136	5.01	5	15
25	120	6.12	5.7	16
22	130	7.19	4.95	19.5
	105	5.45	5.8	20
30	165	5.28	6.1	20
	115	6.02	3	15
15	105	6.77	5	19
37	250	5	4.9	26.5
30	210	7.22	4.4	18
25	225	5.78	5.2	21
35	220	5.69	4.8	22
15	215	6.72	4.8	20
20		7.89	5.1	12
27	135	7.57	6.5	20
	135	6.75	6	16
20	140	6.77	5.8	22
20	125	6.56	5.2	20
25	130	7.22	5.5	24
24	175	5.77	6.2	20
25	210	5.38	3.5	22.5
20	180	6.93	5	19
24		6.27	5.4	22
20	195	6.93	5	19
30		8.14	5.6	23
25	125	6.74	5.1	18
25	110	6	5.45	18
31	125	6.2	5.75	19
30	120	6.29	7	22
30	120	5.03	4.1	

25	165	5.02	6	24.5
30	158	5	6.3	25
22	162	5.71	5.7	
30	110	7.53	5.55	18
25	130	5.04	5	12
20	120	6.06	4	
20	142	5.6	5.6	16
25	125	5.01	4.1	17
25	125	6.4	4	18
27	129	6.78	4.1	17
25	117	6.02	5	19
35	140	5	7	19
30	140	6.01	5.4	19
30	143	6.33	6	18.5
25		6.64	5.1	19
	140	6.34	5.15	19
	105	6.89	5	18
	110	6.83	6	18
	124	6.48	5.15	18
35	135	5.72	6	23.5
	115	9.1	5.2	13
	130	6.2	5.8	17
	145	6.94	5.3	18
	185	6.1	6.2	17
26	98	6.02	5.5	19
33		6.8	4.5	20
24		6.7	6	20
32		6.37	5.9	16
30		5.82	4	21
30		6.58	5.8	19
28		6.58	5.5	20
20		6.19	5	19
30		6.3	5.6	16
23		6.31	4.9	21
24	160	7	3.9	18
25	250	5.41	5.6	22
35	110	6	5.1	21
25	140	6.21	4.6	18.5
27	150	6.31	5.3	22
27	150	5.84	6	21.5
23	160	6.06	6	20.25
25	140	5.94	4.9	19
24	120	6.13	4.8	21
31	170	5.21	5.6	23
30	145	7.35	4.5	21
22	90	7.21	5.6	20
32	130	6.51	5.5	20
35	140	6.91	4.3	21
22	123	5.56	4.3	20
28	155	5.75	5.1	20
24	135	5.65	5.3	23
21	115	6.4	5.3	20
29	170	5	5.6	22

26	120	7.12	5.5	23
33	160	5.3	5.8	22.5
25	130	6.53	5.6	22
23	102	5.9	4.6	21
20	133	6.91	4.7	21
26	130	6.19	4.7	22
24	140	5.91	5.1	21
36	175	5.49	5.2	22
26	162	5.77	4.8	24
33	130	5.22	5.2	19
27	125	6.2	4.7	18.5
27	170	5.18	5.9	22.5
22	110	6.35	4.45	20
28	125	5.85	5	21.5
33	165	5.25	4.4	21
43	160	5.21	8.3	22.5
24	140	5.38	5.3	21
30	160	5.21	8.3	22.5
20	130	7.21	3.6	17
22	130	7.21	5.4	17
22	140	6.93	4.55	18.5
29	170	7.21	4.8	18
28	155	5.5	5.6	23
42	161	4.94	6.1	24
23	130	5.99	4.7	20
27	111	6.5	4.7	16.5
20	140	5.81	5.8	19
35	170	5.25	5.6	21
27	160	5.95	5.5	21.5
32	150	5.68	5.1	21
39	160	5	8.3	22
25	140	5.99	5.8	21
27	170	6.02	5.6	19.5
28	160	5.19	7.3	21
35	160	5.45	5.9	19
23	130	7.21	5.5	18.5
25	140	5.49	5.8	19.5
25	150	5.91	5.1	20.5
27	120	7.35	4.2	17.5
27	140	7.31	4.3	18
30	170	7.01	5.1	19
43	160	5.12	5.8	23
	90	7.41	4.6	17
	90	7.36	4.3	18
	132	6.23	4.7	18.5
	140	6.13	5.7	18.5
	130	5.87	4.2	23
	145	6.9	6.1	20
	120	6.36	4.7	20
	130	6.1	4.5	21.5
	132	5.5	5.05	21
	160	5.45	6.2	23
	133	5.5	4.9	23

	125	5.57	6	23
	120	7.06	4.9	17
	190	5.41	5.5	21
	220	5.12	5	22
	200	5.18	6.5	23
	140	5.41	4.7	23.5
	110	5.82	4.8	24.5
	128	6	5.1	21
	140	5.3	5	21
	130	6.81	5.6	21
	160	6.31	4.9	18.5
	130	6.03	4.7	19
	80	6.63	4.7	13
	160	6.41	4.8	19.5
23	190	5.9	5.7	19
30	165	6.4	5	18
19	165	6.8	4	19
30	160	6.1	6.7	19
20	130	5.9	4.7	18
30	140	5.72	6.9	20
20	115	6.28	4	18.5
44	120	5.9	5.1	18
27	145	5.21	6.9	
23	165	5.35	5.3	20.5
27	125	6.62	4.5	20
24	125	5.82	5.2	20.5
31	140	6.4	5.5	20
25	150	5.9	5.5	21
25	120	7.1	4	17
25	120	6.4	5	18
17	125	6.5	4.1	18
27	105	7.5	5.8	20
25	135	6.19	5.7	22.5
22	130	6.19	4.7	21
26	135	5.81	5.65	21
36	175	5.36	6	29.5
27	150	5.9	5.8	19.5
28	170	6.51	6.6	23
30	190	6.3	6.2	19
25	130	7		17
22	120	6.8	4.7	
35	150	6.4	5.8	17
32	165	6.2	5.3	20
28	145	6.15	5.1	19.5
27	140	6.03	5.8	22.5
22	130	5.84	5.2	22
25	170	6.06	6.4	21.5
35	150	6.53	6.6	25
28	185	5.9	4.6	20
25	160	5.91	5.5	22
30	160	5.8	4.3	21
30	160	5.82	4	25
15	110	6.15	5.2	21.5

29	175	6.1	5.7	18
24	170	6.32	6.2	22
29	155	6.46	5.75	17
25	160	5.56	5.4	21.5
33	160	6	4.3	24
25	125	5.96	5.3	22
29	170	5.4	5.8	21
40	160	6	6.5	20
20	150	5.4	7.3	21
30	160	6.08	3.7	20
35	170	5.9	5.8	17
22	135	6.66	5.3	22.5
19	120	7.05	5.75	17.5
27	140	6.07	5.7	20
25	140	5.8	5	20
24	160	6.16	5.4	23
24	130	6.16	6.5	21.5
27	175	5.65	5.3	22
27	152	6.2	7	18
23	160	5.9	4.8	17
20	110	7	5.3	17
34	165	6.48	5.3	19
32	150	6.74	5.3	18
30	160	6.37	4.6	18
28	130	6.4	5.3	18
27	160	5.38	5.3	18
22	160	6.4	3.9	18
35	190	6.1	5.2	18
25	145	6.74	5.6	18
33	150	5.68	4.2	19
25	115	5.85	5.3	18
20	150	5.45	4.9	19
25	150	5.48	5.1	18
30	150	5.88	5.4	21
26	140	5.75	5.8	19
20	115	7.2	5.2	
25	180	5.8	5.9	18
25	155	5.65	5	19
26	130	6.4	6	17
29	135	6.8	3.8	17
28	150	5.9	5.9	19
26	150	5.95	4.8	18
22	130	6.95	4.5	17
30	165	6.1	5.2	18
27	130	7.1	4.3	18
32	120	7	4.9	17
32	150	5.9	5.8	18
27	150	6.8	4.2	17
20	140	6.84	4.7	16.5
23	165	6.18	4.75	19.5
38	155	5.93	6	19
27	114	7.4	5.38	17
23	124	6.56	3.62	19

28	143	5.68	4.9	17
20	148	6.03	5.81	19
27	126	6.4	5.34	12.5
27	150	7.34	5.24	21
20	130	6.66	6	19.5
20	130	7.15	5	20
30	140	7.53	4	21
20	110	7.38	5.2	19.5
20	150	5.97	5	20.5
20	110	6.59	5.2	18.5
20	90	9.62	5	15
20	110	7	4.5	22
40	135	6.28	5.1	19
30	151	6	5.25	16.5
27	114	6.56	5.7	12.5
23	116	6.78		10
24	143	6.43	4.7	20
29	161	5.9	4.55	17
29	140	6.53	5.77	13
25	160	5.81	5.7	22
30	165	5.4	5.36	18
23	142	6.25	6	19.5
25	130	6.28	4.1	18.5
30	151	5.5	5.6	17.5
20	120	7.31	4.83	18.5
30	151	6	5.08	21
30	160	6.54	4.5	25
27	182	6.34	5.94	25
34	180	5.38	5.83	26
20	146	6.78	4.5	28
36	180	5.03	6.56	29
34	182	5.06	5.5	29
34	194	5.3	6.75	31
40	156	4.6	7	35
34	180	5.08	6.94	24
20	170	5.94	6.5	24
30	155	6.31	6.1	24
23	165	6.8	4	24
24	160	6.37	5.99	24
14	115	6.43	4.3	12
22	121	6.56	5.2	16.25
26	140	6.03	6و5	18.5
24	99	7.09	5.1	18.5
21	120	5.56	5.36	13
20	125	7	4.65	18
25	130	7.4	5.51	12
40	150	5.65	6	23.5
40	160	6.66	5.35	19
27	178	5.24	5.5	18
40	200	6.16	6	22
31	168	5.24	4.57	23
30	160	5.316	4.67	15
30	143	6.3	4.8	16.5

45	165	6.45	6	22
30	155	5.83	5.25	19.5
35	113	6.58	5.1	16
25	120	6.06		18.5
35	200	6.88		17
35	185	5.22	5.22	21
23	134	5.02	5.27	16.5
30	180	4	5	21
30	173	5.4	5.78	18
34	176	5.07	6.3	22
32	162	5.2	6	20
34	141	5.12	4.8	
35	175	5.1	5.5	23
33	162	6	6.1	18
22	124	6.24	5.75	12
34	105	5.53	5.45	20
23	151	5.9	5.15	14
25	105	6.9	5.46	20
31	145	6.18	3.95	17
23	133	5.5	3.61	18
25		6.71	4.05	21
26	156	5.9	4.93	20
29	163	6.43	4.5	22
30	120	7.71	5.5	10
30	145	6.37	5.1	18.75
24	165	6.15	4.65	12
29	105	7.43	4	
30	104	6.68	4.89	13
27	144	5.96	4.45	17.75
24	134	6.78	5.75	17.5
23	135	5.93	4.43	22
33	158	5.56	5.07	16
22	110	7.93	3.91	18
37	150	7.59	6.35	16.25
33	165	6	4.53	13
37	154	6.71	5.8	20
27	142	5.9	5.51	13
26	119	6.81	5.28	
24	140	5.96	4.8	17
24	126	6.25	5.6	20
35	125	6.03	5.23	18.5
40	145	6.43	6	20
18	103	6.06	3.25	13
23	165	5.6	5.35	23.5
20	124	6.59	5.8	21
20	124	6.68	4.1	13
30	150	6.56	5.5	17
30	170	6.54	6.2	15
24	150	7.34	5	
23	165	5.91	5.1	18
28	155	6.15	5.6	16
24	160	6.1	5.1	17
20	140	7.03	5	19.5

20	120	7.47	5.1	10
20	130	6.72	6	15
34	148	5.44	5	19
26	168	5.61	5	19
28	143	6.14	6	22
30	134	5.95	5.5	23
20	160	6.31	5.5	23
20	110	7.63	4.3	19.5
20	150	7.81	5.5	18.5
20	110	7.53	5	19
30	140	8.57	5.5	19
30	160	6.63	5	18
20	130	7.73	5	19
30	160	6.69	5	18.5
30	130	7.47	5.5	18
20	110	7.78	5	18
20	155	6.6	6	22.5
20	90	8.35	3	10
20	120	7.44	6	20
20	120	7.72	5.5	10
27	168	5.5	6.15	18
30	140	7	6	23.5
20	120	8.25	4	10
20	130	7.199	4.75	23
30	130	7.31	5.5	17
20	140	6.78	5	20
20	150	7.25	6	21
30	140	6.65	4.35	19
20	120	7.5	5.2	17
30	150	6.31	5	22
20	130	7.75	6	20.5
20	110	7.53	4.2	20.5
20	110	7.4	5.2	18.5
30	120	6.91	5.5	18
32	156	6	5.5	17
24	157	5.45	5.46	20
30	140	6.84	4.5	21.5
30	150	7.06	4.5	18.5
30	160	6.85	5.5	22
20	140	6.63	5	21
30	130	7.15	6	18
20	160	7.71	6	22
25	140	6.98	3.82	21.5
27	140	6.9	4.4	21.5
28	100	6.8	4	21
25	130	6.81	4.8	23
27	165		5.05	23.5
26	157	5.46	4.9	16
24	150	6.05	5.85	14
30	150	6.44	4.2	15
28	155	5..38	6	21
26	145	7	6	21.5
24	125	7.4	5	

26	150	6.45	3.5	22
22	130	6.47	4.84	19
32	140	5.94	4.89	19
29	145	5.88	5.46	19
32	147	6.48	5.2	18
27	122	5.7	5.15	21
25	125	6.5	4.995	
22	160	6.84	4	19
23	140	7.02	5.1	
30	150	6.78	4.75	21
20	140	6.6	4.25	17
20	130	8.56	5.75	16.5
30	140	6.71	6	23.5
22	150	6.6	5.1	22.5
26	130	7	5	20
26	140	6.8	5	20
20	150	7.09	6	20
30	160	7.69	5.25	15
25	139	6.5	6	20
25	160	6.2	6.29	21
30	148	5.8	5.7	23
30	145	5.88	5.18	23
30	156	5.97	5.8	23
32	156	5.65	5.5	22
32	171	6.1	5.36	18
31	174	6.44	4.67	18
30	160	6.57	4.8	17
30	170	6.35	6	10
26	175	5.66	5.5	23
30	180	6.09	5.75	18.5
30	170	8.07	5	20
30	170	6.13	5	22
	181	5.75	5.8	18
27	170	6.06	6.12	22
29	175	6.06	5.2	19.5
40	170	7.28	5.7	21.5
37	175	5.68	5.6	21.5
35	190	5.63	6	22.5
29	186	5.63	6.25	23.5
20	180	6.44	6	10
20	180	6.31	6	10
22	170	6.4	6.25	17.5
25	165	6.62	5.4	20
20	170	6.98	5.2	
25	152	7.1	5.8	22.5
23	170	7.2	5.8	19
25	154	6.3	5	20
20	150	6.38	5.05	10.5
22	120	7.65	4.9	16.5
20	165	6.45	5	19.5
19	168	6.76	5.65	18
15	120	7.98	5.8	16
19	150	6.98	5.5	16.5

25	165	6.2	6	17.5
19	140	7.2	5.1	17
20	150	7.02	5.1	19.5
24	145	5.54	5.35	17
27	152	5.79	5.5	20
30	130	6.53	6	19
20	120	7.12	5.2	10
20	110	7.9	6	15
20	140	6.82	5	18.5
30	150	6.6	5.7	18.5
30	150	6.75	6.1	17.5
20	160	6.38	6	23.5
20	160	6.12	5.5	19
30	150	7.25	6	21
20	130	7.97	6	10
30	110	7.44	6	18
30	130	7	5	15.5
20	158	6.89	4.25	16
22	155	5.78	5.1	18
34	160	5.96	5.03	20
25	159	6.03	5.25	17
24	151	5.52	4.2	15
31	140	5.61	5.28	18
30	170	6.9	4.6	10
30	150	5.997	7	22.5
40	170	5.69	7	22
40	140	6.31	7	20
34	177	5.25	7	23
30	170	5	7	22.5
26	125	6.65	7.1	18
26	125	6.38	7.87	13
30	160	7.38	6.8	10
24	165	6.8	6.8	21.5
28	182	6.4	6.84	16
20	150	7.06	6.7	19.5
29	170	5.56	6.5	14
29	160	5.81	6.6	19
30	110	7.15	6.5	18.5
30	150	7.15	6.5	18
30	160	6.19	6.6	21
30	150	7.38	6.5	19.5
20	160	6.41	6.5	19.5
38	165	5.49	6.52	18
20	130	6.16	6.5	23
30	130	6.16	6.5	20
33	168	5.59	6.6	18
30	173	5.46	5.64	22.5
23	155	5.26	6.6	18
24	159	5.23	6.6	17
39	181	4.94	6.6	22
24	141	5.93	6.38	22
26	150	6.79	7	17.5
26	150	7.28	6	12

25	160	7	5	15.75
21	122	7.87	5	14.75
29	150	6.75	5	19
23	150	6.78	4.6	17
20	110	7.56	4.4	15
30	120	8.46	2.9	14
25	110	7.81	4.05	15
28	130	7.18	4	18
23	150	6.4	4.59	14
20	150	6.96	4.75	18
21	130	6.62	4.28	16
32	170	5.65	4.67	19
22	130	6.78	4.8	17
25	155	6.25	5.65	17
22	100	7.53	4.1	17
26	150	6.15	6	16
27	170	5.9	5.5	20
22	120	7.16	4.5	11.5
14	113	7.79	4.5	13
15	120	6.59	4.28	16
23	140	6.22	4.2	7
28	145	7.43	5.2	13
23	145	6.64	4.5	11.5
26	160	7.19	5.5	12
20	110	7.37	5	15.5
20	120	7.72	3.8	14
27	140	7.25	5.7	18
32	140	7.16	6	16
29	172	7	6	13
15	115	7.22	5.2	15
24	122	6.81	4	15.5
22	155	6.29	5.5	15
30	139	7.47	5.1	14
25	120	7.41	5.3	15.5
17	115	7.09	4	16.5
28	155	5.88	5.8	18
28	120	7.35	5.25	17
38	185	5.62	6.5	18.5
21	120	7.09	5.65	18
26	140	7.22	5	19
34	170	5.84	7	18
24	120	6.65	4.8	15
25	152	5.87	5.3	17
26	140	7.35	4.48	17
23	115	6.72	5.31	18
30	130	6.36	4.73	17
23	115	4.75	3.2	18
31	135	5.76	5.25	17
37	156	6.25	4.35	14.5
35	160	6.53	4.23	18
29	140	6.26	5.99	17.5
31	155	6.25	7	17.5
27	132	6.16	6.43	17.5

32	145	7.16	6.5	15.75
20	120	6.22	5	13.75
24	125	7.22	6.23	15.5
28	150	6.91	5.59	14.5
25	130	7.44	4.95	13.5
33	162	6.43	5.1	17
22	120	6.59	4.95	14
27	135	6.58	6	12
24	130	6.61	3.92	16.5
20	130	6.92	5.29	13
31	150	5.61	4.64	18.5
32	135	6.43	6.43	17.5
31	115	6.55	4.75	15
27	125	6.3	5.49	15.5
37	150	7.35	4.2	18.25
38	140	6.82	7	17
21	110	6.86	4.95	14.5
21	135	8.25	5.8	13
30	180	5.82	4.86	18
20	150	6.5	4.7	14
28	140	7.34	6.45	15
17	142	6.94	4	16.5
19	140	7.6	4.71	16.5
25	160	6.25	5.1	15
18	128	7.25	4.85	17.5
26	180	5.66	6	20
23	130	7	4.84	14
24	141	6.35	5.53	17
25	140	7.31	4.44	17
28	150	6.25	5.77	16
20	118	7.1	4.6	16
26	150	7.43	4.65	17
19	160	8.31	5.4	18
28	150	6.47	4.94	19.5
28	130	6.39	4.7	19.75
28	160	7.13	4.9	17.75
22	160	6.66	5	20.5
31	180	6.12	5.2	18.5
30	150	5.84	4.96	19.75
19	140	7.03	4.79	16.5
22	155	5.72	5.25	20.5
26	155	6.97	5.5	15.5
25	140	6.63	5.2	17.5
27	150	7.59	5.5	17
16	150	6.87	4.65	18.75
20	125	7.22	4.3	19.75
32	160	5.69	5.1	19.75
14	155	6.62	5.75	18.5
23	120	6.79	5.5	16.5
26	105	7.72	4	16.75
18	130	7.56	4.17	14.5
24	168	6.46	5.2	19.5
18	140	7.47	4.4	17.5

29	180	6.25	5.5	20
29	150	6.85	5.3	18.75
25	135	7.88	4.45	17.75
19	140	7.25	4.52	14.75
36	170	7.28	5.4	19.5
32	170	6	4.9	18.75
31	165	7.15	5.5	18.5
28	180	6.71	5.75	18.5
36	180	5.37	6.4	13
23	110	7.43	5.2	11.5
28	140	6.15	4.9	14.5
20	119	7.93	6.6	14
19	130	7.44	5.2	13
32	145	5.88	7	14
23	130	6.22	5.8	16.5
26	150	6.53	5.3	17
25	135	7.38	5.8	16
28	180	5.81	5.9	12.5
25	155	6.53	6	15
18	170	5.87	6	14
27	168	6.57	6.15	18
35	160	6.35	6.1	17
22	140	7.41	6	14
29	140	6.03	5.9	13.5
27	140	6.3	5.3	19.5
24	130	6.93	5.25	20
28	145	5.38	6.8	19
25	166	6.4	7.63	16
35	175	5.5	6.9	20
32	154	5.59	7.9	26
43	175	4.75	7.6	28
27	140	6.47	5.9	20
39	163	5.22	7.4	24.5
20	151	6.46	5.1	23
22	130	6.38	5.1	17.5
33	145	6.03	5.8	20.5
18	145	5.97	5.6	21
28	140	6.1	4.7	9
31	148	5.63	7	25.5
32	170	5.68	6.8	25.5
38	150	6.59	6.4	9
32	143	6.44	5.8	21
24	140	6.53	5.6	20
29	195	6	5.9	20.5
33	170	5.66	8.2	20
32	139	6.5	4.75	19.75
38	163	6.16	6	23
25	133	6.66	4.8	19
22	130	7.47	5.5	18.75
33	163	6.43	6.8	15.5
33	135	6.7	5.9	18.5
30	143	6.09	5.58	22
32	171	5.21	7.25	24.5

30	128	7.72	4.2	21.5
32	142	5.9	6.4	22.5
22	127	6.31	6	21.5
27	110	7.66	5.6	18.5
32	150	6.93	4.6	21
28	150	6.65	4.77	20.5
30	143	6.28	7.9	
28	135	7.3	6	18
26	140	7.34	6.19	
30	153	6.28	5.24	
30	160	6.66	6	22
31	150	6.94	5.56	22
35	140	6.44	5.86	22.5
31	180	5.9	6	24
28	125	7.25	5.8	24
31	173	6.44	5.64	23.5
24	145	6.84	6	
26	140	8.22	5	18.75
28	120	6.47	5	18
27	135	6.72	6.6	17.5
28	133	7.72	5	17.5
24	139	6.81	4.86	19
30	165	6.57	5	18.5
30	155	6.16	5.55	20
33	149	5.21	5.7	25.5
23	140	6.31	5.7	20.5
30	153	6.68	6.6	21.5
32	184	5.45	8	23.25
32	123	5.47	5.2	20.5
28	122	7.25	5.8	19.5
31	162	5.66	6.47	22.5
55	216	5.28	8.7	25.5
32	173	5.59	7.3	26
26	149	6.47	4.9	
27	138	6.78	5.5	
45	165	6.96	5.95	
30	125	7.97	5	18
25	140	6.8	5	19.5
30	152	6.31	5.14	19
33	150	7.25	5.58	17.75
25	140	6.6	5.63	17
12	129	7.9	5.6	19.5
28	169	6.66	6	20.5
33	130	7.72	4.73	16
31	130	7.5	5.86	16
35	148	5.64	6	26
30	144	6.21	5.5	21
35	156	5.66	5.58	23
20	133	7.5	5.58	17
30	179	5.94	5.6	20
25	145	7	5	21.75
25	150	6.22	5.5	25
40	173	5.69	7.3	20

25	130	6.72	5	
25	165	6.43	6.44	20.5
34	170	5.53	7.25	21.25
31	140	6.5	5	22.75
26	130	7.19	5.72	22.5
35	170	6	5.3	22.25
45	170	6	6.1	21
35	130	6.6	5.6	
25	140	6.19	6.19	20
45	180	5.6	6.91	21
35	180	5.22	6.44	24.5
40	180	5.75	7.23	22
20	130	6.81	6.47	16
15	131	7.15	6	17
34	159	6.47	5.66	19
25	138	6.5	5.31	21
32	168	5.6	5.18	23.5
25	90	8.22	4.54	18
30	163	5.88	5.37	19
30	160	5.84	5.29	20.5
35	127	6.78	4.57	18
30	155	5.56	5	23
35	157	5.9	5.66	24
30	136	6.94	5.5	25
35	140	5.85	5.3	14
26	165	6.34	5.53	23
30	140	6.14	5.43	19.5
39	180	5.88	7.2	
30	160	6.93	5.9	20
35	170	5.91	5.5	18
36	130	6.53	6	19
35	149	5.87	5.33	
23	130	7.66	6.6	21
34	169	5.5	6.2	20
25	120	7.53	5.32	19.5
25	138	7.4	4.83	18
27	130	7.93	5	21.5
29	135	7.71	4.6	12
28	112	7	4.27	15
28	130	6.15	4.77	14.5
35	160	7.04	5.7	16
24	140	6.81	5.11	17
30	165	6.34	5.85	18.5
37	153	7.03	5.12	17
31	126	6.72	4.6	16
25	140	6.57	4	
25	120	6.57	4.57	
26	120	7.94	5.53	17
30	134	6.19	5.3	20
25	104	5.98	5.5	22
33	165	6.25	6.5	22
34	135	6.91	6.1	21
25	159	5.53	6.2	

35	138	6.69	6	
35	175	5.87	6	19
20	120	6.85	5.52	
30	160	5.95	5	19.5
35	180	5.91	6	23.5
25	158	5.94	6.25	23.75
25	150	6.97	4.51	18.75
32	150	6.31	4.8	19
20	140	7.1	5.1	21
37	170	5.91	6.64	19
25	122	8.27	6.71	
26	110	6.75	5.37	
40	150	5.94	8	19.5
24	145	5.97	5.6	22
25	145	6.91	5.7	19.5
26	120	6.91	6	17
30	149		5	
34	150	5.97	6.65	17
47	173	5.34	6.8	12
28	130	6.82	6.4	20
33	170	5.22	7.5	21
35	149	5.94	7	19.5
42	173	5.72	7.8	22
38	150	6.13	7.7	19.5
34	150	5.88	6	20.75
27	164	5.94	4.4	19
26	118	7.38	5	19
28	135	7.12	5	17
33	139	7.22	4.57	18
35	150		5	16
25	140	7	4.37	18
30	140	6.43	5.3	18
30	170	5.47	6.3	15
31	132	6.44	4.8	17
24	143	6.28	5.3	19
25	120	6.75	5.63	8
25	178	5.28	6.8	
33	149	6	5.9	15
45	145	6.25	7.9	
42	163	5.19	7.6	18
23	150	5.9	6.16	21
23	140	5.81	5.7	22
27	130	5.91	5.72	18
25	131	6	5.6	18
53	146	5.61	7	24
17	114	6.7	6.43	13
25	123	6.06	5.51	21
27	122	6.49	5.62	15
31	122	6.2	6	17
30	138	5.75	7.2	19
24	121	6.9	5.3	14
32	110	5.9	4.9	15.5
25	120	5.93	5.05	16.5

24	102	6.45	4.99	19
24	119	6.03	5.3	19
25	127	5.45	6	16.5
22	115	6.41	4.3	17.5
30	123	6.2	6.9	16.5
30	123	5.75	5.95	17.5
30	105	8.9	6	9
21	100	7.4	5	15
20	105	6.09	5.9	20
24	115	5.94	4.8	17
24	135	5.9	6	19
27	155	5.4	6.9	7
30	110	7.7	5.7	15.5
26	130	5.34	5.15	18
27	135	6.25	6.2	15
26	119	6.13	6.1	18
21	128	6.04	6.77	18.5
27	130	5.59	6.6	14.5
33	113	6.01	5.85	19
27	135	6.3	5.2	18.5
29	123	5.83	7.83	14
26	134	5.6	7.7	16
27	133	5.72	5.71	18.5
29	162	4.8	7.09	23
30	100	5.5	6.4	20
34	131	5.65	4.9	18.5
23	120	5.4	5	23
27	135	6.4	5.61	20
26	131	6.23	5.15	16.5
23	145	6.4	5.78	21
21	127	6.3	5.93	15
31	131	5.5	6.22	16
31	134	5.4	5.48	17.5
24	140	5.91	5.76	17
33	140	6.45	6	18
27	150	5.98	6.48	12
26	150	5.81	6.42	14
34	155	5.34	7.21	18.5
28	105	7.82	6	12
21	130	7.5	5.56	14
29	130	6.7	6	17
34	145	5.6	6.2	13
22	120	6.7	6.25	9.5
25	140	5.7	6.7	16.5
16	110	6.53	4	10.5
25	120	6.09	5.4	9.5
27	131	6.44	5.39	13
35	142	5.93	5.6	16.5
30	140	5.61	6.01	18
35	160	5.37	5	21
24	135	6	5.1	17.25
27	120	6.01	4.56	16
28	129	5.3	5.6	21

27	128	5.1	5.47	22
24	100	6.24	4.48	19
23	130	5.78	5.5	18
29	90	6.97	4.68	18
25	127	5.4	5	20.5
32	165	5.38	7.1	22
19	120	6.07	6.02	17
27	145	5.3	5.02	10
33	172	5	6	23
23	120	5.44	4.1	18
29	135	5.01	6.12	15
28	135	5.9	6.07	21
20	110	6.3	4.89	17
20	115	6.3	5	16
23	145	5.9	6.1	20
23	120	5.6	5.98	20
34	170	5.15	6.2	21
25	130	5.75	5.1	17
23	120	6.15	5.2	17
21	110	4.96	6.4	17.25
25	105	6	5.11	14
26	120	5.11	6	17
27	115	5.42	4.6	21
20	120	5.9	5.97	21
28	145	5.7	5.45	22.5
23	115	6.9	6.15	11
23	120	6.8	6.3	16
32	130	7.27	6.9	20
36	170	6.04	6.4	15
24	140	7.1	6	13
33	122	7.22	6.9	14
34	160	5.39	7	20
27	120	5.38	6	14
22	100	5.9	5.05	16
24	121	5.09	5.6	15
23	127	6.63	6	22
21	110	6.1	4.65	15
35	130	5.1	6	22
24	126	5.5	6	16
20	120	6.47	4.7	15
21	112	5.7	5	15
23	120	6.2	5.9	17
28	115	6.25	6.3	19
22	120	6.09	5.32	16
30	121	6.27	5.24	19
27	105	6.59	4.15	15
23	122	6.69	5.29	17
27	130	6.5	5.6	21
30	135	5.43	5.25	22
16	110	7.27	5	17
26	120	5.41	6.07	15
27	137	5.5	5.25	18
20	124	6.71	6.4	16

23	178	5.01	4.3	20.5
23	113	5.2	5.84	19
22	108	6.7	5.2	12.5
23	150	5.5	6.03	22
25	140	5.39	4.57	20
26	130	5.1	6.49	20
20	139	5.6	6.7	17.5
22	110	6.5	5.77	20
33	140	6.1	5.4	22.5
10	124	6.2	4.33	17.5
29	150	6.9	5.85	19
22	130	6.12	4.3	16
25	110	6.47	4.93	15
17	105	6.43	5.9	17
25	123	4.49	5.07	15
25	100	7.1	4.52	19.5
20	115	6.03	4.2	9
24	130	5.3	5.05	16
14	100	7.4	4.64	14
24	130	5.7	6.9	20
25	109	6.7	5.27	18
10	104	7.5	4.7	15
34	133	6.19	5.7	18
24	157	6.62	5.9	19
56	118	7.5	6	15
27	140	6.01	5.77	15
24	140	5.37	7	17
35	157	5.39	6.7	19
24	140	6.03	6.7	17
43	117	7	4.4	11
10	105	7.4	4.69	15
17	120	6.47	6.1	19.5
34	155	5.6	7.5	20.5
27	101	7.5	5.7	15
25	120	6.1	4.65	17.5
27	155	5.6	4.77	20
24	115	6.7	4.97	19.5
31	127	5.66	6	20
41	165	4.6	7.45	22
27	115	6.11	5.55	19.5
39	140	5.1	7.05	19
24	121	5.93	5.9	18
33	150	5.15	5.13	22
23	124	6.22	5	13
24	138	5.61	5.49	20
21	104	6.01	4.93	13
23	139	5.91	5.51	15
28	117	6.25	5.1	19
22	133	5.75	5.59	20
28	129	5.09	6.81	14
25	134	5.47	4.19	19
25	120	5.3	5.7	19
28	116	5.69	6	17

24	117	5.7	6.78	20
19	116	5.9	5.72	20
27	136	6.06	4.35	20
20	113	6.84	5.41	17
23	142	5.25	5.81	19
25	105	6.23	6.3	19.5
24	137	5.6	6.14	18
29	155	5.34	7.66	19.75
35	160	5.34	6.35	
26	135	5.75	5.66	
29	145	6.39	8	
39	131	5.05	6.45	20
24	129	6.09	5.1	20
28	117	5.81	4.4	17
35	129	5.28	6.15	22
32	97	6.2	6	17.5
30	103	6.15	5.4	18
37	127	6.19	5.78	17
40	151	5.07	6.5	23
22	91	6.47	4	15
38	127	5.64	5.42	19
27	142	5.03	5.4	18.5
20	83	7.29	4.9	16
35	136	5.25	5.65	19
34	139	5.15	6.27	25
35	129	5.69	6.26	15
10	102	5.47	6.29	18.5
30	117	6.4	4.28	17
27	110	5.91	4.72	18
28	134	5.75	4.6	22
33	119	5.75	5.7	20
31	138	5.4	6.5	18
26	110	6	5.26	19
27	141	5.88	5.88	20
35	128	6.19	4.6	18
19	107	6.81	4.2	16
21	90	8.22	5.71	
36	147	5.28	6.9	19
25	112	6.69	4.5	15
30	119	5.56	5.28	22.5
43	138	5.04	7.1	28
30	121	6.37	6	15
31	144	5.91	5.8	17
33	126	5.59	6.55	19
40	128	5.59	6.78	24.5
34	135	5.75	5.78	24
26	90	6.28	5.7	21.5
27	105	6.25	4.42	19
48	171	4.89	6.34	20
30	115	6.3	5.55	20.5
28	100	6.06	5.11	19
24	122	6.21	4.85	21.5
35	134	6.15	5.75	21.5

31	111	6.25	5.25	19.5
19	121	6.25	6.65	21.5
35	86	7.16	6.35	16.5
28	125	6.68	5.07	20
26	105	9.57	7	
16	104	6.79	4.41	16
31	105	6.688	4.84	22.5
34	107	6.2	5.32	24
33	146	5.78	4.88	19
45	149	5.06	6.43	26
35	125	5.9	7.1	18.5
29	109	6.65	5.92	20.75
41	134	5.37	6.87	22
35	110	5.9	6.25	25
40	123	5.84	7.24	22.5
27	104	6.84	4.37	23
32	170	5.65	6.25	17.5
29	98	6.06	5.4	19
24	100	6.9	4.25	13.5
25	118	5.94	4.7	20
22	105	6.79	4.35	14
27	117	5.75	6	18
28	112	6.22	5.37	20
33	124	5.53	5.67	19.5
33	130	5.4	6.19	27
24	90	6.56	4.32	18
31	114	6.68	4.7	17
33	117	6.34	5	19.5
34	117	5.37	5.4	22.5
28	99	6.34	5	17
42	139	6.8	4.5	15
37	133	5.2	5.7	14.5
41	117	6.21	5.1	16.5
22	96	7.71	4.51	14.5
29	135	6.25	4.75	17
28	100	6.03	5.43	15.5
39	136	5.42	5.8	23
33	110	6.98	3.82	13.5
19	87	6.65	6	15.5
36	117	5.87	6	14
37	144	5.25	5.91	22
42	128	5.4	6	22
32	112	6.18	5.56	16
28	125	6.25	5.42	17
29	130	6.12	5.23	16.5
24	110	5.6	5	19.5
30	113	7.41	4.8	13.5
20	122	6.68	5.7	
31	102	5.94	4.75	10
37	129	6.3	5	19
39	105	6.31	5.9	19
30	124	5.5	5.7	21.5
31	107	6.28	5.3	12

30	123	5.93	5.1	19
32	111	6.59	4.45	18
32	112	6.1	7.12	19
29	106	6.16	6	17
34	109	6.22	5.3	19
22	137	5.91	5.6	22
29	92	6.77	5	20
36	116	5.97	5.7	15.75
43	130	5.43	5.65	19.5
33	131	5.8	8	21
29	100	6.06	4.9	19.25
18	81	8.03	3.51	12.5
50	160	5.03	8	21.5
49	131	5.66	5.8	23.5
39	121	5.72	4.9	23
38	136	5.5	6	20.5
29	139	5.85	7	24
31	116	5.63	5.4	18
23	90	7.45	5.1	14
37	105	5.31	5.3	19
40	136	5.45	5.65	20
34	111	6.35	5.8	17
26	123	6.09	5.6	17.5
36	119	5.66	6.8	19
32	101	6.19	6.1	19
33	124	7.09	4.32	16
33	115	6.02	5.8	20
40	113	6.09	5.75	20
18	90	8.5	4.8	17
40	110	5.28	6.9	22
39	118	5.87	5.7	18.5
37	134	5.19	7	21.5
25	101	6.16	4.4	20
31	102	6.16	6.1	19
30	92	6.69	5.6	19
47	148	5.15	6.35	24
27	101	6.53	5.9	17
28	106	6.25	5.1	20
34	128	5.67	4.8	17
36	130	6.59	4.9	20
31	91	6.41	5.5	18
34	117	6.44	5.5	20
37	135	5.69	6.5	18.5
42	121	5.25	6.4	18
31	120	5.72	6.6	20
32	120	6	5.2	14
34	104	5.44	6.5	20.5
20	104	5.94	5.65	17.5
17	108	5.59	6.5	19.25
43	105	5.84	5.2	19.5
36	120	5.6	5	20.25
38	115	5.54	5.2	20
22	86	6.72	4.5	20.5

24	117	6.35	5.65	16.5
29	121	5.72	5.5	21.5
22	90	8.38	5.2	15.5
40	143	5.59	6.3	22.5
37	141	5.63	5.45	18.5
37	149	5	6.75	22.5
56	163	4.85	8.45	26
36	122	6.44	6.8	20.75
39	159	5.21	7.7	20
31	114	5.34	5.28	21
29	128	5.97	6.2	17.5
27	101	5.91	4.9	19
35	147	5.5	6.8	21.5
25	100	6.34	5.1	20.75
30	142	6.1	5.11	20
35	136	5.22	5.3	19
32	143	5.42	5.6	19.5
32	103	6.66	4.23	12.5
29	127	6.69	5.6	18.5
41	127	6.25	4.35	20.5
32	109	5.38	6	20.5
41	122	5.87	5.2	
41	137	5.03	7.15	20
29	121	5.97	4.15	19.5
27	100	6.34	5.11	20
26	103	6.21	5.11	20
25	109	6.44	5.8	18.5
38	125	5.7	5.5	17.5
16	106	6.03	5	21
36	129	6.66	5.15	19.5
35	114	6.68	4.3	12
40	134	5.43	6.1	23
35	158	5.8	4.5	11
24	111	6.38	5.1	18
35	134	5.2	5.7	22
31	111	5.66	5.5	20
31	142	5.9	5.42	18
29	125	6.16	5.9	21.5
22	197	6.81	4.1	18.5
37	128	5.28	5.7	21
26	132	5.94	5.6	19.5
27	110	5.97	4.9	18.5
29	95	6.41	4.9	16
11	82	8.22	3	
32	102	6.34	5.2	18
27	118	6.22	5	21
25	105	6.09	5	18.5
32	96	5.47	6.3	17
27	93	7.09	4.5	19
38	117	5.69	4.7	16.5
37	109	5.91	7.6	20
22	96	6.53	5.8	15
36	97	5.72	4.1	21.25

44	125	5.27	5.9	19
33	120	5.46	4.9	18.5
30	113	5.56	4.75	18.5
24	109	5.41	5.65	22.5
33	138	5.97	5.8	20
42	115	5.5	6.1	19
37	130	5.41	5.9	22.5
39	155	5.03	6.8	26
43	141	5.46	5.9	22
32	119	6.56	4.77	18.5
27	102	5.89	5.49	19
36	138	5.34	6.1	21
20	97	6.31	5.5	19
33	118	5.75	5.84	20
31	118	6.89	6	18
38	120	5.5	5.9	21
30	101	5.81	6.55	20
30	107	5.87	5.45	22
30	97	6.31	4.7	18
24	101	8.12	4.14	17
12	90	6.96	3.75	18
25	111	6.49	4.4	21
40	168	6.25	6	20
36	189	5.12	7.45	23.5
29	150	7.03	6.43	21
35	146	6.69	5.6	19.5
30	120	6.3	6.66	22
39	143	6.28	5.77	20.5
30	150	6.18	6.35	21
25	126	6.15	4.34	17.5
34	135	6.82	5.4	17.5
31	124	6.07	5.8	22
23	140	6.15	5.73	20
29	147	6.57	5.8	20.5
32	150	5.28	6.1	19.5
33	145	6.62	5.4	22
29	147	5.91	6.1	20
38	167	6.64	6.14	20.5
28		5.66	5.6	
40	175	6.07	6.2	20
25	152	7.81	4.6	
33	142	5.66	6.1	18.5
46	124	5.37	7.83	20
43	162	5.8	4.4	21.5
28	160	7.75	4.2	18
32	163	6.1	5.9	21.5
37	128	6.12	5.5	18.5
26	131	6.11	5.5	19
17			4.9	
24				
28	151	7.2	6.69	21
31	148	6.28	5.9	18.5
33	143	6.37	5.1	19.5

39	180	5.87	6.89	20
39	173	5.41	6.1	20
33	167	5.56	7.5	21
28	151	6.16	5.5	22.5
34	120	6.25	6.6	19
33	180	5.7	6.5	18.5
34	137	6.6	6.6	21
26	124	6.22	5.8	18
49	173	6.18	6.8	20
26	137	7.07	5.7	18
32	167	6.32	6.5	21
40	154	6.07	5.9	19.5
35	168	5.29	6.2	20.5
28	133	7.28	5.5	20
34	153	6.16	6.1	18
33	140	6.28	6.28	21.5
26	148	6.79	5.1	20
49	186	5.01	6.6	23.5
34	180	5.34	6.9	22
23	184	5.45	6	22
30	128	6.82	5	22
28			6.7	20.5
30	135	6.92	6.1	17
40	132	6.25	6.1	20
31	140	5.66	4.9	19
44	192	6.23	6.71	24.5
44	190	6.23	6.65	24.5
29	129	6.71	6.22	19
23	128	7.06	5	19
33	170	5.79	6.8	16
36	172	6.27	6	18.5
37	154	6.18	5.7	22
21	132	7.35	6.5	21
19	163	6.68	7.54	15.5
26	131	7.3	6	20
33	135	5.99	6	20.5
25	155	6.98	6	
32	129	6.21	5.2	20.5
43	191	5.31	7.25	20
36	131	5.69	5.71	17
50	175	4.54	7.34	22
38	160	5.71	6.41	21
42	172	5	6.46	28
35	125	7	4.4	
32	155	7.59	6	15
24	145	5.1	5.9	20
33	175	5.81	6.2	19
24	144	6.41	4.5	20
30	140	6.18	6.5	21.5
33	130	6	5.1	16
27	140	6	6.15	22
35	144	6.37	5.63	19
47	160	5.45	5.84	24

27		7.02	5.6	17
36	129	6.4	4.9	19.5
	120	6.17	4.9	15
28	152	6.47	6.3	24.5
25	135	6.9	5.2	18.5
30	160	6.5	6.9	19.5
32	146	6.59	5.75	20.5
25	131	6.09	4.5	24
49	190	5.11	8.2	24.5
38	167	5.82	5.1	23
42	183	5.31	7	22
39	150	6	6.5	20
34	125	6.9	5.2	
10	100	8.26	4.7	15
29	135	6.71	4.8	20.5
33	150	6.35	6.2	20
25	115	6.7	6	20
24	120	6.86	5.6	18
29	155	6.39	7	19
28	115	6.19	5	19.5
18	155	6.35	6	25
20	120	6.71	5.1	18
62	125	5.67	5.1	20
25	120	6.14	4.8	19
20	135	6.38	4.8	20
24	120	6.27	6	20
17	105	6.94	5.1	11.5
25	150	6.85	5.2	19
19	130	7.21	5.1	14
49	180	5.57	7	17
25	135	7.07	4.65	18.5
20	125	7.66	3.6	16
17	120	7.09	3.2	17
29	130	6.74	7.2	20
30	125	7.17	4.2	18
32	152	5.33	5.65	19
42	190	6.1	6.25	21
33	120	6.65	7	20
25	100	7.28	5.25	15.5
26	125	5.7	6.45	19.5
25	105	6.03	5.6	17
25	135	5.96	7.1	23
39	125	6.22	5.75	19.5
20	122	6.46	6.45	14
25	110	6.72	5	17.5
20	120	6.99	6	21
31	130	5.01	4.95	18
29	125	5.85	5.8	18
30	135	5.02	5.45	18
27	115	7.78	5.05	16.5
25	120	6.03	5.95	18.5
38	152	5.43	5.67	21
24	122	6.45	5	19

21	90	7.17	6.3	18
22	122	5.78	5	18.5
26	125	6.67	5.3	17
35	165	5.94	6	20.5
30	127	5.08	4.7	17
39	147	6.24	6.25	20
28	123	6.27	5.35	18
31	137	6.63	4.95	
28	150	6.23	5	18.5
34	130	5.75	6.2	21.5
30	135	5.01	5	22
27	102	6.82	5.2	12
25	130	6.65	4.85	16
41	145	5.59	6	20.5
38	160	5.74	6	20.5
22	130	6.12	4.75	20.5
	112	7.53	5.8	18
22	140	6.22	4.5	18
32	175	7.25	4.55	19
23	125	5.45	6	18
25	135	6.9	5	15
29	275	5.99	5.45	18
25	269	6.1	5.45	17
13	100		3.1	13
28	130	6.44	5	18
23	145	6.74	6	16
29	120	6.72	5.1	23
28	125	7.22	5	16
25	150	6.19	4.8	23.5
20	105	6.37	6.25	20.5
33	145	6.32	5	19
30	155	5.69	7.35	23
37	145	6	5.5	20
24	115	6.97	4.3	15
30	155	6.12	6	17
25	135	6.31	5.8	21
15	120	8.82	5.05	20
30	120	6.5	5.95	20
20	240	6.59	5.2	18.5
25	200	9.7	3.6	18
30	160	6.57	5.9	18
25	135	5.78	6.1	26
28	140	6.12	4.5	19
29	135	6.07	5.85	19.5
35	150	5.85	6	18.5
28	135	6.53	4.5	22
24	260	6.92	5.15	17
29	102	7.16	5.65	20
43	160	5.4	6.1	21
30	127	6.46	5.5	20
30	180	5.44	5.7	20.5
36	125	5.04	6.3	22
30	140	6.03	6	26

30	110	6.02	6	17
31	160	6.06	6.5	19.5
30	130	5.8	6	19.5
29	130	6.72	5.8	12
27	95		5.2	
30	115	6.03	5.2	19.5
26	135	6	6.6	18
30	125	7	5.65	18
30	145	5.98	7	18.5
36	135	5.59	7.5	23.5
32	130	6.5	5.5	16
24	235	6.17	6	20
25	125	5.89	7	19.5
29	163	6.09	5.7	22
25	136	5.98	6	16.5
17	100	6.94	6.9	18
20	105	6.24	5	17
25	135	6.35	7.5	19
20	130	6.18	5.5	20
24	235	6.52	5.5	20
30	232	6.05	5.5	16
35	130	6.29	5.8	15
25	140	5.05	6.8	26
31	155	6.4	5.8	18
29	135	7.01	5	14
23	120	5.94	6	22
15	235	7.22	5.1	17
19	220	7	5.6	13.5
38	276	5.31	7.1	19
35	125	6.46	5.3	18.5
	115	7.75	6.5	20
	110	6.4	5.8	19
	112	6.69	5	21
	126	6.25	5.1	20
	128	6.45	6	19.5
	140	6.82	5.35	23
25	106	9.64	4.8	18.5
	112	6.63	6.2	19.5
30	120	6.02	6.35	19.5
30	110	7.08	5.05	15
29	195	5.82	5.95	
25	108	6.12	5.3	21
29	134	6.94	5	20
32	135	5.78	4.8	20
30	164	5.75	6.1	19
24	134	6.19	4.6	20.5
19	148	6.22	5	18
30	134	6.31	4.7	21.5
37	135	6.81	3.4	19
23	135	6.97	4.35	16.5
25	161	5.53	6.2	22.5
25	150	6.5	5.1	19
30	186	5.59	6.2	24

29	164	5.59	7	17
35	154	5.93	6.5	20
20		7.21	3.1	
30	148	5.66	4.9	23
27	124	6.44	4.9	20
35	154	6.26	5.6	20
31	149	5.35	4.8	22
35	149	6	6.15	15
31	138	6.07	5	
27	147	6.36	4.3	10
30	170	5.44	6.35	22
30	164	4.84	6	14
27	119	5.87	5.5	21
38	154	6.1	5.5	24
37	152	5.75	5.65	18
30	152	5.53	6	23
28	142	5.84	5.6	16.75
35	148	5.84	5.1	15
25	153	6	6.1	16.5
30	165	6.09	5.3	14.5
28	151	5.85	6.2	19.5
32	149	6.47	5	22
29	137	7	6.6	7.5
23	165	7.04	5.9	
23	120	6.54	5.4	16.5
36	178	5.28	6.6	24
24	148	6.34	4.7	18
23	118	6.87	5.2	25
31	195	4.87	6.4	19.5
28	152	5.09	5.7	31.5
25	105	7.03	4.1	
31	180	5.35	5.9	26.5
24	120	6.72	4.5	20
27	125	6.25	4.5	8.45
25	147	6.16	5.25	22.5
30	164	5.84	6.5	20
34	135	6	5.4	21
25	138	5.97	4.7	20
30	154	5.97	6.5	19
34	120		4.9	23
19	140		4	17.5
	134	6.87	5.1	
	120	7.09		
38	149	5.69	5.2	25
	105	6.25	5	
	150	5.6	7.33	
	124	6.25	4.8	
	148	5.63	4.7	
	130	7.31	5.5	
	184	5.1	6.35	
21	118	7.25	5.1	
14	119	6.56	3.6	19
35	110	6.68	5.1	19

24	110	6.84	5.39	19
15	128	7.13	4.9	15
23	132	4.9	5.67	11
18	130	6.78	6.38	
21	111	6.71	4.75	18
22	95	8.38		
37	131	7.01	5.4	15
31	135	6.34	5.98	17
35	130	6.18	6.55	18
30	132	6.12	5.8	17
31	140	6.65	5.74	
36	140	6.44	5.3	17
31	128	7.54	6.38	
22	128	8.03	5.26	
33	138	6.43	5.9	
28	120	6.96	5.23	8
10	90	8.03	3.57	
35	141	6.16	5.95	
39	148	6.25	5.78	8
34	135	6.06	6.45	
36	118	6.29	7.42	24
26	125	7.54	7.7	
25	160	6.97	6.23	23
22	130	6.12	4.47	14
21	125	6.28	5.81	
36	142	7.38	5.1	
25	130	6.28	5.42	8
44	184	5.5	5.9	10
29	145	6.22	4.82	11
35	162	5.43	5.83	21
48	210	4.62	6.2	24.5
39	208	4.91	7.3	
33	151	5.44	8.5	8
32	160	5.66	6.2	19
40	122	5.97	4.85	
23	121	6.5	6.6	16
19	112	8.5	5.45	20
25	119	6.91	5.1	15
28	142	5.68	5.35	20
17	90	6.87	4.1	
30	155	6.4	5.2	13
21	138	5.9	6.5	
22	110	6.9	4.4	9
17	111	7.38	4.5	21
21	109	6.44	5.5	12
51	162	6.29	6.5	22
40	166	5.37	5.6	12
30	140	6.56	4.95	9
30	159	5.97	5.1	
23	107		4.3	
26	138			
30	130	5.97	4.75	
27	125	6.25	5.4	

25	149	6.03	6.2	
32	128	5.68	5.65	11
22	110	6.25	4.75	10
26	109	6.56	5.5	
32	145	5.72	4.5	18
28	140	5.55	5.4	19
21	110	7.59	5.3	10
40	167	5.71	6.2	25.5
29	150	7.44	5.93	19
27	105	6.4	5.7	
35	142	5.75	5.85	24
29	118	6.59	5.68	22
20	118	6.32	5.6	22
24	112	7.2	6	
30	120	8.43	5.17	
26	130	7	5.1	20
29	135	7.25	5.75	
24	130	6.73	4.8	18
31	145	5.99	5.75	21.5
33	160	5.87	5.3	21.5
33		6.16	5	21.5
20		6.73	4.8	
30	112	6.75	4.7	20
22	115	8.06	6	
29	135	5.89	5.3	21
18	90	7.96	5	
25	109			
34	150			
26	110			
35	151			
28	129			
44	174			
42	145			
28				
25	110			
25	130			
32	159	6.3	6.9	23
30	168	6.8	5.8	17
30	163	6.8	6	15
30	173	6.9	5.3	16
16	106	8	5.1	11
15	120	5.62	4.4	19
23	170	5.31	6.6	20
14	123	6.05	4.63	14.5
21	127	5.78	5.35	18
13	121	7.1	4.35	14
21	132	6.84	5	17
10	100	8	2.4	
16	110	6.39	4.5	15
23	152	5.93	6.25	21
16	130	5.69	4.4	20
11	113	5.48	4.5	21.5
	143		4.1	

20	126	6.4	3.6	21.5
25	138	6.32	3.8	18.5
28	177	5.7	6.3	18.5
17	130	6.3	4.6	18.5
20	146	5.5	5.2	21.5
27	169	5.3	5.8	19.5
21	149	6.1	5.53	18.5
21	146	5.48	4	19.5
12	137	6.66	3.15	13.5
12	134	6.76	3.23	13.5
16	140	6.24	4.4	17
21	156	5.03	5.2	22
11	121	6.11	3.9	18
15	128	6.4	3.44	17
35	158	5.9	5.85	20
25	143	5.63	4.3	20
29	160	5.23	4.8	25.5
20	169	5.19	6	
19	144	5.67	5.65	18
18	128	5.93	5	19
18	144	5.75	5.6	22
22	147	5.9	4.7	20
22	144	5.87	5	17.5
16	134	5.73	5.5	16.5
26	137	5.89	5.5	18.5
26	148	6.37	5.1	17
20	145	6.08	4.3	17.5
30	128	5.74	5.15	20.5
15	103	6.4	3.45	14.5
21	131	5.89	3.6	19.5
16	130	5.93	4.85	21
23	149	5.5	5.85	20
15	125	6.7	3.21	18
33	170	5.6	5.6	21
20	129	6.1	4.9	21
27	145	6.39	4.2	17
29	203	4.76	5.5	21.5
14	122	6.47	5.32	17
18	137	6.79	5.57	17
25	130	6.77	5.3	18
22	168	5.55	6.28	24.5
26	193	5.19	6.37	24
29	187	5.46	6.4	23.5
20	151	5.97	6.29	15
26	137	5.44	6.34	19.5
22	132	6.34	5.06	16
43	174	6.17	8.91	20
13	146	6.32	6.39	19.5
26	131	5.87	5.7	18
16	141	6	5.41	15.5
23	170	5.47	4.79	24
24	157	5.87	6.56	17
35	194	5.22	7.79	24

14	111			16
17	132	6.28	4.57	19
26	187	5.42	5.13	15
30	169	5.16	7.14	14.5
22	149	5.97	5.78	13
28	191	5.26	5.84	20
22	112	6.09	3.28	15
25	123	6.57	6.1	17
33	100		6	
25	130		5.9	
25	150	6.1	6.7	
25	122	5.5	5	17.5
30	150	6.54	4.5	22
25	120	6.51	5	21
25	140	6.19	5.6	23
40	175	4.93	6.3	26
42	150	5.68	5.6	24
35	170	5.13	5.7	24
30	100	5.22	5	24
35	135	5.26	5.5	25
25	100	7.7	6.1	17
35	180		4.9	21
35	185	5.1	6	28.5
35	125	6.8	6.5	22
30	180	5.48	6.5	23.5
25	140	7.52	5	20
25	150	7.23	5.6	15.5
30	140	6	5.5	19.5
20	110	7.5	4.5	14
30	130	6.88	4.5	21
25	130	7.21	5.1	15.5
35	150	6.8	5	18.5
30	140	5.9	6.5	23.5
25	170	6.14	5.1	17
30	165	5.71	5.1	18
30	163	5.8	6.5	18
35	95	6.78	5	17
30	160	5	4.5	17.5
40	180	5.8	6.5	19
30	175	5.02	6.5	18.5
30	175	5.12	6	22.5
30	130	5.8	6.5	26
35	130	5.98	6.5	18.5
20	140	6.34	5.5	26
28	172	5.7	6.4	23.5
32	180	5.22	5.3	20
28	150	5.68	5.8	26
25	170	6.46	5.7	29
30	140	6.11	5.8	23
30	170	5.68	4.8	27
35	148	7.6	5.7	25
30	140	5.63	6.1	25
25	150	5.7	6.2	25

25	166	5.16	6.3	24
40	170	5	7.6	20
42	180	5.32	6.9	26
27	180	5.6	4.4	22
33	150	6.19	6.4	23
20	145	4.89	6.6	23
25	160	6.16	5.2	28
30	120	6.2	5.6	12
30	110	7.29	5.7	
30	145	7.95	5.8	21
20	115	7.49	5.6	
28	115	6.34	5	19.5
25	115	6.04	5.6	19
25	155	5.46	6.3	
35	140	6.4	5.5	16
32	172	5.7	5.5	25
30	170	5.09	4.6	19
32	140	6.3	5.3	
30	110	6.04	5.1	
25	140	6.41	5.9	
35	140	6.4	5.2	21.5
30	140	6.74	5.2	
20	130	8	5.6	
20	110		5.5	
25	140	5.72	5.2	
30	135		7.3	
40	130		5.6	
30			4.9	
25			5.23	25.5
20	120		5.1	
20	110		6.2	
30	140		7.3	
30	130	6.42	7.5	
40	160	6.03	6.3	
30	152	6.26	5	
30	145	7.7	5.5	
20	120	5.95	4.5	
40	160	6.15		21
33	100	6.3	7.2	
30	165	5.77	7	
30	110	6.37	5.8	
30	145	5.18	6.4	
30	170		6.2	
37	130		6.5	
38	150	6.17	6.3	
25	120	7.33	4.7	20
25	155	5.46	6.5	
28	172	5.7	6.4	23.5
36	125	6.2	6.8	
	140	6.08	6	26
28	130	6.09	6.4	
	160	6.14	6.1	14
28	150	6.4	6.6	20

35	188	5.5		9
30	86	5.42	4.5	15
30	155	4.8	6.5	27
30	143	5.89	4.5	17
25	135	5.82	8	23
20	150	5.74	5.5	18
20	100	6.02	4	8
20	110	4.98	7	16
20	123	5.85	5.5	19
35	115	4.9	4.5	17.5
35	170	5.14	7.5	18
25	135	5.8	6.5	19
35	185	5	7.5	16.5
30	130	5.22	4.5	17.5
30	180	6.12	6.5	19.5
50	120	6.15	7	17
35	125		7.5	18
35	160	5.12	6.5	27.5
25	128	5.9	5.5	26.5
40	180	5.02	8.5	28.5
25	135	5.3	5.5	16.5
30		6.13	4.8	25
30	172	5.7	5.2	19
30	155	5.78	5.3	25.5
25	145	5.89	6.2	25.5
30	140		5.5	
30	151	5.22	6.2	
30	140	6.48	5.8	29
28	140	6.26	6.7	
28	155	5.84	6.5	
39	205	4.95	7.5	
30	175	5.51	6.2	30
30	112	5.89	5.5	14
35	175	5.3	7.5	
20	110	6.5	5.4	22
25	142	6.02	7	18
35	180	4.48	6	30
28	130	6.48	4.18	21
48	160	5.64	4.53	10
33	150	7.25	5.75	
23	130	6.23	4.29	21
27	110	6.1	5.4	
27	160	5.24	6.5	28
26	130	5.15	5.5	23
35	170	4.81	6.7	26
30	170	6.48	5.5	24
30	152	6.52	5.6	23.5
25	140	6.66	5.7	22
35	195	5.65	5.5	27.5
34	150	5.46	5.1	23
28	160	5.51	5	24
58	180	4.5	8.1	20
15	140	6	5.7	29

32	140	6.14	5	28
30	160	5.79	5.2	29
35	190	5.76	5.7	24
35	180	5.2	5.85	24
30	180	5.4	7.2	24
30	150	5.24	5	25
28	152	5.78	5.9	24.5
37	150	5.7	5	21
39	144	5.5	6.16	21.5
35	170	5.84	7.58	18.5
35	140		5.95	19
38	155	6.28	5.6	24
31	130	6.33	5.3	21
37	170	5.41	7.4	26
45	175	5.17	6	26
29	142	6.03	5.51	
28	120	5.2	4.9	22
30	155	5.2	5.7	24.5
25	130	6.71	5.1	22
13	125	6.6	4.8	21
30	120	6.13	6.1	8
25	170	5.59	5.4	
25	155		6	
34	118	6.71	5.8	
32	140	6.2	5	19.5
28	165	6.18	5.7	25
20	125	5.45	5.6	24
30	155	5.7	5.8	
25	180	6.01	5.9	
30	185	5.82	5.9	
20	195	5.2	5.9	
31	160	6.4	5.6	21.5
34	130	6.5	5.6	20
38	150	5.77	5.8	19
34	140	6.02	5.6	17
40	150	6.56	5.5	30
22	135	6.5	5	15
28	125	6.41	5.7	18
25	150	5.12	4.4	
20	160	5.82	4.6	
25	150	6.31	5.5	
29	150	6.56	5.5	16
21	135		5.9	
31	160	6.21	5.5	
17	140	7.07	4.9	
25	130	6.47	5.8	11
25	140	6.26	5.8	
14	110	6.33	5.4	18
32	150	5.6	5.5	19.5
30	135	5.66	5.5	
30	150	5.27	6	
25	112	7.62	5.1	
39	175	5.52	5	

27	130	6.96	5.7	22
25	150	6.44	4.8	20
29	130	6	5.2	
31	140	7.7	5.2	11
30	170	6.3	4.8	22
43	140	6.01	5.9	
25	135		5	
30	150	5.57	5.5	17
20	120	6.34	5.1	
30	165		5.1	10
30	150	5.44	4.5	11
20	110	7.34	5.8	
38	183	5.31	6.5	
35	200	4.8	8.1	22.5
25	110	7.09	5	
20	145	6.21	4.8	
25	128	5.68	4.7	
30	128		5.1	
28	115	6.11	6	
35	210	5.24	5.4	
25	130	6.03	6.2	
24	150	5.6	7.1	9
18	150	6.11	6.6	19
37	160	5.57	6.5	8
25	120	5.6	6	21
30	142	6.29	6	
35	172	5.06	6.5	20
22	130	6.36	5.95	19
20	150	6.28	5.9	
35	160	6.09	6.2	
35	155	5	5.8	
25	140		6.9	
32	185	5.4	5.2	26
29	150	5.5	5.5	21.5
30	120	5.7	6	21
35	160	5.87	6.7	19.5
24	130	7.26	6.6	19
29	130	7.69	6.6	18
27	150		7.53	
25	153	7.18	6.5	
30	160	6.8	7	12
22	150	6.6	6.1	
	140		6.1	18
25	120		6.3	
40	160	5.3	6	22
30	130	6.3	5.8	18
29	150	5.61	5.6	20
37	140	6.37	6	
35	160	6.12	6.5	16
21	160	6.27	5.1	16
28	140		5.9	19
38	135	6.46	5.6	14
34	110	7.23	5.8	14

20	120		4.75	
30	130		6.5	
20	150		4.4	
20	110		6.04	10
30	140	5.29	5.1	
30	100		3.8	
25	150	6.67	4.5	
35	140	8.27	5.1	
30	140	8.1	4.5	
25	140	6.52	4.9	
27	140	7.2	4.7	20
39	150	6.2	6.67	
25	160	6.1	5.5	24
44	150	5.42	6.5	24
20	130	6.9	5.4	
32	130	7.53	5.75	
29	130	8.21	5.23	17
34	150	8.19	6.3	
25	110	7	6.5	
30	138	6.48	6.1	8
25	145	6.87	6.8	
28	170	5.23	7.1	23.5
40	130	5.22	5.1	
35	180	5.31	5.7	23
30	140	6.3	5.1	
25	160	6.31	6.1	
20	110		5	
25			4.94	
20	110		5.12	
30	110	6.14	4.5	18.5
20	100		3.2	
30	130		6.25	21.5
20	110		4.9	
20	110		6.1	
25	120	6.15	5	
25	140	5.41	4.7	
40	140	4.88	6.1	
30	150	5.5	5.5	
35	165	5.15	6.2	20
40	160	5.67	7	
32	160	4.69	6.5	26
35	180	4.74	6.8	25
25	120		5	
25	140	5.67	5.5	18.5
30	130	6.5	6.5	18
30	160	4.93	6	19.5
25	120	7.57	5.2	12.5
40	190	4.89	7.2	21.5
30	170	4.74	5.8	
25	160	5.41	6.5	24
26	150	5.87	5	15
35	155	6.73	5	15.5
25	130	6.79	5.7	17

25	135	6.6	6.1	17
30	140	7.44	4.59	18.5
20	150	7.48	5	15.5
30	160	6.35	5.8	18
25	130	7.44	4.9	17
30	130	6.81	6.8	
30	150	6.24	6	20
45	190	5.69	7.9	25
40	185	5.34	6.5	26.5
30	145	6.66	6.3	17
30	150	6.41	5.5	15.5
30	160	6.57	5.5	20
35	135	5.28	7.5	26
25	124	6	3.8	10
30	145	5.28	6.5	19.5
35	133	5.22	6.5	28
25	123	5.28	7.5	18.5
30	150	5.18	7.5	19.5
30	105	6.4	3.5	8.5
40	140	4.63	6	30.5
30	122	5.8	7	10.5
45	145	5.14	8	28.5
30	123	5.3	5.5	20
25	125	5.73	6.5	19.5
30	140	5.3	5.5	19
30	130	5.78	6.5	19
30	125	5.28	5.5	24
30	170	5.14	5.5	26
25	105	5.83	4.5	15
30	140	5.38	6.5	20
35	100	5.8	6.5	28.5
35	175	6	6.5	18.5
25	125	6	6.5	17
25	100			
35	180	5.08	6.5	30
35	150	5.63	6	18.5.
45	200	5.28	7.5	17.5
15			6.5	
35	145	5.18	6.5	17.5
30	185	5.62	6.5	25.5
30	135	5.9	5.5	22.5
45	120	6	6.5	19.5
35	110	5.43	6	24
30	185	5.3	5.5	27
45	175	5	6.5	23
35	195	4.88	6.5	26.5
40	200	4.83	6.5	35.5
30	125	5.63	6.5	24
35	135	6	5.5	24
40	185	5.1	5.5	26
30	189	5.14	6.5	27.5
35	135	5.8	5.5	18
35	115	5.08	5.5	23.5

40	180	5.18	6	25.5
35	145	6	6.5	13.5
35	175	5.8	6.5	26
22	140	5.63	6	18.5
45	150	5.12	6	20
30	155	5.02	6.5	14
35	150	5.9	6	19.5
40	105	6.08	6.5	18.5
25	144	5.8	7	19.5
25	120	6.3	5	15.5
25	135	7	5	
25	160	5.02	6.5	19
25	145	5.88	6.5	19
25	100	6	6.5	15.5
30	130	5.49	6.5	15.5
40	135	5.9	6.5	18.5
25	163	5	4.5	28
25	120	5.38	7	17.5
35	182	5	5.5	24.5
30	100	6.2	4.5	16
27	180	5.9	8	22
25	147	5.38	8.5	19
25	146	5	5.5	18.5
30	168	5	8	29
